# Supplementary material for: Applications to medical and surgical specialist training in the UK National Health Service, 2021–2022: a cross-sectional observational study to characterise the diversity of successful applicants
Source: BMJ Open. 2023 Apr 17;13(4):e069846. doi: 10.1136/bmjopen-2022-069846 (PMC10186087; doi:10.1136/bmjopen-2022-069846)
Supplement: Supplementary data [file bmjopen-2022-069846supp001.pdf]

Gender – All applicants

Gender	2021 All Applicants	2021 Accepted Applicants
Male	19340	5625
Female	17523	6480
Not Stated	1108	314

Country of Qualification – All applicants

Country of Qualification	2021 All Applicants	2021 Accepted Applicants
UK	17939	7987
Non UK	19044	4334
Non Medical Public Health Applicants	988	98

Ethnicity by Graduate Country

Ethnic Origin	Applied			Accepted		
	UK Grads	Non-UK Grads	Non-Medical Applicants	UK Grads	Non-UK Grads	Non-Medical Applicants
Asian or Asian British - Bangladeshi	164	481	12	85	103	0
Asian or Asian British - Indian	1733	2970	90	764	690	<5
Asian or Asian British - Pakistani	732	3175	36	291	675	<5
Chinese	968	173	13	353	50	<5
Any other Asian background	989	1299	33	368	315	<5
Black or Black British - African	453	3283	105	176	918	<5
Black or Black British - Caribbean	62	88	12	30	21	0
Any other Black background	16	346	6	7	66	0
Mixed White and Asian	400	305	19	179	64	<5
Mixed White and Black African	57	1101	20	21	190	0
Mixed White and Black Caribbean	66	31	5	31	7	0
Any other mixed background	244	393	13	109	87	<5
White - British	8982	119	440	4348	37	68
White - Irish	538	147	35	257	50	<5
Any other white background	796	1734	55	320	402	5
Any other ethnic group	423	1794	38	165	350	<5
Not stated	1316	1605	56	483	309	<5

Disability - All applicants

Disability	2021 All Applicants	2021 Accepted Applicants
Yes	464	179
No	36418	11940
Not Stated	1089	300

Specialty by Gender

Specialty and Level	Number of Applicants			Number Accepted An Offer		
	Male	Female	Not Stated	Male	Female	Not Stated
ACCS Anaesthetics/Core Anaesthetics CT1	1364	1026	67	323	315	10
ACCS Emergency Medicine ST1/CT1	806	576	42	165	186	9
ACCS Internal Medicine/Internal Medicine CT1	1772	1723	95	728	801	38
Allergy ST3	17	21	<5	<5	<5	0
Anaesthetics ST3	930	660	37	315	247	15
Audiovestibular Medicine ST3	11	5	<5	<5	0	0
Cardiology ST3	284	102	15	68	24	<5
Cardiothoracic Surgery ST1	69	40	<5	6	0	0
Cardiothoracic Surgery ST3	38	8	<5	<5	0	0
Chemical Pathology ST3	16	13	0	5	5	0
Clinical Genetics ST3	13	43	0	<5	14	0
Clinical Neurophysiology ST3	32	16	<5	7	<5	0
Clinical Oncology ST3	127	130	10	55	53	<5
Clinical Radiology ST1	1146	637	96	222	146	22
Combined Infection Training ST3	138	128	6	32	36	0
Community Sexual and Reproductive Health ST1	40	120	7	0	6	0
Core Psychiatry CT1	964	1092	74	258	398	27
Core Surgical Training CT1	1486	932	110	332	199	23
Dermatology ST3	63	218	12	<5	35	<5
Diagnostic Neuropathology ST3	8	8	0	0	<5	0
Emergency Medicine ST3	189	42	6	12	6	<5
Emergency Medicine ST4	87	49	<5	32	29	0
Gastroenterology ST3	187	63	<5	31	12	<5
General and Vascular Surgery ST3	380	215	12	71	60	0
General Practice ST1	4675	5128	250	1952	2532	96
General Psychiatry ST4	284	353	8	178	256	8
Genitourinary Medicine ST3	21	25	<5	<5	9	0
Haematology ST3	174	184	10	31	53	<5
Histopathology ST1	156	180	15	37	66	6

Immunology ST3	31	42	<5	<5	6	0
Intensive Care Medicine ST3	392	192	11	128	69	5
Medical Oncology ST3	91	113	8	30	45	<5
Medical Ophthalmology ST3	12	9	0	<5	<5	0
Neurology ST3	97	62	9	29	19	<5
Neurosurgery ST1	165	67	10	10	5	0
Neurosurgery ST2	41	17	<5	0	<5	0
Nuclear Medicine ST3	13	<5	0	<5	<5	0
Obstetrics and Gynaecology ST1	230	622	25	36	216	8
Obstetrics and Gynaecology ST3	94	215	<5	7	14	<5
Occupational Medicine ST3	26	19	<5	6	0	0
Ophthalmology ST1	355	238	32	49	34	6
Ophthalmology ST3	47	33	<5	8	7	0
Oral and Maxillo Facial Surgery ST1	31	19	<5	<5	5	<5
Oral and Maxillo Facial Surgery ST3	18	6	<5	8	<5	<5
Otolaryngology ST3	91	61	<5	19	13	<5
Paediatric and Perinatal Pathology ST3	<5	7	0	0	<5	0
Paediatric Cardiology ST4	24	10	<5	5	<5	0
Paediatric Surgery ST3	40	38	<5	<5	7	0
Paediatrics ST1	272	598	16	116	302	5
Paediatrics ST3	114	106	<5	11	12	0
Paediatrics ST4	93	82	0	11	16	0
Palliative Medicine ST3	31	70	<5	9	25	0
Plastic Surgery ST3	113	87	11	39	30	<5
Public Health Medicine ST1	315	644	29	33	63	<5
Rehabilitation Medicine ST3	45	29	<5	13	<5	0
Sport and Exercise Medicine ST3	26	7	<5	6	0	0
Trauma and Orthopaedic Surgery ST3	489	98	17	123	51	<5
Urology ST3	136	57	6	33	13	<5

Specialty by Disability

Specialty and Level	Number of Applicants			Number Accepted An Offer		
	Disabled	Not Disabled	Not Stated	Disabled	Not Disabled	Not Stated
ACCS Anaesthetics/Core Anaesthetics CT1	24	2373	60	6	631	11
ACCS Emergency Medicine ST1/CT1	9	1374	41	<5	351	8
ACCS Internal Medicine/Internal Medicine CT1	36	3457	97	17	1516	34
Allergy ST3	<5	37	<5	0	5	0
Anaesthetics ST3	19	1585	23	5	563	9
Audiovestibular Medicine ST3	0	16	<5	0	<5	<5
Cardiology ST3	<5	384	18	<5	90	6

Cardiothoracic Surgery ST1	<5	109	<5	0	6	0
Cardiothoracic Surgery ST3	0	44	<5	0	<5	<5
Chemical Pathology ST3	<5	26	<5	<5	9	0
Clinical Genetics ST3	<5	55	0	0	18	0
Clinical Neurophysiology ST3	<5	35	<5	<5	7	0
Clinical Oncology ST3	<5	260	6	0	107	<5
Clinical Radiology ST1	18	1785	506	7	369	14
Combined Infection Training ST3	<5	246	5	0	65	<5
Community Sexual and Reproductive Health ST1	<5	153	11	0	5	<5
Core Psychiatry CT1	57	2000	73	23	639	21
Core Surgical Training CT1	21	2410	97	8	519	27
Dermatology ST3	<5	277	12	<5	40	<5
Diagnostic Neuropathology ST3	<5	15	0	0	<5	0
Emergency Medicine ST3	<5	229	6	0	19	<5
Emergency Medicine ST4	5	130	<5	<5	57	<5
Gastroenterology ST3	0	248	6	0	42	<5
General and Vascular Surgery ST3	7	585	15	0	130	<5
General Practice ST1	121	9632	300	61	4412	107
General Psychiatry ST4	16	618	11	15	418	9
Genitourinary Medicine ST3	<5	47	<5	0	13	0
Haematology ST3	<5	358	9	0	83	<5
Histopathology ST1	11	330	10	6	101	<5
Immunology ST3	<5	73	<5	0	9	0
Intensive Care Medicine ST3	<5	582	10	<5	196	<5
Medical Oncology ST3	<5	206	5	<5	76	<5
Medical Ophthalmology ST3	0	21	0	0	<5	0
Neurology ST3	<5	155	11	0	47	<5
Neurosurgery ST1	<5	233	6	<5	15	0
Neurosurgery ST2	<5	55	<5	0	<5	0
Nuclear Medicine ST3	0	16	0	0	<5	0
Obstetrics and Gynaecology ST1	7	844	26	<5	250	6
Obstetrics and Gynaecology ST3	0	305	7	0	21	<5
Occupational Medicine ST3	<5	43	<5	0	6	0
Ophthalmology ST1	7	572	26	<5	86	<5
Ophthalmology ST3	<5	79	<5	0	15	0
Oral and Maxillo Facial Surgery ST1	<5	51	<5	<5	9	0
Oral and Maxillo Facial Surgery ST3	0	27	<5	0	15	0
Otolaryngology ST3	<5	149	<5	0	33	0
Paediatric and Perinatal Pathology ST3	0	11	0	0	<5	0
Paediatric Cardiology ST4	0	34	<5	0	6	0
Paediatric Surgery ST3	0	77	<5	0	8	<5
Paediatrics ST1	11	851	24	9	408	6

Paediatrics ST3	0	218	<5	0	25	0
Paediatrics ST4	0	174	<5	0	27	0
Palliative Medicine ST3	<5	99	<5	0	33	<5
Plastic Surgery ST3	0	206	5	0	71	<5
Public Health Medicine ST1	46	919	23	<5	93	<5
Rehabilitation Medicine ST3	<5	69	6	0	16	0
Sport and Exercise Medicine ST3	<5	34	0	<5	5	0
Trauma and Orthopaedic Surgery ST3	<5	588	13	0	175	<5
Urology ST3	<5	196	<5	0	48	0

Specialty by Ethnicity

Specialty and Level	Number of Applicants																	Number Accepted An Offer																
	Asian or Asian British - Bangladeshi	Asian or Asian British - Indian	Asian or Asian British - Pakistani	Chinese	Any other Asian background	Black or Black British - African	Black or Black British - Caribbean	Any other Black background	Mixed White and Asian	Mixed White and Black African	Mixed White and Black Caribbean	Any other Mixed background	White - British	White - Irish	Any other White background	Any other ethnic group	Not Stated	Asian or Asian British - Bangladeshi	Asian or Asian British - Indian	Asian or Asian British - Pakistani	Chinese	Any other Asian background	Black or Black British - African	Black or Black British - Caribbean	Any other Black background	Mixed White and Asian	Mixed White and Black African	Mixed White and Black Caribbean	Any other Mixed background	White - British	White - Irish	Any other White background	Any other ethnic group	Not Stated
ACCS Anaesthetics/Core Anaesthetics CT1	21	225	121	102	129	98	9	5	46	37	13	38	1135	79	162	77	160	<5	41	14	26	19	5	<5	0	9	<5	6	12	424	29	31	5	22
ACCS Emergency Medicine ST1/CT1	14	190	99	31	75	113	5	<5	33	33	<5	29	492	24	97	69	113	<5	18	9	10	6	12	<5	0	13	<5	<5	9	226	11	21	<5	15
ACCS Internal Medicine/Internal Medicine CT1	59	456	327	172	266	244	9	19	94	66	10	47	1062	79	269	161	250	26	192	111	79	112	91	<5	6	43	21	6	21	577	30	112	56	81
Allergy ST3	<5	7	6	<5	<5	6	0	<5	0	<5	0	<5	<5	0	<5	<5	5	0	0	0	<5	0	0	0	0	0	0	0	0	<5	0	<5	0	<5
Anaesthetics ST3	<5	193	38	49	56	18	<5	<5	39	20	<5	27	893	43	94	66	81	<5	58	<5	19	12	<5	<5	<5	14	<5	<5	12	350	20	33	12	31
Audiovestibular Medicine ST3	0	<5	<5	<5	<5	<5	0	<5	0	<5	0	0	0	0	<5	<5	<5	0	<5	0	<5	0	<5	0	0	0	0	0	0	0	0	0	0	0
Cardiology ST3	11	48	68	9	30	35	<5	<5	5	19	0	12	44	5	38	29	41	<5	15	7	<5	8	6	<5	0	<5	0	0	0	25	<5	10	0	11
Cardiothoracic Surgery ST1	<5	14	10	9	10	9	0	<5	5	<5	<5	0	10	<5	19	<5	16	0	<5	0	<5	<5	0	0	0	0	0	0	0	<5	0	0	0	0
Cardiothoracic Surgery ST3	<5	0	<5	<5	7	6	0	0	0	<5	<5	0	6	<5	<5	6	6	0	0	0	0	0	0	0	0	0	0	0	0	0	0	0	<5	<5
Chemical Pathology ST3	0	<5	<5	0	0	<5	0	<5	0	<5	0	<5	0	0	0	<5	0	0	0	<5	0	0	<5	0	0	0	<5	0	0	0	0	0	0	0
Clinical Genetics ST3	0	6	<5	<5	5	6	0	<5	<5	0	<5	0	17	<5	5	<5	<5	0	<5	0	<5	<5	0	0	0	<5	0	0	<5	8	0	<5	<5	0
Clinical Neurophysiology ST3	<5	<5	9	<5	<5	5	0	<5	0	<5	<5	<5	<5	<5	<5	<5	<5	0	0	<5	0	0	<5	0	0	0	<5	0	<5	<5	<5	<5	0	0
Clinical Oncology ST3	<5	32	23	7	14	10	0	<5	<5	7	<5	<5	60	<5	7	14	14	0	16	6	<5	6	<5	0	<5	<5	<5	0	<5	35	<5	<5	6	5
Clinical Radiology ST1	32	288	255	79	137	185	10	17	26	44	6	39	226	26	145	164	200	<5	57	29	25	22	18	0	<5	7	<5	<5	8	116	7	31	19	38

Combined Infection Training ST3	<5	28	33	5	16	30	<5	11	5	19	0	11	59	<5	10	5	19	0	7	5	<5	5	5	<5	<5	<5	<5	0	<5	28	0	<5	0	<5
Community Sexual and Reproductive Health ST1	5	22	15	<5	9	27	<5	<5	<5	<5	<5	<5	32	6	9	10	18	0	0	0	0	0	<5	0	0	0	0	0	0	<5	<5	0	0	0
Core Psychiatry CT1	48	234	249	36	125	358	8	23	40	86	7	40	363	17	150	158	188	14	76	41	15	27	93	<5	<5	19	12	<5	17	204	8	49	53	46
Core Surgical Training CT1	45	292	185	117	167	168	13	12	47	31	<5	42	694	56	243	147	265	9	75	34	26	40	26	<5	<5	15	<5	<5	7	189	11	42	26	48
Dermatology ST3	<5	43	36	25	20	18	<5	<5	8	6	<5	<5	48	9	24	24	22	<5	6	<5	<5	<5	<5	0	0	<5	0	<5	0	9	0	<5	5	5
Diagnostic Neuropathology ST3	0	5	0	0	<5	<5	0	<5	0	<5	0	<5	<5	0	<5	0	0	0	0	0	0	0	0	0	0	0	0	0	<5	0	0	<5	0	0
Emergency Medicine ST3	<5	88	19	<1	10	32	0	<5	<5	17	0	6	9	<5	11	15	19	0	5	<5	0	<5	<5	0	<5	<5	<5	0	0	<5	0	0	<5	<5
Emergency Medicine ST4	0	34	12	<5	<5	<5	0	<5	<5	<5	<5	0	53	<5	8	<5	7	0	9	<5	<5	<5	<5	0	0	<5	0	<5	0	33	<5	5	0	<5
Gastroenterology ST3	6	32	52	5	18	22	0	15	<5	19	<5	9	19	<5	11	21	18	<5	5	5	<5	<5	8	0	<5	0	<5	0	0	8	<5	<5	<5	<5
General and Vascular Surgery ST3	12	77	55	15	31	34	<5	8	9	29	<5	13	142	15	50	73	40	<5	9	5	<5	7	5	<5	0	<5	<5	0	<5	66	8	12	<5	6
General Practice ST1	236	1211	1474	213	725	1533	47	109	187	309	17	146	1834	169	517	568	758	104	530	568	96	304	681	25	44	67	125	7	64	1123	109	202	203	328
General Psychiatry ST4	8	107	39	21	28	68	<5	<5	7	6	0	7	210	12	65	30	32	<5	87	23	15	20	40	0	<5	<5	<5	0	6	153	6	38	21	21
Genitourinary Medicine ST3	<5	6	6	<5	<5	6	0	<5	<5	<5	0	<5	7	<5	<5	<5	6	0	<5	0	0	0	<5	0	0	<5	0	0	0	<5	<5	0	<5	0
Haematology ST3	7	59	39	13	12	37	<5	12	6	23	<5	12	57	5	24	30	30	0	20	<5	6	<5	<5	<5	0	<5	<5	<5	<5	30	<5	5	<5	<5
Histopathology ST1	<5	32	20	12	14	41	<5	6	8	12	<5	7	87	5	42	34	26	0	12	5	7	<5	5	<5	0	5	<5	0	<5	42	<5	10	6	8
Immunology ST3	<5	7	6	<5	<5	14	0	5	0	10	0	8	<5	<5	<5	<5	9	0	<5	<5	0	0	<5	0	0	0	0	0	<5	<5	0	0	0	<5
Intensive Care Medicine ST3	<5	72	29	20	19	18	<5	<5	12	15	<5	6	285	14	34	26	33	0	24	<5	6	<5	<5	0	0	6	<5	<5	<5	115	5	10	8	13
Medical Oncology ST3	<5	36	32	8	11	10	0	<5	<5	8	0	<5	48	5	10	14	21	0	14	13	<5	<5	<5	0	0	0	0	0	<5	22	<5	<5	<5	8
Medical Ophthalmology ST3	0	<5	6	0	<5	<5	0	0	<5	0	0	0	<5	0	<5	<5	6	0	0	<5	0	0	0	0	0	0	0	0	0	0	0	<5	0	0
Neurology ST3	6	19	27	<5	10	18	0	5	<5	13	<5	13	20	<5	13	9	16	<5	<5	<5	<5	<5	<5	0	<5	0	<5	0	<5	13	<5	7	0	6
Neurosurgery ST1	6	38	25	9	20	15	<5	<5	<5	5	<5	5	41	<5	31	11	26	0	<5	0	0	<5	<5	0	0	0	0	0	<5	6	0	<5	<5	0
Neurosurgery ST2	<5	<5	8	<5	<5	9	<5	0	0	<5	0	<5	<5	0	10	6	<5	0	0	0	0	0	0	0	0	0	0	0	<5	0	0	0	0	0

Nuclear Medicine ST3	0	<5	<5	0	<5	<5	0	0	0	0	0	0	0	0	<5	<5	<5	0	0	0	0	0	0	0	0	0	0	0	0	0	0	<5	<5	0
Obstetrics and Gynaecology ST1	15	85	43	25	52	146	11	6	19	27	<5	13	206	12	87	48	78	<5	20	7	6	17	21	5	<5	11	<5	<5	<5	114	5	18	7	20
Obstetrics and Gynaecology ST3	<5	79	41	0	8	46	<5	9	6	23	<5	5	<5	0	27	34	22	0	13	<5	0	<5	<5	0	0	0	0	0	0	0	0	<5	<5	<5
Occupational Medicine ST3	0	<5	<5	0	<5	6	0	<5	0	<5	0	0	7	0	<5	0	<5	0	0	0	0	0	<5	0	0	0	0	0	0	<5	0	<5	0	<5
Ophthalmology ST1	7	74	72	30	51	37	<5	<5	7	14	<5	13	72	15	66	68	71	0	13	<5	5	6	<5	0	0	<5	0	0	<5	24	<5	12	13	6
Ophthalmology ST3	0	18	<5	<5	<5	6	<5	0	<5	<5	0	<5	<5	0	9	16	14	0	<5	<5	0	0	<5	<5	0	0	0	0	0	0	0	<5	5	<5
Oral and Maxillo Facial Surgery ST1	<5	12	<5	0	<5	<5	0	0	<5	<5	0	<5	13	<5	5	5	<5	0	<5	0	0	<5	0	0	0	<5	0	0	0	<5	<5	0	<5	0
Oral and Maxillo Facial Surgery ST3	0	<5	<5	<5	<5	0	0	0	0	0	0	0	6	0	<5	<5	<5	0	<5	0	<5	0	0	0	0	0	0	0	0	<5	0	<5	<5	<5
Otolaryngology ST3	29	<5	<5	9	14	<5	0	0	5	<5	<5	<5	47	<5	15	12	10	0	5	<5	<5	<5	0	0	0	0	0	0	<5	18	0	<5	<5	<5
Paediatric and Perinatal Pathology ST3	0	<5	0	0	<5	0	0	0	0	0	0	0	0	0	0	0	0	0	0	0	0	<5	0	0	0	0	0	0	0	0	0	0	0	0
Paediatric Cardiology ST4	0	<5	<5	<5	0	<5	0	0	<5	<5	0	0	8	0	6	8	5	0	0	0	<5	0	0	0	0	0	<5	0	0	<5	0	0	<5	<5
Paediatric Surgery ST3	<5	7	<5	<5	0	<5	0	0	<5	<5	0	6	25	<5	6	13	5	0	<5	0	0	0	0	0	0	0	0	0	0	5	0	<5	<5	<5
Paediatrics ST1	6	107	36	34	52	82	<5	10	10	31	0	8	311	22	69	44	62	<5	52	9	17	18	25	<5	<5	7	8	0	5	203	14	23	15	19
Paediatrics ST3	<5	51	22	<5	6	30	<5	8	5	28	0	5	10	<5	9	23	17	0	11	<5	0	0	<5	0	0	0	<5	0	0	<5	<5	<5	0	0
Paediatrics ST4	<5	49	13	<5	5	20	0	5	8	20	0	<5	7	<5	8	23	9	0	9	<5	<5	<5	<5	0	0	<5	<5	0	<5	<5	0	<5	<5	<5
Palliative Medicine ST3	<5	10	6	6	7	5	<5	0	<5	<5	0	0	49	<5	<5	<5	<5	0	<5	<5	<5	0	0	0	0	0	0	0	0	27	<5	0	0	0
Plastic Surgery ST3	0	24	7	16	11	6	<5	<5	<5	<5	<5	5	60	<5	24	13	33	0	<5	<5	6	5	<5	0	0	0	<5	<5	<5	28	<5	8	<5	9
Public Health Medicine ST1	12	90	36	13	33	105	12	6	19	20	5	13	440	35	55	38	56	0	<5	<5	<5	<5	<5	0	0	<5	0	0	<5	68	<5	5	<5	<5
Rehabilitation Medicine ST3	<5	<5	13	<5	5	13	0	<5	<5	6	0	<5	9	<5	<5	5	9	0	<5	<5	0	0	<5	0	<5	0	<5	0	<5	0	0	<5	<5	<5
Sport and Exercise Medicine ST3	0	5	6	0	<5	<5	0	0	<5	0	0	0	11	<5	<5	<5	<5	0	<5	<5	0	<5	0	0	0	0	0	0	0	0	<5	0	0	0
Trauma and Orthopaedic Surgery ST3	<5	113	44	12	23	31	<5	<5	8	21	<5	11	177	7	41	45	58	<5	17	12	6	6	<5	<5	0	0	<5	<5	<5	98	<5	8	5	12
Urology ST3	6	22	13	10	9	18	<5	<5	<5	<5	0	<5	59	8	12	20	12	<5	9	<5	0	<5	<5	<5	<5	<5	0	0	0	23	<5	<5	<5	<5
