# Supplementary material for: Applications to medical and surgical specialist training in the UK National Health Service, 2021–2022: a cross-sectional observational study to characterise the diversity of successful applicants
Source: BMJ Open. 2023 Apr 17;13(4):e069846. doi: 10.1136/bmjopen-2022-069846 (PMC10186087; doi:10.1136/bmjopen-2022-069846)
Supplement: Supplementary data [file bmjopen-2022-069846supp002.pdf]

Supplementary Tables

Specialty and Level of Entry	Group
ACCS Internal Medicine/Internal Medicine CT1	Acute
ACCS Anaesthetics/Core Anaesthetics CT1	Anaes/ICU
Anaesthetics ST3	Anaes/ICU
Intensive Care Medicine ST3	Anaes/ICU
ACCS Emergency Medicine ST1/CT1	EM
Emergency Medicine ST3	EM
Emergency Medicine ST4	EM
General Practice ST1	GP
Allergy ST3	Medical
Audiovestibular Medicine ST3	Medical
Cardiology ST3	Medical
Clinical Genetics ST3	Medical
Clinical Neurophysiology ST3	Medical
Clinical Oncology ST3	Medical
Combined Infection Training ST3	Medical
Dermatology ST3	Medical
Gastroenterology ST3	Medical
Genitourinary Medicine ST3	Medical
Haematology ST3	Medical
Immunology ST3	Medical
Medical Oncology ST3	Medical
Medical Ophthalmology ST3	Medical
Neurology ST3	Medical
Occupational Medicine ST3	Medical
Palliative Medicine ST3	Medical
Rehabilitation Medicine ST3	Medical
Sport and Exercise Medicine ST3	Medical
Community Sexual and Reproductive Health ST1	Obs/Gynae
Obstetrics and Gynaecology ST1	Obs/Gynae
Obstetrics and Gynaecology ST3	Obs/Gynae
Ophthalmology ST1	Ophthalmology
Ophthalmology ST3	Ophthalmology
Paediatric Cardiology ST4	Paediatric Medical
Paediatrics ST1	Paediatric Medical
Paediatrics ST3	Paediatric Medical
Paediatrics ST4	Paediatric Medical
Chemical Pathology ST3	Pathology
Diagnostic Neuropathology ST3	Pathology
Histopathology ST1	Pathology
Core Psychiatry CT1	Psychiatry
General Psychiatry ST4	Psychiatry
Public Health Medicine ST1	Public Health
Clinical Radiology ST1	Radiology
Cardiothoracic Surgery ST1	Surgical
Cardiothoracic Surgery ST3	Surgical
Core Surgical Training CT1	Surgical
General and Vascular Surgery ST3	Surgical
Neurosurgery ST1	Surgical
Neurosurgery ST2	Surgical
Oral and Maxillo Facial Surgery ST1	Surgical
Oral and Maxillo Facial Surgery ST3	Surgical

Otolaryngology ST3	Surgical
Paediatric Surgery ST3	Surgical
Plastic Surgery ST3	Surgical
Trauma and Orthopaedic Surgery ST3	Surgical
Urology ST3	Surgical

Supplementary Table 1: Grouping of individual specialty posts for onward analysis
Abbreviations: ST, Specialist Trainee (year); CT, Core Trainee (year); ACCS, Acute Care Common Stem.

Specialty and Level of Entry	Male	Female	Not Stated	Total
General Practice ST1	4675	5128	250	10053
ACCS Internal Medicine/Internal Medicine CT1	1772	1723	95	3590
Core Surgical Training CT1	1486	932	110	2528
ACCS Anaesthetics/Core Anaesthetics CT1	1364	1026	67	2457
Core Psychiatry CT1	964	1092	74	2130
Clinical Radiology ST1	1146	637	96	1879
Anaesthetics ST3	930	660	37	1627
ACCS Emergency Medicine ST1/CT1	806	576	42	1424
Public Health Medicine ST1	315	644	29	988
Paediatrics ST1	272	598	16	886
Obstetrics and Gynaecology ST1	230	622	25	877
General Psychiatry ST4	284	353	8	645
Ophthalmology ST1	355	238	32	625
General and Vascular Surgery ST3	380	215	12	607
Trauma and Orthopaedic Surgery ST3	489	98	17	604
Intensive Care Medicine ST3	392	192	11	595
Cardiology ST3	284	102	15	401
Haematology ST3	174	184	10	368
Histopathology ST1	156	180	15	351
Obstetrics and Gynaecology ST3*	94	215	<5	309
Dermatology ST3	63	218	12	293
Combined Infection Training ST3	138	128	6	272
Clinical Oncology ST3	127	130	10	267
Gastroenterology ST3*	187	63	<5	250
Neurosurgery ST1	165	67	10	242
Emergency Medicine ST3	189	42	6	237
Paediatrics ST3*	114	106	<5	220
Medical Oncology ST3	91	113	8	212
Plastic Surgery ST3	113	87	11	211
Urology ST3	136	57	6	199
Paediatrics ST4	93	82	0	175
Neurology ST3	97	62	9	168
Community Sexual and Reproductive Health ST1	40	120	7	167

Otolaryngology ST3*	91	61	<5	152
Emergency Medicine ST4*	87	49	<5	136
Cardiothoracic Surgery ST1*	69	40	<5	109
Palliative Medicine ST3*	31	70	<5	101
Ophthalmology ST3*	47	33	<5	80
Paediatric Surgery ST3*	40	38	<5	78
Rehabilitation Medicine ST3*	45	29	<5	74
Immunology ST3*	31	42	<5	73
Neurosurgery ST2*	41	17	<5	58
Clinical Genetics ST3	13	43	0	56
Oral and Maxillo Facial Surgery ST1*	31	19	<5	50
Clinical Neurophysiology ST3*	32	16	<5	48
Cardiothoracic Surgery ST3*	38	8	<5	46
Genitourinary Medicine ST3*	21	25	<5	46
Occupational Medicine ST3*	26	19	<5	45
Allergy ST3*	17	21	<5	38
Paediatric Cardiology ST4*	24	10	<5	34
Sport and Exercise Medicine ST3*	26	7	<5	33
Chemical Pathology ST3	16	13	0	29
Oral and Maxillo Facial Surgery ST3*	18	6	<5	24
Medical Ophthalmology ST3	12	9	0	21
Audiovestibular Medicine ST3*	11	5	<5	16
Diagnostic Neuropathology ST3	8	8	0	16

Supplementary Table 2: The number of applicants to Specialty Posts in the 2021-2022 recruitment cycle by gender. Totals with * are not exact as <5 applicants did not state their gender. ‘Nuclear Medicine ST3’ and ‘Paediatric and Perinatal Pathology ST3’ did not have 5 or more applicants in a gender category and therefore are not included here. Abbreviations: ST, Specialist Trainee (year); CT, Core Trainee (year); ACCS, Acute Care Common Stem.

Specialty and Level	Male Success (%)	Female Success (%)	Sig diff in male or female success (p-value)
Emergency Medicine ST3	6.35	14.29	0.107
Neurosurgery ST1	6.06	7.46	0.769
Cardiothoracic Surgery ST1	8.70	0.00	0.084
Community Sexual and Reproductive Health ST1	0.00	5.00	0.338
Occupational Medicine ST3	23.08	0.00	0.032
Sport and Exercise Medicine ST3	23.08	0.00	0.301
Chemical Pathology ST3	31.25	38.46	0.714

Supplementary Table 3: Success of applicants to Training Posts by specialty in the 2021 recruitment year by gender where numbers are small. Table showing the applications of trainees to Specialty Training Posts and percentage of successful applicants by gender, where numbers are small and *p*-values derived from Fisher exact tests are provided for comparison and to 3 decimal places. Where p-values fall below the significance threshold of 0.05 they are highlighted in blue (Occupational Medicine ST3, which had a higher proportion of successful males).

Ethnic.Origin	Total	Non−UK Graduate Success (%)	UK Graduate Success (%)	Percentage Difference (95% CI)
Asian or Asian British − Bangladeshi	645	21.4	51.8	30.40 (21.50 to 39.30)
Asian or Asian British − Indian	4703	23.2	44.1	20.90 (18.00 to 23.70)
Asian or Asian British − Pakistani	3907	21.3	39.8	18.50 (14.60 to 22.40)
Chinese	1141	28.9	36.5	7.57 (−0.18 to 15.30)
Any other Asian background	2288	24.2	37.2	13.00 (9.06 to 16.90)
Black or Black British − African	3736	28.0	38.9	10.90 (6.02 to 15.80)
Black or Black British − Caribbean	150	23.9	48.4	24.50 (7.85 to 41.20)
Any other Black background	362	19.1	43.8	24.70 (−3.25 to 52.60)
Mixed White and Asian	705	21.0	44.8	23.80 (16.80 to 30.70)
Mixed White and Black African	1158	17.3	36.8	19.60 (5.94 to 33.20)
Mixed White and Black Caribbean	97	22.6	47.0	24.40 (3.00 to 45.80)
Any other mixed background	637	22.1	44.7	22.50 (14.70 to 30.30)
White − British	9101	31.1	48.4	17.30 (8.51 to 26.10)
White − Irish	685	34.0	47.8	13.80 (4.58 to 22.90)
Any other white background	2530	23.2	40.2	17.00 (13.00 to 21.10)
Any other ethnic group	2217	19.5	39.0	19.50 (14.40 to 24.60)
Not stated	2921	19.3	36.7	17.40 (14.10 to 20.80)
All Ethnicities*	36983	22.8	44.5	21.80 (20.80 to 22.70)

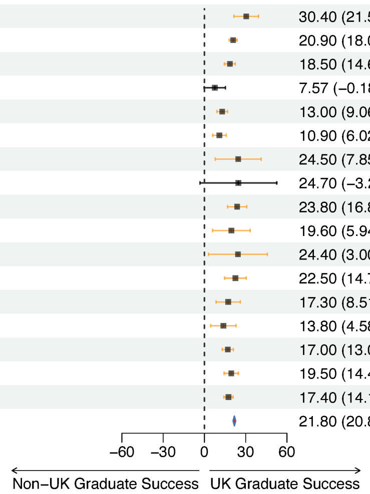

Supplementary Table 4: Success of applicants to Training Posts by ethnicity in the 2021 recruitment year by graduate status. Table showing the applications of trainees to Specialty Training Posts by ethnicity and percentage of successful applicants by graduate status (UK vs Non-UK). All values are provided to 3 significant figures. Where confidence intervals for estimated differences between the percentage of successful applicants by graduate status does not cross zero, it is highlighted in orange (greater UK graduate success).

Predictors	Adjusted Odds Ratios	95% Confidence Interval	p
Non-UK Graduates	0.43 ***	0.41 – 0.46	**<0.001**
White – Irish	1.02	0.87 – 1.20	0.762
Any other White background	0.73 ***	0.66 – 0.81	**<0.001**
Mixed White and Black Caribbean	0.88	0.58 – 1.33	0.546
Mixed White and Black African	0.52 ***	0.44 – 0.61	**<0.001**
Mixed White and Asian]	0.78 **	0.66 – 0.92	**0.004**
Black or Black British - Caribbean	0.88	0.61 – 1.23	0.453
Black or Black British - African	0.91	0.83 – 1.00	0.058
Any other Black background	0.60 ***	0.45 – 0.77	**<0.001**
Chinese	0.65 ***	0.57 – 0.74	**<0.001**
Asian or Asian British - Bangladeshi	0.80 *	0.67 – 0.96	**0.016**
Asian or Asian British - Indian	0.79 ***	0.72 – 0.85	**<0.001**
Asian or Asian British - Pakistani	0.68 ***	0.61 – 0.74	**<0.001**
Any other Asian background]	0.71 ***	0.64 – 0.78	**<0.001**
Any other mixed background	0.77 **	0.64 – 0.92	**0.004**
Any other ethnic group	0.62 ***	0.55 – 0.69	**<0.001**
Not stated	0.60 ***	0.55 – 0.67	**<0.001**
Observations	36983		

Supplementary Table 5: The effect of ethnicity and graduate status on success at application to Specialty Posts in the UK. Multivariable logistic regression model comparing success of application to Specialty Training Posts by minority ethnic groups to White British applicants where Country of Qualification (UK versus Non-UK) is included as a covariate. Non-Medical applicants to Public Health Specialty Training have been removed (n=988). Odds ratios are presented with 95% Confidence Intervals. ** p<0.05   ** p<0.01   *** p<0.001.*

Predictors	Unadjusted Odds Ratios	95% Confidence Interval	p
Non-UK Graduates	0.37 ***	0.35 – 0.38	**<0.001**
White - Irish	0.87	0.75 – 1.02	0.089
Any other white background	0.43 ***	0.39 – 0.47	**<0.001**
Mixed White and Black Caribbean	0.69	0.46 – 1.04	0.079
Mixed White and Black African	0.24 ***	0.20 – 0.28	**<0.001**
Mixed White and Asian	0.57 ***	0.48 – 0.66	**<0.001**
Black or Black British – Caribbean	0.55 ***	0.39 – 0.77	**0.001**
Black or Black British - African	0.45 ***	0.41 – 0.48	**<0.001**
Any other Black background	0.27 ***	0.21 – 0.35	**<0.001**
Chinese	0.59 ***	0.52 – 0.67	**<0.001**
Asian or Asian British - Bangladeshi	0.44 ***	0.37 – 0.53	**<0.001**
Asian or Asian British - Indian	0.48 ***	0.45 – 0.52	**<0.001**
Asian or Asian British - Pakistani	0.35 ***	0.32 – 0.38	**<0.001**
Any other Asian background	0.46 ***	0.41 – 0.50	**<0.001**
Any other mixed background	0.48 ***	0.40 – 0.57	**<0.001**
Any other ethnic group	0.33 ***	0.29 – 0.36	**<0.001**
Not stated	0.40 ***	0.37 – 0.44	**<0.001**
Observations	36983		

Supplementary Table 6: The unadjusted effects of ethnicity and graduate status on success at application to Specialty Posts in the UK. Unadjusted multivariable logistic regression model comparing success of application to Specialty Training Posts by minority ethnic groups to White British applicants where Country of Qualification (UK versus Non-UK) **is not** included as a covariate. Non-Medical applicants to Public Health Specialty Training have been removed (n=988). Odds ratios are presented with 95% Confidence Intervals. ** p<0.05   ** p<0.01   *** p<0.001.*

Ethnicity	UK Graduate probability of success	95% Confidence Interval	Non-UK Graduate probability of success	95% Confidence Interval
White British	0.48	0.47-0.49	0.31	0.23-0.40
White Irish	0.48	0.44-0.52	0.34	0.27-0.42
Any other white background	0.40	0.37-0.44	0.23	0.21-0.25
Mixed White and Black Caribbean	0.47	0.35-0.59	0.23	0.11-0.40
Mixed White and Black African	0.37	0.25-0.50	0.17	0.15-0.20
Mixed White and Asian	0.45	0.40-0.50	0.21	0.17-0.26
Black or Black British Caribbean	0.48	0.36-0.61	0.24	0.16-0.34
Black or Black British African	0.39	0.34-0.43	0.28	0.26-0.30
Any other Black background	0.44	0.22-0.68	0.19	0.15-0.24
Chinese	0.36	0.33-0.40	0.29	0.23-0.36
Asian or Asian British Bangladeshi	0.52	0.44-0.59	0.21	0.18-0.25
Asian or Asian British Indian	0.44	0.42-0.46	0.23	0.22-0.25
Asian or Asian British Pakistani	0.40	0.36-0.43	0.21	0.20-0.23
Any other Asian background	0.37	0.34-0.40	0.24	0.22-0.27
Any other mixed background	0.45	0.39-0.51	0.22	0.18-0.27
Any other ethnic group	0.39	0.34-0.44	0.20	0.18-0.21
Not stated	0.37	0.34-0.39	0.19	0.17-0.21

Supplementary Table 7: Multivariable logistic regression model displaying the probability of success of application to Specialty Training Posts in the 2021-22 recruitment cycle by Ethnicity and Country of Graduate Status. A logistic regression model is fitted with variation of success at application between ethnic groups by country of qualification measured by including interaction terms between the two variables. Probabilities are presented with 95% Confidence Intervals. Anova analysis of residuals between models where an interaction was included and where it was not, demonstrated a significant difference between the models. Only the ‘Asian or Asian British – Bangladeshi’ group significantly differed in outcome across the graduate status (p=0.02).

Supplementary Figures

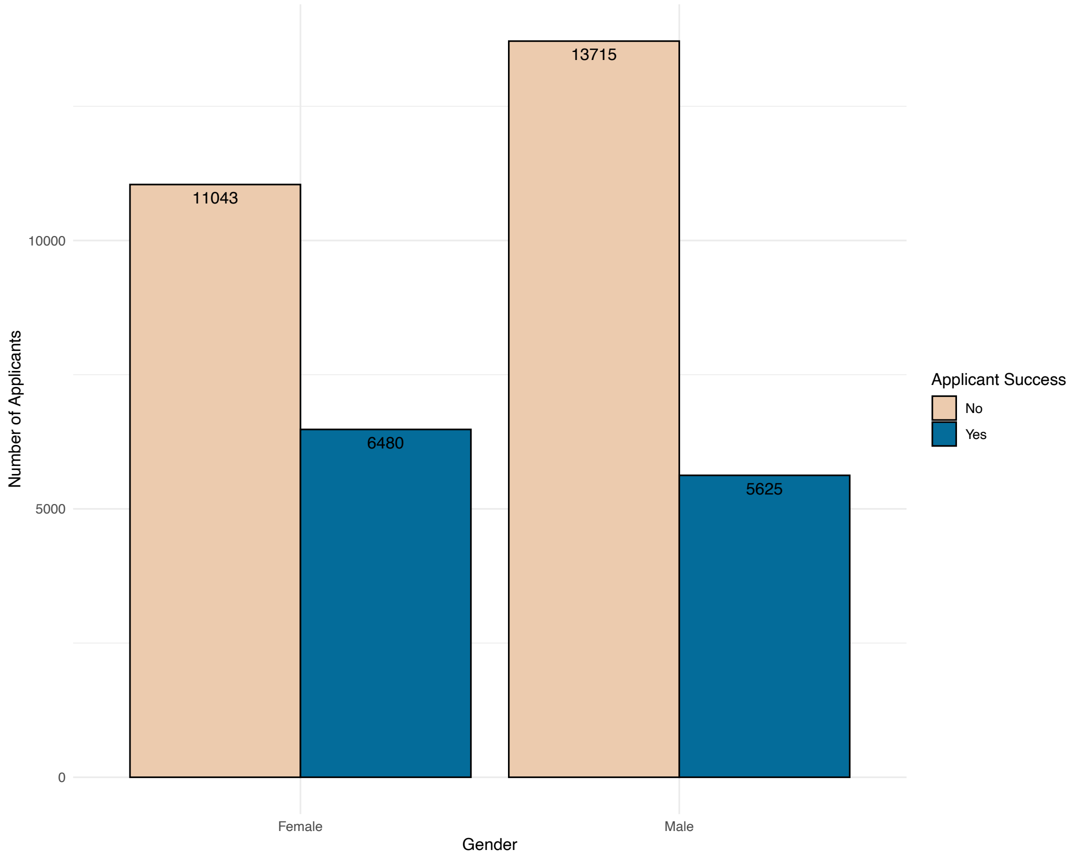

Supplementary Figure 1: Applications and outcomes for Specialty Training Posts in the 2021-22 recruitment year by gender. Bar chart demonstrating applications and outcomes of trainees to Specialty Training by gender. Overall, the difference in percentage of success by females (6480/17523, 37.0%) and males (5625/19340, 29.1%) was 7.9% (95% C.I.= 6.93-8.86%), in favour of females.

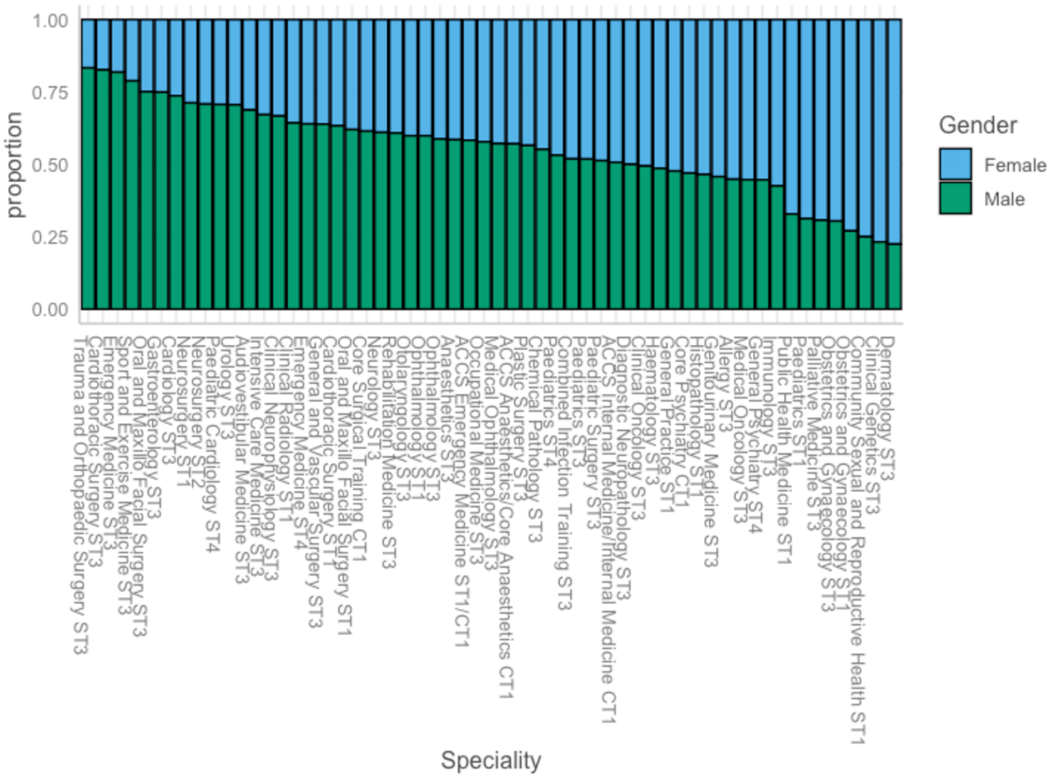

Supplementary Figure 2: Proportion of applicants to individual Specialty Training Posts in the 2021 recruitment year by Gender. Data is presented where complete.

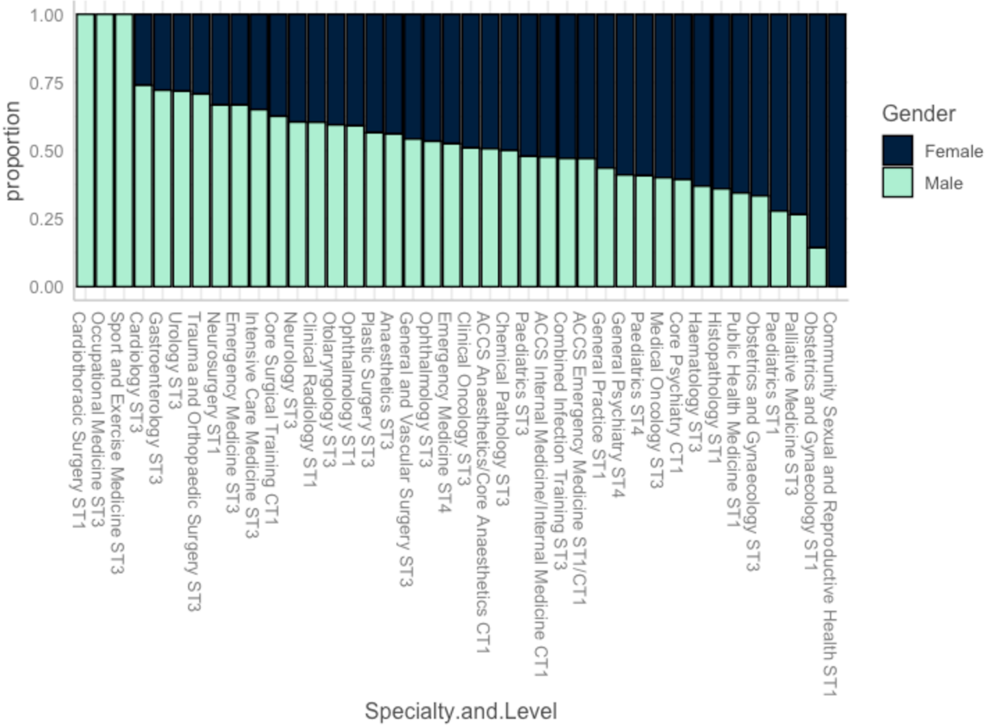

Supplementary Figure 3: Proportion of successful applicants to individual specialities in the 2021 recruitment year by Gender. Data is presented where complete.

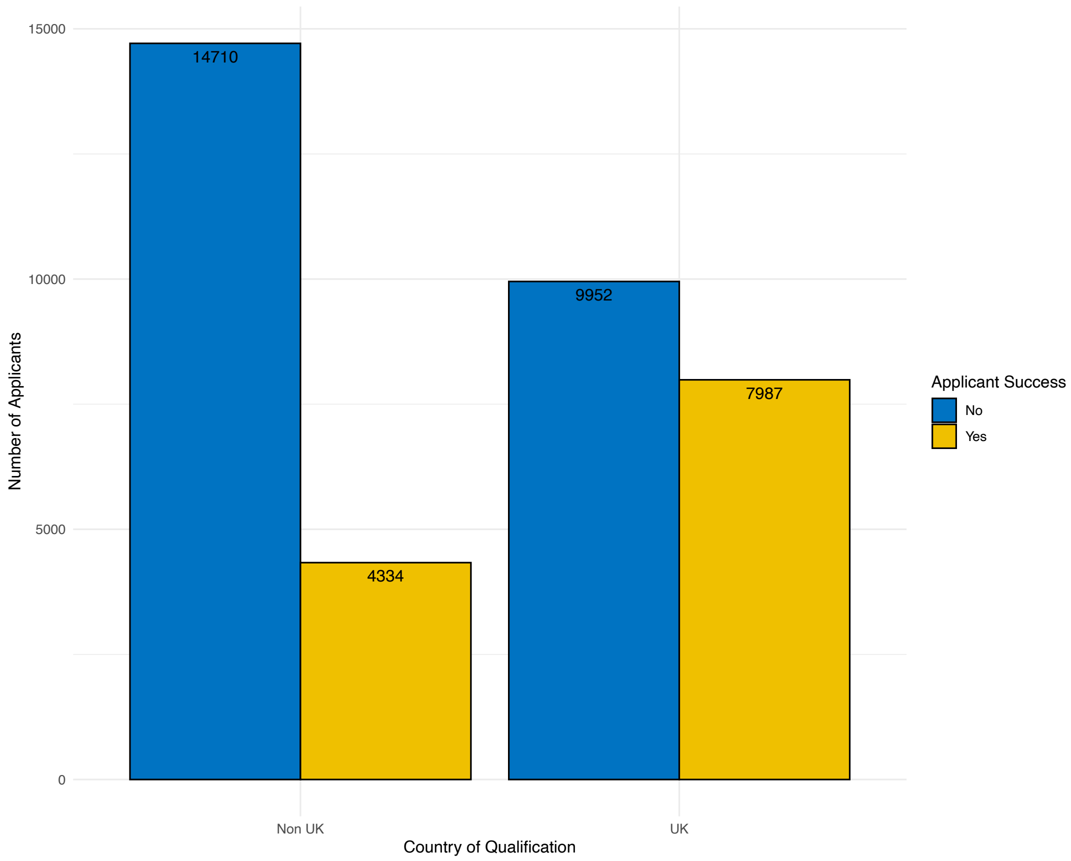

Supplementary Figure 4: Applications and outcomes for Specialty Training Posts in the 2021-2022 recruitment year by ethnicity. (a) Applications to Specialty Training Posts in the 2021 recruitment year by Ethnicity. (b**)** Applications and outcomes by country of qualification (Non-UK vs UK). Overall, the difference in percentage of success by UK graduates (7987/17939, 44.5%) and Non-UK graduates (4334/19044, 22.8%) was 21.8% (95% C.I. = 20.8-22.7%), in favour of UK graduates.

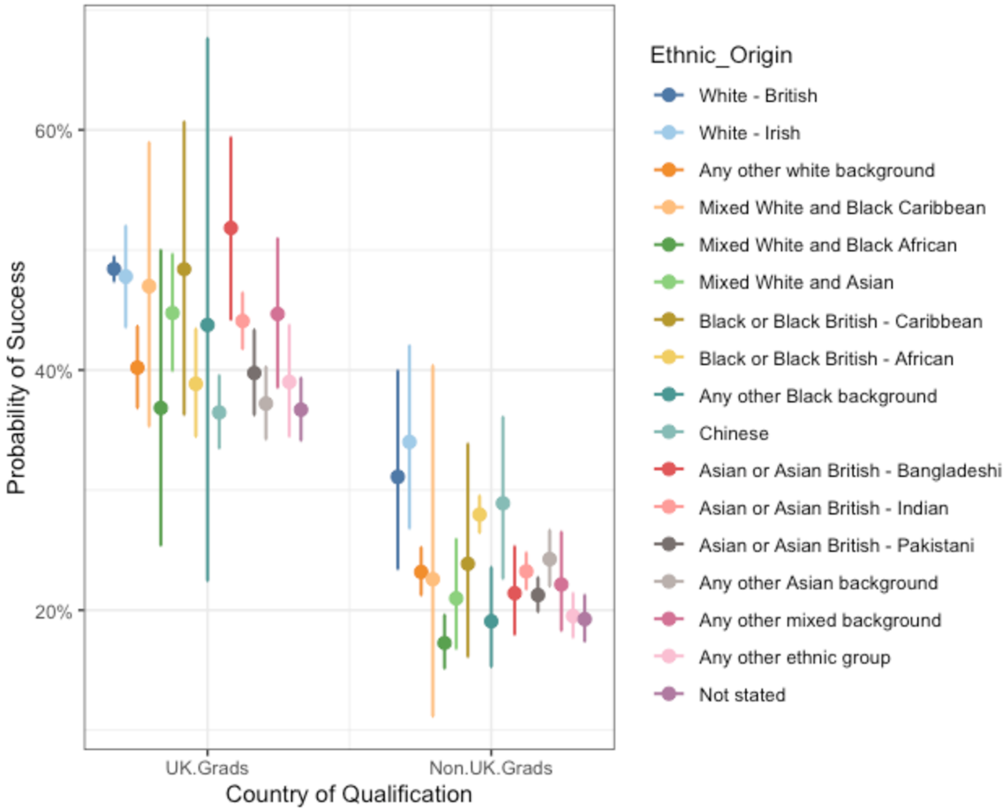

Supplementary Figure 5: Multivariable logistic regression model displaying the probability of success of application to Specialty Training Posts in the 2021-22 recruitment cycle by Ethnicity. A logistic regression model is fitted with variation of success at application between ethnic groups by country of qualification measured by including interaction terms between the two variables. Probabilities are presented with 95% Confidence Intervals. Anova analysis of residuals between models where an interaction was included and where it was not, demonstrated a significant difference between the models. Only the 'Asian or Asian British – Bangladeshi' group significantly differed in outcome across the graduate status.

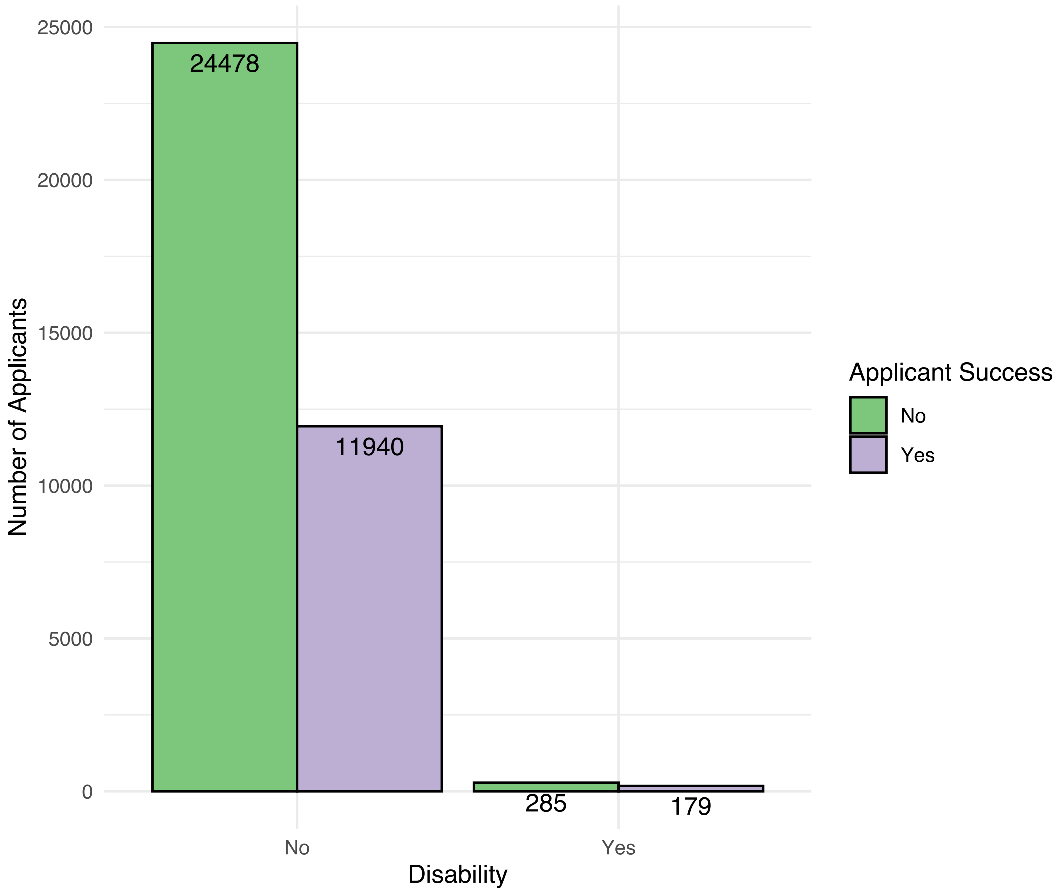

Supplementary Figure 6: **Applications and outcomes to Specialty Training Posts in 2021 by disability status.** Overall, the difference in percentage of success by disabled applicants (179/464, 38.6%) and non-disabled applicants (11940/36418, 32.8%) was 5.79% (95% C.I. = 1.23-10.4%), in favour of disabled applicants.
