# Supplementary material for: Applications to medical and surgical specialist training in the UK National Health Service, 2021–2022: a cross-sectional observational study to characterise the diversity of successful applicants
Source: BMJ Open. 2023 Apr 17;13(4):e069846. doi: 10.1136/bmjopen-2022-069846 (PMC10186087; doi:10.1136/bmjopen-2022-069846)
Supplement: Author's manuscript [file bmjopen-2022-069846.draft_revisions.pdf]

BMJ Open

BMJ Open is committed to open peer review. As part of this commitment we make the peer review history of every article we publish publicly available.

When an article is published we post the peer reviewers' comments and the authors' responses online. We also post the versions of the paper that were used during peer review. These are the versions that the peer review comments apply to.

The versions of the paper that follow are the versions that were submitted during the peer review process. They are not the versions of record or the final published versions. They should not be cited or distributed as the published version of this manuscript.

BMJ Open is an open access journal and the full, final, typeset and author-corrected version of record of the manuscript is available on our site with no access controls, subscription charges or pay-per-view fees (http://bmjopen.bmj.com).

If you have any questions on BMJ Open's open peer review process please email info.bmjopen@bmj.com

BMJ Open

Examining diversity and inclusion in recruitment to medical and surgical specialist training in the UK National Health Service, 2021-22: an observational study

Journal:	*BMJ Open*
Manuscript ID	bmjopen-2022-069846
Article Type:	Original research
Date Submitted by the Author:	08-Nov-2022
Complete List of Authors:	Aggarwal, Dinesh; University of Cambridge, Department of Medicine; Wellcome Sanger Institute<br>Roy-Chowdhury, Meera; Bar Standards Board<br>Xiang, Nicola; Imperial College Healthcare NHS Trust<br>Peacock, Sharon; Univ Cambridge
Keywords:	Health policy < HEALTH SERVICES ADMINISTRATION & MANAGEMENT, Organisational development < HEALTH SERVICES ADMINISTRATION & MANAGEMENT, SURGERY, GENERAL MEDICINE (see Internal Medicine)

SCHOLARONE™
Manuscripts

I, the Submitting Author has the right to grant and does grant on behalf of all authors of the Work (as defined in the below author licence), an exclusive licence and/or a non-exclusive licence for contributions from authors who are: i) UK Crown employees; ii) where BMJ has agreed a CC-BY licence shall apply, and/or iii) in accordance with the terms applicable for US Federal Government officers or employees acting as part of their official duties; on a worldwide, perpetual, irrevocable, royalty-free basis to BMJ Publishing Group Ltd ("BMJ") its licensees and where the relevant Journal is co-owned by BMJ to the co-owners of the Journal, to publish the Work in this journal and any other BMJ products and to exploit all rights, as set out in our licence.

The Submitting Author accepts and understands that any supply made under these terms is made by BMJ to the Submitting Author unless you are acting as an employee on behalf of your employer or a postgraduate student of an affiliated institution which is paying any applicable article publishing charge ("APC") for Open Access articles. Where the Submitting Author wishes to make the Work available on an Open Access basis (and intends to pay the relevant APC), the terms of reuse of such Open Access shall be governed by a Creative Commons licence – details of these licences and which Creative Commons licence will apply to this Work are set out in our licence referred to above.

Other than as permitted in any relevant BMJ Author's Self Archiving Policies, I confirm this Work has not been accepted for publication elsewhere, is not being considered for publication elsewhere and does not duplicate material already published. I confirm all authors consent to publication of this Work and authorise the granting of this licence.

For peer review only

Examining diversity and inclusion in recruitment to medical and surgical specialist training in the UK National Health Service, 2021-22: an observational study

*Dr Dinesh Aggarwal[1,2,3], Ms Meera Roy-Chowdhury[4], Dr Nicola Xiang[5], Prof Sharon J. Peacock[1,2]

Affiliations

1. University of Cambridge, Department of Medicine, Hills Rd, Cambridge, CB2 0QQ
2. Cambridge University Hospital NHS Foundation Trust, Hills Rd, Cambridge, CB2 0QQ
3. Wellcome Sanger Institute, Hinxton, Saffron Walden, CB10 1RQ, UK
4. The Bar Standards Board, High Holborn, London, WC1V 7HZ
5. St Mary's Hospital, Imperial College Healthcare NHS Trust, Praed St, London, W2 1NY

* corresponding authors:

Dr Dinesh Aggarwal, University of Cambridge, Department of Medicine, Cambridge, UK. Email: dinesh.aggarwal@nhs.net. Telephone: +441223 336 849

Declarations of Interest

None

For peer review only

Abstract

Objectives: To compare success of applicants to Specialty Training Posts in the UK by gender, ethnicity, and disability status.

Design: Cross-sectional observational study.

Setting: National Health Service, UK.

Participants: All Specialty Training Post applications to Health Education England, UK, during the 2021 to 2022 recruitment cycle.

Main outcome measures: Comparison of success at application to Specialty Training Posts by gender, ethnicity, country of qualification (UK vs Non-UK), and disability. The influence of ethnicity on success was investigated using a logistic regression model, where country of qualification was included as a covariate.

Results: 12419/37971 (32.7%) of applicants to Specialty Training Posts were successful, representing 58 specialities. The difference in percentage of successful females (6480/17523, 37.0%) and males (5625/19340, 29.1%) was 7.9% (95% C.I=6.93-8.86%), in favour of females. Segregation of applications to specialities by gender was observed; surgical specialities had the highest proportion of male applicants, whilst obstetrics and gynaecology had the highest proportion of female applicants. The proportion of successful recruits to specialities largely reflected the number of applications. 11/15 minority ethnic groups (excluding 'Not stated') had significantly lower adjusted odds-ratios for success compared to White-British applicants. 'Mixed White and Black African' (OR=0.52, 95% C.I=0.44-0.61. *p*=<0.001) were least successful minority group in our study, whilst Non-UK graduates had an adjusted odds-ratio for success of 0.43 (95% C.I=0.41-0.46. *p*=<0.001) compared to UK graduates. The difference in percentage of success by disabled applicants (179/464, 38.6%) and non-disabled applicants (11940/36418, 32.8%) was 5.79% (95% C.I=1.23-10.4%), in favour of disabled applicants. No disabled applicants were accepted to 21/58 (36.2%) of specialities.

Conclusions: Despite greater success by female applicants overall, there is an attraction issue to specialities by gender. Further, most ethnic minority groups are less successful at application when compared to White-British applicants. This requires continuous monitoring and evaluation of the reasons behind observed differences.

Strengths and limitations of this study

- This study utilises a large sample of applicants to a national recruitment portal allowing the estimation of success at application to specialist recruitment posts by demographic groups.

For peer review only

- We evaluate three important protected characteristics - gender, ethnicity, and disability - representing the most comprehensive study of the inclusivity of the national recruitment process, to our knowledge.
- By including country of qualification as a covariate in our analysis we account for a key confounding variable.
- Due to Information Commission Office standards, small specialities are largely excluded from the individual specialty level analysis and intersectional analysis beyond what is presented is not possible.
- Due to limitations on data availability longitudinal effects and those of residual confounding factors, such as the influence of socioeconomic background on the disparate success observed by ethnic minority groups, cannot be ascertained here but are important to study in future work.

Introduction

It is crucial for the National Health Service (NHS) to reflect the society which it serves and to nurture diverse talent effectively. Harnessing diverse lived experiences and perspectives strengthens the pool of knowledge and skills within the profession. Diverse teams are more efficient, innovative and make better decisions, meaning that they are ultimately better placed to serve patients (1). Importantly, a lack of workforce diversity can be detrimental to patient care, with research demonstrating inherent biases influencing how clinicians treat patients (2). The NHS is under-staffed at all levels of seniority; backlogs created by the COVID-19 crisis, systemic under-funding, the increasingly complex care needs of an ageing population, and issues with retention of its medical workforce are only serving to exacerbate this (3). The UK is reported to employ 3.03 doctors per 1000 people compared to an average of 3.7 per 1000 people in the Organisation for Economic Co-operation and Development (OECD) European Union nations (4). These issues are likely to only worsen as the workforce supply and demand gap is predicted to widen (5). Creating a more inclusive culture in the NHS is required

For peer review only - http://bmjopen.bmj.com/site/about/guidelines.xhtml

to ensure that a wider, more diverse talent pool is attracted, able to break into the profession, able to progress successfully, and retained.

Disparate representation within the NHS workforce is well reported (6-8). Surgical specialities have the lowest proportion of female consultants, and gender parity within surgical specialities is predicted to be reached in decades rather than years (8). Such disparities have inevitable knock-on effects. A lack of female representation contributes towards a male dominated culture, and results in fewer female role models to inspire and encourage aspiring female doctors. These are known factors in deterring female applicants as early as undergraduate training (9). A recent independent report examining the gender pay gap in medicine in England found that the mean whole-time equivalent pay gap is 18.9% for hospital doctors, 15.3% for GPs and 11.5% for clinical academics in favour of men; a key recommendation to reduce gender pay was to minimise gender segregation within specialties (6). Additionally, 41.9% of the NHS medical workforce comprises individuals from minority ethnic groups, yet only 22.7% at Clinical Director level (7). Potential bias against minority ethnic groups embedded in recruitment processes and decisions has been demonstrated in multiple sectors and can occur at multiple stages (10-12). A historical study from Esmail et al reported poorer outcomes for matched applicants to specialty medicine with Asian names compared to those with English names (10). More recent data has highlighted differences in applicants deemed appointable for specialty posts by ethnicity, but the data failed to account for the impact of country of graduation – a known additional factor in success at postgraduate examinations and recruitment (13, 14).

As the largest employer in the UK, the NHS must have fair and transparent recruitment processes. This responsibility is reinforced by the fact that the NHS is governed by the Public Sector Equality Duty with the stated objectives to: "eliminate unlawful discrimination; advance equality of opportunity between people who share a protected characteristic and those who don't; foster or encourage good relations between people who share a protected characteristic and those who don't" (15). Systematic examination of disparities in recruitment data by protected characteristics across specialties is therefore a key missing piece of analysis.

For peer review only

Investment of time and resources in investigating any disparities highlighted, addressing the root causes through action and mitigation, and evaluating and measuring progress over time will help to progress diverse representation throughout all levels of seniority, and create a more inclusive culture across the NHS clinical workforce. Here, we examine disparities in the outcomes for the recruitment to all Specialty Training Posts in the UK in 2021 by gender, ethnicity, and disability status.

Methods

Specialty Training Post recruitment process

Obtaining a national training number for specialty training is the route which generates the vast majority of new consultants and general practitioners in the UK. Depending on the specialty, length of training can be variable and entry points for candidates can also vary (i.e. some post-graduate junior doctors may enter specialty training in the first year of the respective Specialty Training Programme or may be deemed to have the competencies required to apply at a later stage in the Programme; these entry points are donated by a prefix of 'ST' or 'CT' followed the year of training at which the Specialty Training Posts is allowing entry. Applications are made online and mostly processed/organised by a lead Health Education England (HEE) local office/devolved nation in order to prevent the need for multiple applications to access posts in different geographical locations. Briefly, the process involves:

- shortlisting of candidates which can include self-assessment or a Multi-Specialty Recruitment Assessment
- an interview, recommended to be multi-panel
- ranking based on shortlisting and interview scores
- offer of appointment.

Demographic details are self-reported. Options for gender include 'Male', 'Female', and 'Not Stated'. Options for ethnicity include 'White - British ', 'Asian or Asian British - Bangladeshi',

For peer review only

‘Asian or Asian British - Indian’, ‘Asian or Asian British - Pakistani’, ‘Chinese’, ‘Any other Asian background’, ‘Black or Black British - African’, ‘Black or Black British - Caribbean’, ‘Any other Black background’, ‘Mixed White and Asian’, ‘Mixed White and Black African’, ‘Mixed White and Black Caribbean’, ‘Any other Mixed background’, ‘White - Irish’, ‘Any other White background’, ‘Any other ethnic background’, and ‘Not stated’. Options for Disability include ‘Yes’ (i.e. applicant has a disability), ‘No’, and ‘Not stated’. Further details on the application process for individual specialties in the UK can be found here, https://specialtytraining.hee.nhs.uk/Recruitment (16).

Study participants

Data on the gender, ethnicity, disability status, and country of qualification (UK vs Non-UK) of applicants to Specialty Training Posts for the 2021-22 recruitment cycle was made available through two separate Freedom of Information (FOI) requests to HEE. HEE follows the Information Commissioner's Office code of practice relating to data anonymisation; release of small numbers (<5 in a geographical area/demographic) has therefore been suppressed. All data made available through the FOI request, including specialities where data is incomplete due to low numbers, can be found in supplementary files (**Supplementary Data**).

Statistical analysis

Analysis was performed in Excel 2016 and R version 4.02. Figures were generated in R version 4.2.1. Probabilities were calculated from estimated models using the emmeans package (1.7.3) (17). Where individual specialities have been grouped for graphical representation, the breakdown of these groups can be found in **Supplementary Table 1**. Differences in percentages of successful recruitment between categories are reported with confidence intervals derived using the prop.test function in R. The influence of ethnicity on success at application to specialty posts is investigated using a logistic regression model by univariable and multivariable analysis (outcome ~ graduate status + ethnic origin) where country of qualification (UK versus Non-UK) is included as a covariate. Variation of success

For peer review only - http://bmjopen.bmj.com/site/about/guidelines.xhtml

at application between ethnic groups by country of qualification is then examined by including interaction terms (outcome ~ graduate status + ethnic origin + graduate status:ethnic origin) between the two variables. This study complies with the STROBE reporting guidelines for observational studies in Epidemiology (18).

<u>Patient and Public Involvement</u>

Patients or the public were not involved in study design, data collection and analysis, decision to publish, or preparation of the manuscript.

Results

There were 12419/37971 (32.7%) successful applicants to Health Education England for Training Posts representing 58 specialty posts in 2021; a distribution of applications by specialty is shown in **Supplementary Table 2**.

<u>*Recruitment by gender*</u>

When considering all applicants, we found 17523/37971 (46.1%) were female. 1108/37971 (2.9%) individuals preferred not to state their gender. Of the successful applicants, 6480/12419 (52.2%) were female and 5625/12419 (45.3%) were male. Overall, the difference in percentage of success by females (6480/17523, 37.0%) and males (5625/19340, 29.1%) was 7.9% (95% C.I.= 6.93-8.86%), in favour of females **(Figure 1, Supplementary Figure 1**).

Recruitment to Specialities by gender

56/58 Specialty Training Posts had complete data representing male and female applications and 40/58 Specialty Training Posts had complete data representing successful male and female applications (excluding applicants with undisclosed gender). When considering applications by individual specialty, 13/40 specialty posts had a higher percentage of successful females when compared to males, compared to specialty 2/40 posts that had a

For peer review only

higher percentage of successful males (**Figure 1**). Despite this, 22/40 specialty posts had a greater absolute number of successful male applicants compared to females, representing an 'attraction' issue. When examining absolute numbers of applicants and successful applicants by gender, there was an observed segregation by specialty (**Figure 2, Supplementary Figures 2 and 3**). Of note, surgical specialities (3097/4742, 65.3%) and radiology (1146/1783, 64.3%) had the highest proportion of male applicants, whilst obstetrics and gynaecology (957/1321, 72.4%) and public health (644/959, 67.2%) had the highest proportion of female applicants. The proportion of successful recruits to specialities largely reflected the number of applications (**Figure 2c**).

Recruitment by Ethnicity and Country of Qualification

Applications for Specialty Training Posts by ethnicity are shown in **Figure 3**. 19044/37971 (50.2%) of applicants to Specialty Training Posts were Non-UK medical graduates in the 2021-22 application cycle **(Supplementary Figure 4)**. Excluding non-medical applicants to Public Health Training Posts, of the successful applicants, 4334/12419 (34.9%) were Non-UK graduates and 7987/12419 (64.3%) were UK graduates. Overall, the difference in percentage of success by UK graduates (7987/17939, 44.5%) and Non-UK graduates (4334/19044, 22.8%) was 21.8% (95% C.I. = 20.8-22.7%), in favour of UK graduates (**Supplementary Table 4**). UK graduates were more successful in all ethnic groups other than 'Chinese' and 'Any other Black background' (**Supplementary Table 4**).

Using a multivariable logistic regression analysis, including country of qualification as a covariate, we found 11/15 minority ethnic groups (excluding 'Not stated') to have a significantly lower odds ratio of success at recruitment when compared to White-British applicants (**Figure 4b and Supplementary Table 5**). Non-UK graduates had a significantly lower odds ratio of success at recruitment when compared to UK-graduates (adjusted OR=0.43, 95% C.I = 0.41 to 0.46. p = <0.001). All adjusted and unadjusted odd-ratios for success by country of qualification and minority ethnic group are provided in **Supplementary Tables 5** and **6**.

For peer review only - http://bmjopen.bmj.com/site/about/guidelines.xhtml

Additionally, only the 'Asian or Asian British – Bangladeshi' group significantly differed in outcome across the graduate status (p=0.02) in the interaction model. (**Supplementary Table 7 and Supplementary Figure 5**).

Recruitment by Disability

When considering all applicants to Specialty Training Posts, we found 464/37971 (1.2%) were disabled. 1089/37971 (2.9%) individuals preferred not to state their disability status. Of the successful applicants, 179/12419 (1.4%) were disabled and 11940/12419 (96.1%) were non-disabled. Overall, the difference in percentage of success by disabled applicants (179/464, 38.6%) and non-disabled applicants (11940/36418, 32.8%) was 5.79% (95% C.I. = 1.23-10.4%), in favour of disabled applicants (**Supplementary Figure 6**). However, there were no disabled applicants to 13/58 specialities and a further 21/58 specialities where no disabled applicants were accepted. Of the specialities where data was available, General Psychiatry ST4 had the highest acceptance rate for disabled applicants (15/16, 93.8%), whilst General Practice had the highest absolute number of successful applicants (61/121, 50.4%).

Discussion

We present the most complete assessment of diversity data relating to recruitment to Specialty Training Posts in the UK to our knowledge. Overall, of the specialties studied, female applicants were more successful in the recruitment process though we note clear segregation of applications by specialty. We observe a low absolute number of successful female applicants to Surgical specialities, Radiology, and Ophthalmology, and a low absolute number of successful male applicants to Obstetrics and Gynaecology, Public Health and Paediatric Medicine. We show that several minority ethnic groups are less likely to be successful in their applications when adjusting for country of graduation. Finally, we find disabled applicants to be more successful in the recruitment process when compared to non-disabled applicants, but again with significant variation in applications and success at individual specialty level. The NHS has committed to fostering a truly inclusive environment; this work, and the

For peer review only - http://bmjopen.bmj.com/site/about/guidelines.xhtml

associated actions (**Box 1**), should serve as a framework to evaluate recruitment data, and subsequently review policies and processes to enable the implementation of improvements, to ensure that this commitment can be achieved.

Strengths and limitations of this study, and future work

We use a large sample of applicants to a national recruitment portal maintained by HEE which allows us to estimate the success at application to specialist recruitment posts by demographic groups. We evaluate three important protected characteristics, gender, ethnicity, and disability, which represents the most comprehensive evaluation of the inclusivity of the national recruitment process to our knowledge. By including country of qualification as a covariate in our analysis, we account for a key confounding variable, demonstrating unequal success by ethnicity despite country of qualification; an important result that suggests the recruitment process needs further investigation.

This study has a number of limitations. Due to Information Commission Office standards, small specialities are largely excluded from the individual specialty level analysis. Due to the nature of the data, multivariable analysis beyond what is presented is not possible; an intersectional analysis of protected characteristics, e.g. females from ethnic minority backgrounds, should be conducted to identify groups that may be particularly disadvantaged. Further, residual confounding factors, such as the influence of socioeconomic background on the disparate success observed by ethnic minority groups cannot be ascertained here but present an important factor to study in future work. The data is representative of one year of recruitment, and possible enduring effects of the COVID-19 pandemic on applications and recruitment patterns cannot be disregarded; ongoing systematic data monitoring is necessary to evaluate trends in applications and successful recruitment by demographic characteristics over time. The data was obtained through two separate FOI requests; one representing aggregated data for 'all Specialty Training Posts' and the second including data separated by individual specialties. Data analysis does not include comparison of the aggregated data between

For peer review only - http://bmjopen.bmj.com/site/about/guidelines.xhtml

datasets but we do note a minor difference in the total number of applicants between datasets (raw data is provided in supplementary files) – given the large sample size, these are not expected to influence our findings. We report an analysis of recruitment to Specialty Training Posts which is only one aspect of assessing the diversity and inclusiveness of a profession; additional work should assess how these findings relate to pay gaps, progression and retention of the workforce, and for any barriers faced by groups representing other protected characteristics as defined by the Equality Act 2010 within the NHS. We note 'Black or Black British – African', 'Black or Black British – Caribbean', and 'Mixed White and Black Caribbean' applicants do not have a lower odds ratio of success at application when compared to White British applicants; this does not mean the absolute numbers of applicants are not disproportionately lower or that this group are not disadvantaged at other stages of their career progression. Finally, to reiterate, the NHS workforce should reflect the population it serves at all levels. This extends beyond clinicians – similar work should be undertaken for allied health professionals and support staff in the NHS.

Implications and supporting evidence

Over-representation of males in senior positions of medicine in the NHS, and in particular specialities such as surgery, has been noted previously (19) but national data on recruitment across specialty posts is limited. Success by female applicants in many specialties, demonstrated in our study, is a positive step towards gender balance, and perhaps reflects existing efforts to address disparities, or higher applicant quality where female applications are low. However, the skew in applications and subsequent recruitment by gender, particularly amongst surgical specialities, is concerning. There is a clear attraction issue (i.e. a lack of diversity amongst applicants) amongst multiple specialities (**Figure 2a and Supplementary Figure 1**), with several possible reasons. Taking surgery as an example, factors including a male-dominated workplace culture, incidents of bullying and harassment, few female role models, and career inflexibility, have been suggested as the reasons why females are deterred from considering to apply, as early as undergraduate study (9, 20). Female surgeons

For peer review only - http://bmjopen.bmj.com/site/about/guidelines.xhtml

have reported quality of life and fewer unsocial hours as explanations of why women prefer other clinical specialities, in addition to the fear that working less-than-full-time (LTFT) or taking career breaks is perceived negatively (20). There are exceptions to this where some specialities appear to have succeeded in creating a culture that attracts female trainees and allows them to thrive. Obstetrics and Gynaecology provides a clear example of a demanding specialty requiring shift work and a surgical skill set (21) where females dominate applications and successful hires. Obstetrics and Gynaecology, Public Health, and Paediatric medicine, are known to have a higher representation of females compared to males (6), and continue to disproportionately recruit females; these represent 3 or the 4 specialities with the highest proportion of LTFT trainees (22). The availability of LTFT opportunities compound gender segregation within specialities, and can exacerbate the gender pay gap (6). Additional specialty-specific factors will inevitably influence a doctor's application decision-making, including personal choice and even patient preference (studies have demonstrated a slight preference for female Obstetric and Gynaecology clinicians by their female patient cohort, for example (23)), and these should be taken into consideration. However, the aim should be to ensure that doctors are given the opportunity to flourish in all specialities and that this is facilitated by fair recruitment.

Concerningly, we find disparities in the success of applicants by ethnicity and this is not explained by country of graduation alone. A previous descriptive analysis demonstrated that doctors from minority ethnic groups were less likely to be considered suitable for appointment to Specialty Training Posts in the UK, but did not consider country of graduation or undertake a thorough analysis of the data (24). Examining Combined Infection Training specifically, country of qualification possibly accounted for imbalances in recruitment by ethnicity (13). Our findings, however, indicate a need to thoroughly review recruitment policies and processes from a diversity and inclusion perspective (such as bias in recruitment decisions and a lack of inclusivity in the design of recruitment processes), to ensure that they are facilitating equitable outcomes. Outside of recruitment to specialty training, unequal progression by ethnicity has

For peer review only

been demonstrated in the NHS. Doctors from minority ethnic groups are underrepresented at consultant grade and within academic roles (7). They have reported disproportionately high levels of discrimination from work colleagues, and had poorer outcomes at revalidation and Annual Review of Competence Progression, regardless of country of qualification. Additionally, differences in postgraduate examination pass rates by country of qualification, and between minority ethnic groups are established (14). These differences can be overcome; the NHS WRES demonstrated that in regions where a concerted effort to improve disparate outcomes for ethnic minority employees had been made, significant improvements were seen (25). We should be mindful that unequal outcomes may reflect a history of disparities in the opportunities available to, and experiences of, various demographic groups and we encourage the NHS to support access schemes that aim to help doctors at an early-career stage. The 'widening access' initiative introduced by HEE, for example, is a positive step in attempting to support the applications of Non-UK graduate doctors to Specialty Training Posts (26); schemes such as these should be maintained after HEE merges with NHS England. The fragmented nature of the NHS and its complex employment structures mean monitoring progression and attainment by protected characteristics is perhaps more complex than other professions, but effort must be made to formulate a consistent methodology to do so. The NHS is facing a workforce crisis (3) and doctors from ethnic minority backgrounds are being increasingly relied on in the UK. They comprise 41.9% (53,157) of the medical and dental workforce in NHS trusts and clinical commissioning groups in England in 2020 (7); therefore, ensuring that these doctors are able to work within an inclusive environment, that allows them to thrive and progress should be a priority.

Only 1.2% of the applicants to Specialty Training Posts reported a disability – this will likely be an under-estimate with under-reporting of disabilities amongst medical professionals (27). It is encouraging, however, to observe a high proportion of acceptances amongst all individuals disclosing a disability, with a particularly high proportion of success seen in Psychiatry and Paediatric Medicine. The 2021 Workforce Disability Equality Standard found 23.4% of the

For peer review only

disabled staff working in the NHS did not have the adjustments necessary to effectively work in the NHS (27). Ultimately, the NHS needs to ensure that application and recruitment processes are accessible and open to adjustments for all disabilities (including neurodiversity), eliminate any perceived fear of discrimination, and provide assurance that all NHS workplaces will accommodate reasonable adjustments to ensure that disabled doctors can carry out their work. This will not only help to encourage more disabled applicants, but also make disabled clinicians to feel more comfortable disclosing this information, in turn support the monitoring and evaluation of diversity data. Additionally, given the diversity of issues faced by disabled doctors, we encourage assessment of this protected characteristic by taking the nature of one's disability into account. Stakeholders should engage with disabled doctors through networks such as the Disability Doctors Network, to identify possible barriers to disclosing their disability, applying to become, and working as a doctor, and how these could be overcome. Disabled doctors harbour unique insights and provide an opportunity to relate to the patient journey (28); the NHS would stand to benefit from this expertise if they are supported to prosper within its environment.

Conclusions

Overall, we present detailed analysis of the diversity and inclusion considerations for recruitment to specialty training in the UK for the 2021-22 cycle. This study provides evidence to demonstrate higher success rates by females compared to men but gender segregation of applications by specialty. Our data highlights poorer outcomes for minority ethnic applicants, regardless of country of graduation. We show favourable outcomes for disabled doctors and encourage data reporting in this domain to be improved. Examination of recruitment processes is the first step towards building an inclusive workplace; we encourage policy makers to investigate the root cause of our findings and ensure such monitoring is made routine.

For peer review only - http://bmjopen.bmj.com/site/about/guidelines.xhtml

Recruitment specific recommendation
Processes and data pertaining to the allocation of Specialty Training Posts should be transparent and published.
Ensure that recruitment panels are as diverse as possible.
Ensure that all staff involved in recruitment processes are trained in fair and inclusive recruitment, with regular refresher training.
Language used in applications should be inclusive and examined to ensure it does not deter any demographic group.
Review job criteria to ensure that only "essential" skills and experience is included. Women for example, are less likely to apply for a job where they do not meet all the criteria.
Include a self-identification category for gender.
Recruitment systems and processes should be flexible to accommodate for reasonable adjustments. This should be plainly messaged to applicants, along with a clearly signposted process for requesting reasonable adjustments.
Review application tests for inclusivity and fairness across protected characteristics.
Implement positive action schemes to support applicants from groups with disproportionately low success rates.
Wider approaches to promote inclusivity to address attraction issues
Support all genders with less-than-full-time applications.
Actively support woman returning from maternity leave to increase confidence and mitigate de-skilling (such as return to work transition programmes and 'keeping-in-touch' days).
De-stigmatise parental leave for all genders.
Encourage male applications to traditionally female dominated specialities to help break-down existing gender stereotypes within the NHS.
Promote diversity in specialities that are under-represented by certain demographic groups by highlighting role models and positive messaging.
Investigate cultural issues within specialties where this is a problem; carry out engagement surveys and focus groups to determine how these can be addressed.
Create and promote a culture with zero tolerance for bullying and harassment, and ensure that doctors feel safe to speak out.
Create outreach programmes for those specialties with attraction issues for certain groups.
Ensure workplaces are accessible and processes for reasonable adjustments (including successful examples of reasonable adjustments) are well advertised.
Engage with employee networks and patient groups to understand specific issues faced by different demographic groups and promote Equality, Diversity, and Inclusion related activities.

Box 1: Actions for consideration to improve inclusivity in the Specialty Post Recruitment process

Acknowledgements

We thank members of the Freedom of Information office at Health Education England for providing data. We thank Mr Vivek Roy Chowdhury, Department of Economics, University of Cambridge, and Dr Oliver Feng, Department of Mathematics, University of Cambridge, for providing direction on statistical analysis.

Ethics approval

This study uses data obtained from a freedom of information request, that is routinely collected and obtained in line with the Information Commissioner's Office. Ethical approval is therefore not required.

Transparency statement

Author DA (manuscript guarantor) affirms the manuscript is an honest, accurate, and transparent account of the study being reported; that no important aspects of the study have been omitted; and that any discrepancies from the study as originally planned (and, if relevant, registered) have been explained.

Competing interests statement

All authors have completed the Unified Competing Interest form (available on request from the corresponding author) and declare: no support from any organisation for the submitted work; no financial relationships with any organisations that might have an interest in the submitted work in the previous three years, no other relationships or activities that could appear to have influenced the submitted work.

Data statement

The datasets analysed during this study are made freely available in the supplementary materials.

Contributions

Conceptualisation: D.A, M.R.C; Project administration: D.A.; Investigation: D.A.; Data curation: D.A.; Methodology: D.A., M.R.C.; Visualization: D.A.; Formal analysis: D.A.;

For peer review only

Writing—original draft preparation: D.A.; Writing—review and editing: D.A, M.R.C, N.X., S.J.P.; Funding acquisition: N/A; Supervision: S.J.P.

Copyright Statement

The corresponding author has the right to grant on behalf of all authors and does grant on behalf of all authors, an exclusive licence on a worldwide basis to the BMJ Publishing Group Ltd to permit this article (if accepted) to be published in BMJ editions and any other BMJPGL products and sublicences such use and exploit all subsidiary rights, as set out in our licence.

Funding Statement

DA is a Clinical PhD Fellow and gratefully supported by the Wellcome Trust (Grant number: 222903/Z/21/Z). This research did not receive any specific grant from funding agencies in the public, commercial, or not-for-profit sectors. This is an open access article distributed in accordance with the terms of the Creative Commons Attribution (CC BY 4.0) license, which permits others to distribute, remix, adapt and build upon this work, for commercial use, provided the original work is properly cited. See: http://creativecommons.org/licenses/by/4.0.

References

1. Gomez LE, Bernet P. Diversity improves performance and outcomes. J Natl Med Assoc. 2019;111(4):383-92.
2. Rotenstein LS, Reede JY, Jena AB. Addressing Workforce Diversity - A Quality-Improvement Framework. N Engl J Med. 2021;384(12):1083-6.
3. Committee HaSC. Workforce: recruitment, training and retention in health and social care: UK Parliament; 2022 [Available from: https://publications.parliament.uk/pa/cm5803/cmselect/cmhealth/115/report.html.
4. Association BM. NHS medical staffing data analysis 2022 [Available from: https://www.bma.org.uk/advice-and-support/nhs-delivery-and-workforce/workforce/nhs-medical-staffing-data-analysis.
5. Shembavnekar N BJ, Bazeer N, Kelly E, Beech J, Charlesworth A, McConkey R, Fisher R. . NHS workforce projections 2022. The Health Foundation. 2022.
6. Jane Dacre CW, Carol Atkinson, Ioannis Laliotis, Mark Williams, Jo Blanden, Sheila Wild, Duncan Brown. Mend the Gap: The Independent Review into Gender Pay Gaps in Medicine in England: Department of Health and Social Care 2020; 2020 [Available from: https://assets.publishing.service.gov.uk/government/uploads/system/uploads/attachment_data/file/944246/Gender_pay_gap_in_medicine_review.pdf.
7. team WI. Medical Workforce Race Equality Standard (MWRES): WRES indicators for the medical workforce 2020: NHS England; 2021 [Available from:

For peer review only

https://www.england.nhs.uk/wp-content/uploads/2021/07/MWRES-DIGITAL-2020_FINAL.pdf.

8. Newman TH, Parry MG, Zakeri R, Pegna V, Nagle A, Bhatti F, et al. Gender diversity in UK surgical specialties: a national observational study. BMJ Open. 2022;12(2):e055516.

9. Skinner H, Bhatti F. Women in surgery. The Bulletin of the Royal College of Surgeons of England. 2019;101(suppl_6):12-4.

10. Esmail A, Everington S. Racial discrimination against doctors from ethnic minorities. Bmj. 1993;306(6879):691-2.

11. Wood M, Hales J, Purdon S, Sejersen T, Hayllar O. A test for racial discrimination in recruitment practice in British cities. Department for Work and Pensions Research Report. 2009;607.

12. Adamovic M. When ethnic discrimination in recruitment is likely to occur and how to reduce it: Applying a contingency perspective to review resume studies. Hum Resour Manage R. 2022;32(2).

13. Aggarwal D, Roy-Chowdhury M, Xiang N, Peacock SJ. Equity in recruitment to Combined Infection Training, 2021: Diversity & Inclusion considerations. J Infect. 2022.

14. MRCP(UK) and Specialty Certificate Examinations Pass Rates by Gender and Ethnicity 2021: MRCP(UK); 2021 [Available from: https://www.mrcpuk.org/sites/default/files/documents/Equality%20report%202021.pdf.

15. office Ge. Equality Act 2010: Public Sector Equality Duty. What do I need to know? A quick start guide for public sector organisations.: GOV.UK; 2011 [Available from: https://assets.publishing.service.gov.uk/government/uploads/system/uploads/attachment_data/file/85041/equality-duty.pdf.

16. England HE. Medical Specialty Recruitment 2022: Health Education England; [Available from: https://specialtytraining.hee.nhs.uk/Recruitment.

17. Lenth R, Singmann H, Love J, Buerkner P, Herve M. Emmeans: Estimated marginal means, aka least-squares means. R package version. 2018;1(1):3.

18. Vandenbroucke JP, von Elm E, Altman DG, Gotzsche PC, Mulrow CD, Pocock SJ, et al. Strengthening the Reporting of Observational Studies in Epidemiology (STROBE): explanation and elaboration. Int J Surg. 2014;12(12):1500-24.

19. Digital N. Analysis of the representation of women across the hospital and community health services workforce: NHS Digital; 2018 [Available from: https://digital.nhs.uk/data-and-information/find-data-and-publications/supplementary-information/2018-supplementary-information-files/analysis-of-the-representation-of-women-across-the-hospital-and-community-health-services-workforce.

20. Bellini MI, Graham Y, Hayes C, Zakeri R, Parks R, Papalois V. A woman's place is in theatre: women's perceptions and experiences of working in surgery from the Association of Surgeons of Great Britain and Ireland women in surgery working group. BMJ Open. 2019;9(1):e024349.

21. Wise J. Life as a physician in obstetrics and gynaecology. BMJ. 2020;368:m37.

22. Council GM. National training survey: General Medical Council; 2022 [Available from: https://www.gmc-uk.org/education/how-we-quality-assure-medical-education-and-training/evidence-data-and-intelligence/national-training-surveys.

23. Janssen SM, Lagro-Janssen AL. Physician's gender, communication style, patient preferences and patient satisfaction in gynecology and obstetrics: a systematic review. Patient Educ Couns. 2012;89(2):221-6.

24. Iacobucci G. Specialty training: ethnic minority doctors' reduced chance of being appointed is "unacceptable". BMJ. 2020;368:m479.

For peer review only

25. England N. NHS Workforce Race Equality Standard: 2021 data analysis report for NHS trusts: NHS England; 2022 [Available from: https://www.england.nhs.uk/wp-content/uploads/2022/04/Workforce-Race-Equality-Standard-report-2021-.pdf.
26. England HE. Widening Access to Specialty Training: start your medical career in the NHS hee.nhs.uk: Health Education England; [Available from: https://specialtytraining.hee.nhs.uk/wideningaccess.
27. Workforce Disability Equality Standard: 2021 data analysis report for NHS trusts and foundation trusts: NHS England; 2022 [Available from: https://www.england.nhs.uk/wp-content/uploads/2022/05/Workforce-Disability-Equality-Standard-2021-data-analysis-report-for-NHS-trusts-and-foundation-trusts.pdf.
28. GMC. Welcomed and valued: Supporting disabled learners in medical education and training GMC: GMC; 2019 [Available from: https://www.gmc-uk.org/education/standards-guidance-and-curricula/guidance/welcomed-and-valued.

Figure Legends

Figure 1: Success of applicants to Training Posts by specialty in the 2021 recruitment year by gender.
Figure showing the applications of trainees to Specialty Training Posts and percentage of successful applicants by gender, where data is complete. All values are provided to 3 significant figures. Where confidence intervals for estimated differences between the percentage of successful applicants by gender does not cross zero, bars are highlighted in orange (greater female success) or purple (greater male success). Specialities marked with ^ contained small numbers (i.e expected frequencies were lower than 5 in any domain of the contingency table) and therefore Fisher-exact test results to examine differences in proportions of successful candidates are also provided in **Supplementary Table 3**.

Figure 2: Applications and outcomes for Specialty Training Posts separated into groups of specialities in the 2021-22 recruitment year by gender.
(a) Proportion of applicants to specialty groups by gender. (b) Number of applicants to specialty groups by gender. (c) Proportion of successful applicants to specialty groups by gender. (d) Number of successful applicants to specialty groups by gender. Groupings of specialties can be found in **Supplementary Table 1**.

Figure 3: Applications and outcomes for Specialty Training Posts in the 2021-2022 recruitment year by ethnicity.
Applications to Specialty Training Posts in the 2021 recruitment year by Ethnicity.

Figure 4: Applications and outcomes for Specialty Training Posts in the 2021-2022 recruitment year by ethnicity and graduate status.
(a) Unadjusted Odds ratios for success by ethnicity when compared to White British applicants derived from a logistic regression model (b) Adjusted Odds ratios for success by ethnicity compared to White-British where country of qualification (UK versus Non-UK) is included as a covariate derived from a multivariable logistic regression model. Non-Medical applicants to Public Health Specialty Training have been removed (n=988). Odds ratios are presented with 95% Confidence Intervals. * *p<0.05* ** *p<0.01* *** *p<0.001*.

For peer review only - http://bmjopen.bmj.com/site/about/guidelines.xhtml

Specialty	Total	Male Success (%)	Female Success (%)	Percentage Difference (95% CI)
General Practice ST1	9803	41.80	49.40	7.62 (5.63 to 9.61)
Core Surgical Training CT1	2418	22.30	21.40	−0.99 (−4.45 to 2.47)
ACCS Internal Medicine/Internal Medicine CT1	3495	41.10	46.50	5.41 (2.06 to 8.75)
ACCS Anaesthetics/Core Anaesthetics CT1	2390	23.70	30.70	7.02 (3.32 to 10.70)
Clinical Radiology ST1	1783	19.40	22.90	3.55 (−0.56 to 7.66)
Core Psychiatry CT1	2056	26.80	36.40	9.68 (5.59 to 13.80)
ACCS Emergency Medicine ST1/CT1	1382	20.50	32.30	11.80 (6.94 to 16.70)
Anaesthetics ST3	1590	33.90	37.40	3.55 (−1.36 to 8.47)
Trauma and Orthopaedic Surgery ST3	587	25.20	52.00	26.90 (15.70 to 38.10)
General and Vascular Surgery ST3	595	18.70	27.90	9.22 (1.70 to 16.70)
Ophthalmology ST1	593	13.80	14.30	0.48 (−5.58 to 6.55)
Public Health Medicine ST1	959	10.50	9.78	−0.69 (−5.02 to 3.63)
Intensive Care Medicine ST3	584	32.70	35.90	3.28 (−5.33 to 11.90)
Cardiology ST3	386	23.90	23.50	−0.41 (−10.40 to 9.61)
Obstetrics and Gynaecology ST1	852	15.70	34.70	19.10 (12.80 to 25.40)
Emergency Medicine ST3^	231	6.35	14.30	7.94 (−4.66 to 20.50)
Gastroenterology ST3	250	16.60	19.00	2.47 (−9.66 to 14.60)
Paediatrics ST1	870	42.60	50.50	7.85 (0.47 to 15.20)
Neurosurgery ST1^	232	6.06	7.46	1.40 (−6.92 to 9.72)
Haematology ST3	358	17.80	28.80	11.00 (1.76 to 20.20)
Histopathology ST1	336	23.70	36.70	12.90 (2.65 to 23.20)
Combined Infection Training ST3	266	23.20	28.10	4.94 (−6.32 to 16.20)
General Psychiatry ST4	637	62.70	72.50	9.85 (2.22 to 17.50)
Paediatrics ST3	220	9.65	11.30	1.67 (−7.35 to 10.70)
Urology ST3	193	24.30	22.80	−1.46 (−15.80 to 12.80)
Obstetrics and Gynaecology ST3	309	7.45	6.51	−0.94 (−7.95 to 6.08)
Paediatrics ST4	175	11.80	19.50	7.68 (−4.26 to 19.60)
Plastic Surgery ST3	200	34.50	34.50	−0.03 (−13.30 to 13.30)
Clinical Oncology ST3	257	43.30	40.80	−2.54 (−15.40 to 10.30)
Otolaryngology ST3	152	20.90	21.30	0.43 (−13.20 to 14.10)
Neurology ST3	159	29.90	30.60	0.75 (−14.70 to 16.10)
Cardiothoracic Surgery ST1^	109	8.70	0.00	−8.70 (−17.30 to −0.07)
Medical Oncology ST3	204	33.00	39.80	6.86 (−7.36 to 21.10)
Emergency Medicine ST4	136	36.80	59.20	22.40 (3.72 to 41.10)
Community Sexual and Reproductive Health ST1^	160	0.00	5.00	5.00 (−0.57 to 10.60)
Ophthalmology ST3	80	17.00	21.20	4.19 (−16.00 to 24.40)
Palliative Medicine ST3	101	29.00	35.70	6.68 (−15.20 to 28.50)
Occupational Medicine ST3^	45	23.10	0.00	−23.10 (−43.80 to −2.33)
Sport and Exercise Medicine ST3^	33	23.10	0.00	−23.10 (−48.30 to 2.18)
Chemical Pathology ST3^	29	31.20	38.50	7.21 (−34.60 to 49.00)
All Applicants*	36863	29.10	37.00	7.90 (6.93 to 8.86)

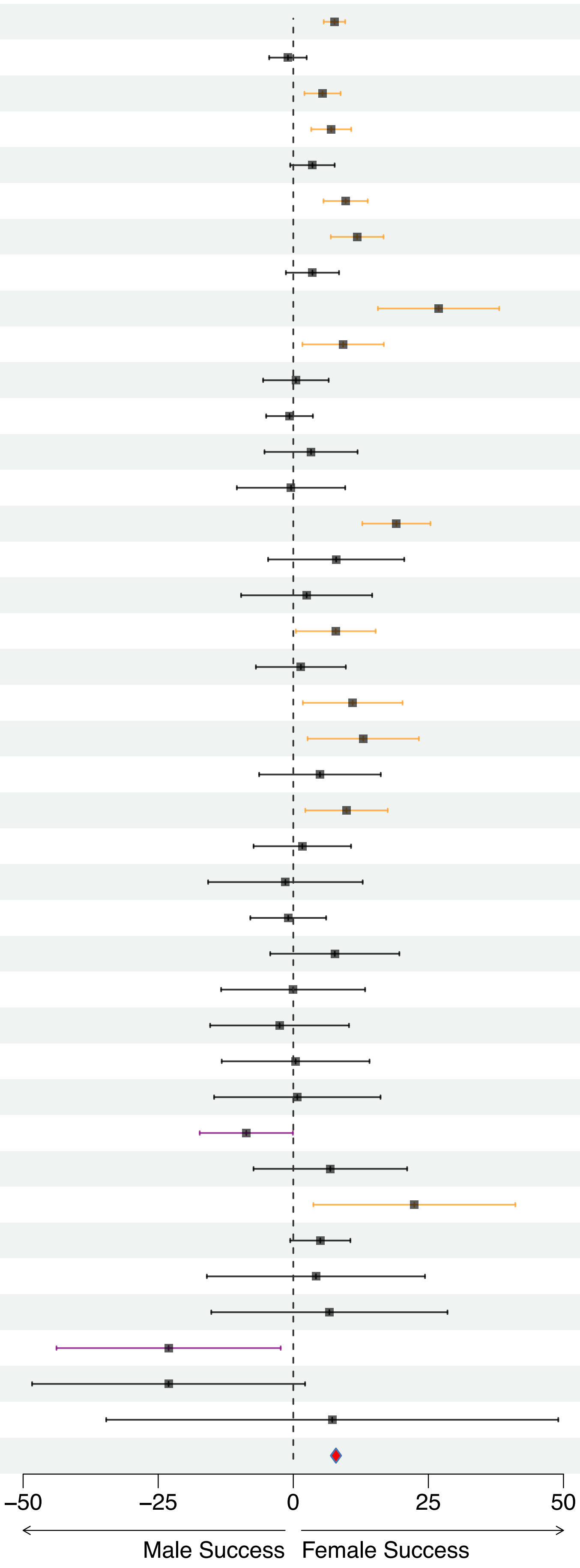

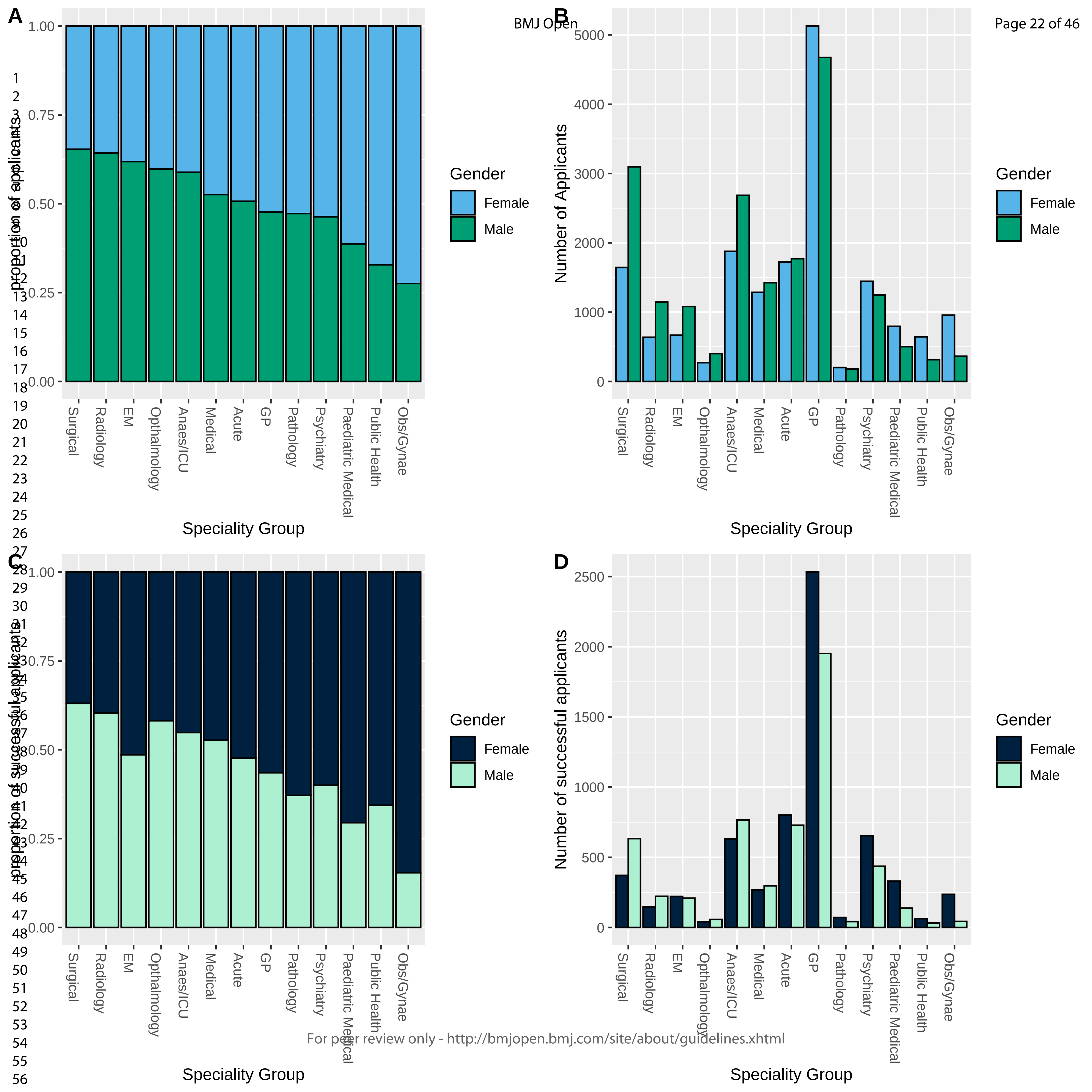
A
proportion of applicants
Gender
Female
Male
Speciality Group
Surgical
Radiology
EM
Opthalmology
Anaes/ICU
Medical
Acute
GP
Pathology
Psychiatry
Paediatric Medical
Public Health
Obs/Gynae
B
Number of Applicants
C
proportion of successful applicants
D
Number of successful applicants

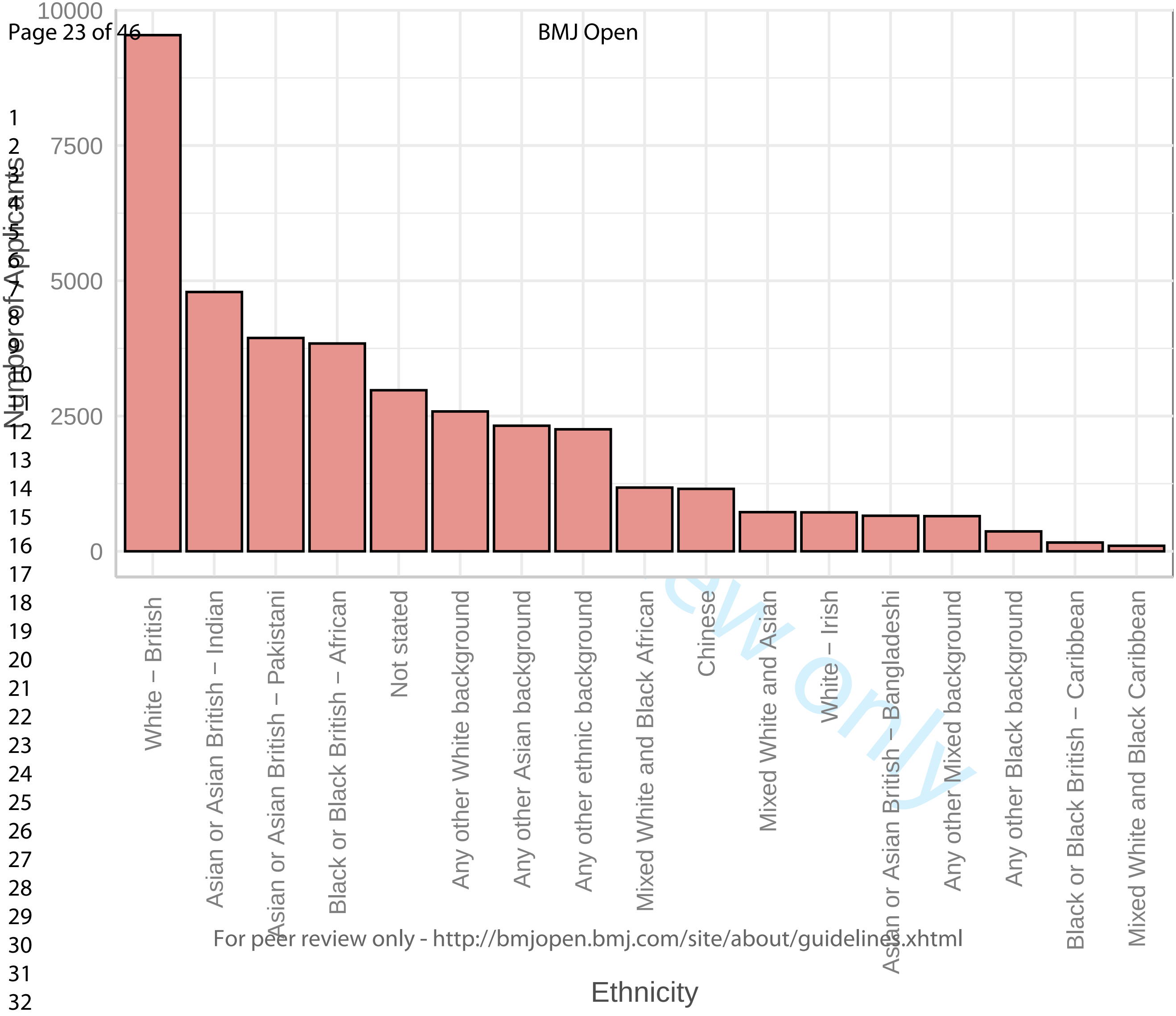
10000
7500
5000
2500
0
Number of Applicants
White – British
Asian or Asian British – Indian
Asian or Asian British – Pakistani
Black or Black British – African
Not stated
Any other White background
Any other Asian background
Any other ethnic background
Mixed White and Black African
Chinese
Mixed White and Asian
White – Irish
Asian or Asian British – Bangladeshi
Any other Mixed background
Any other Black background
Black or Black British – Caribbean
Mixed White and Black Caribbean
Ethnicity

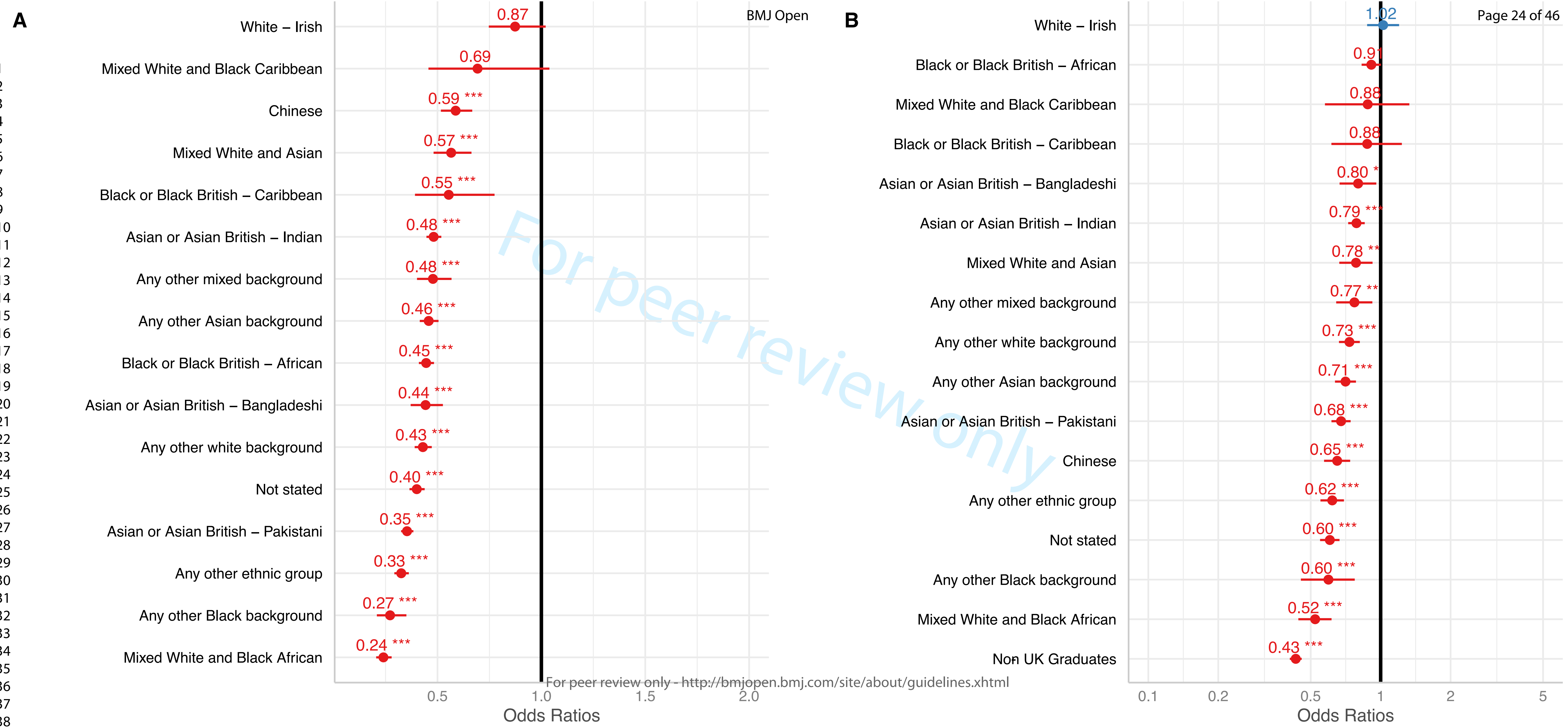

For peer review only

Supplementary Tables

Specialty and Level of Entry	Group
ACCS Internal Medicine/Internal Medicine CT1	Acute
ACCS Anaesthetics/Core Anaesthetics CT1	Anaes/ICU
Anaesthetics ST3	Anaes/ICU
Intensive Care Medicine ST3	Anaes/ICU
ACCS Emergency Medicine ST1/CT1	EM
Emergency Medicine ST3	EM
Emergency Medicine ST4	EM
General Practice ST1	GP
Allergy ST3	Medical
Audiovestibular Medicine ST3	Medical
Cardiology ST3	Medical
Clinical Genetics ST3	Medical
Clinical Neurophysiology ST3	Medical
Clinical Oncology ST3	Medical
Combined Infection Training ST3	Medical
Dermatology ST3	Medical
Gastroenterology ST3	Medical
Genitourinary Medicine ST3	Medical
Haematology ST3	Medical
Immunology ST3	Medical
Medical Oncology ST3	Medical
Medical Ophthalmology ST3	Medical
Neurology ST3	Medical
Occupational Medicine ST3	Medical
Palliative Medicine ST3	Medical
Rehabilitation Medicine ST3	Medical
Sport and Exercise Medicine ST3	Medical
Community Sexual and Reproductive Health ST1	Obs/Gynae
Obstetrics and Gynaecology ST1	Obs/Gynae
Obstetrics and Gynaecology ST3	Obs/Gynae
Ophthalmology ST1	Ophthalmology
Ophthalmology ST3	Ophthalmology
Paediatric Cardiology ST4	Paediatric Medical
Paediatrics ST1	Paediatric Medical
Paediatrics ST3	Paediatric Medical
Paediatrics ST4	Paediatric Medical
Chemical Pathology ST3	Pathology
Diagnostic Neuropathology ST3	Pathology
Histopathology ST1	Pathology
Core Psychiatry CT1	Psychiatry
General Psychiatry ST4	Psychiatry
Public Health Medicine ST1	Public Health
Clinical Radiology ST1	Radiology
Cardiothoracic Surgery ST1	Surgical
Cardiothoracic Surgery ST3	Surgical
Core Surgical Training CT1	Surgical
General and Vascular Surgery ST3	Surgical
Neurosurgery ST1	Surgical
Neurosurgery ST2	Surgical
Oral and Maxillo Facial Surgery ST1	Surgical
Oral and Maxillo Facial Surgery ST3	Surgical

For peer review only

Otolaryngology ST3	Surgical
Paediatric Surgery ST3	Surgical
Plastic Surgery ST3	Surgical
Trauma and Orthopaedic Surgery ST3	Surgical
Urology ST3	Surgical

Supplementary Table 1: Grouping of individual specialty posts for onward analysis
Abbreviations: ST, Specialist Trainee (year); CT, Core Trainee (year); ACCS, Acute Care Common Stem.

Specialty and Level of Entry	Male	Female	Not Stated	Total
General Practice ST1	4675	5128	250	10053
ACCS Internal Medicine/Internal Medicine CT1	1772	1723	95	3590
Core Surgical Training CT1	1486	932	110	2528
ACCS Anaesthetics/Core Anaesthetics CT1	1364	1026	67	2457
Core Psychiatry CT1	964	1092	74	2130
Clinical Radiology ST1	1146	637	96	1879
Anaesthetics ST3	930	660	37	1627
ACCS Emergency Medicine ST1/CT1	806	576	42	1424
Public Health Medicine ST1	315	644	29	988
Paediatrics ST1	272	598	16	886
Obstetrics and Gynaecology ST1	230	622	25	877
General Psychiatry ST4	284	353	8	645
Ophthalmology ST1	355	238	32	625
General and Vascular Surgery ST3	380	215	12	607
Trauma and Orthopaedic Surgery ST3	489	98	17	604
Intensive Care Medicine ST3	392	192	11	595
Cardiology ST3	284	102	15	401
Haematology ST3	174	184	10	368
Histopathology ST1	156	180	15	351
Obstetrics and Gynaecology ST3*	94	215	<5	309
Dermatology ST3	63	218	12	293
Combined Infection Training ST3	138	128	6	272
Clinical Oncology ST3	127	130	10	267
Gastroenterology ST3*	187	63	<5	250
Neurosurgery ST1	165	67	10	242
Emergency Medicine ST3	189	42	6	237
Paediatrics ST3*	114	106	<5	220
Medical Oncology ST3	91	113	8	212
Plastic Surgery ST3	113	87	11	211
Urology ST3	136	57	6	199
Paediatrics ST4	93	82	0	175
Neurology ST3	97	62	9	168
Community Sexual and Reproductive Health ST1	40	120	7	167

For peer review only

Otolaryngology ST3*	91	61	<5	152
Emergency Medicine ST4*	87	49	<5	136
Cardiothoracic Surgery ST1*	69	40	<5	109
Palliative Medicine ST3*	31	70	<5	101
Ophthalmology ST3*	47	33	<5	80
Paediatric Surgery ST3*	40	38	<5	78
Rehabilitation Medicine ST3*	45	29	<5	74
Immunology ST3*	31	42	<5	73
Neurosurgery ST2*	41	17	<5	58
Clinical Genetics ST3	13	43	0	56
Oral and Maxillo Facial Surgery ST1*	31	19	<5	50
Clinical Neurophysiology ST3*	32	16	<5	48
Cardiothoracic Surgery ST3*	38	8	<5	46
Genitourinary Medicine ST3*	21	25	<5	46
Occupational Medicine ST3*	26	19	<5	45
Allergy ST3*	17	21	<5	38
Paediatric Cardiology ST4*	24	10	<5	34
Sport and Exercise Medicine ST3*	26	7	<5	33
Chemical Pathology ST3	16	13	0	29
Oral and Maxillo Facial Surgery ST3*	18	6	<5	24
Medical Ophthalmology ST3	12	9	0	21
Audiovestibular Medicine ST3*	11	5	<5	16
Diagnostic Neuropathology ST3	8	8	0	16

Supplementary Table 2: The number of applicants to Specialty Posts in the 2021-2022 recruitment cycle by gender. Totals with * are not exact as <5 applicants did not state their gender. ‘Nuclear Medicine ST3’ and ‘Paediatric and Perinatal Pathology ST3’ did not have 5 or more applicants in a gender category and therefore are not included here. Abbreviations: ST, Specialist Trainee (year); CT, Core Trainee (year); ACCS, Acute Care Common Stem.

For peer review only

Specialty and Level	Male Success (%)	Female Success (%)	Sig diff in male or female success (p-value)
Emergency Medicine ST3	6.35	14.29	0.107
Neurosurgery ST1	6.06	7.46	0.769
Cardiothoracic Surgery ST1	8.70	0.00	0.084
Community Sexual and Reproductive Health ST1	0.00	5.00	0.338
Occupational Medicine ST3	23.08	0.00	0.032
Sport and Exercise Medicine ST3	23.08	0.00	0.301
Chemical Pathology ST3	31.25	38.46	0.714

Supplementary Table 3: Success of applicants to Training Posts by specialty in the 2021 recruitment year by gender where numbers are small. Table showing the applications of trainees to Specialty Training Posts and percentage of successful applicants by gender, where numbers are small and *p*-values derived from Fisher exact tests are provided for comparison and to 3 decimal places. Where p-values fall below the significance threshold of 0.05 they are highlighted in blue (Occupational Medicine ST3, which had a higher proportion of successful males).

Ethnic.Origin	Total	Non–UK Graduate Success (%)	UK Graduate Success (%)	Percentage Difference (95% CI)
Asian or Asian British – Bangladeshi	645	21.4	51.8	30.40 (21.50 to 39.30)
Asian or Asian British – Indian	4703	23.2	44.1	20.90 (18.00 to 23.70)
Asian or Asian British – Pakistani	3907	21.3	39.8	18.50 (14.60 to 22.40)
Chinese	1141	28.9	36.5	7.57 (–0.18 to 15.30)
Any other Asian background	2288	24.2	37.2	13.00 (9.06 to 16.90)
Black or Black British – African	3736	28.0	38.9	10.90 (6.02 to 15.80)
Black or Black British – Caribbean	150	23.9	48.4	24.50 (7.85 to 41.20)
Any other Black background	362	19.1	43.8	24.70 (–3.25 to 52.60)
Mixed White and Asian	705	21.0	44.8	23.80 (16.80 to 30.70)
Mixed White and Black African	1158	17.3	36.8	19.60 (5.94 to 33.20)
Mixed White and Black Caribbean	97	22.6	47.0	24.40 (3.00 to 45.80)
Any other mixed background	637	22.1	44.7	22.50 (14.70 to 30.30)
White – British	9101	31.1	48.4	17.30 (8.51 to 26.10)
White – Irish	685	34.0	47.8	13.80 (4.58 to 22.90)
Any other white background	2530	23.2	40.2	17.00 (13.00 to 21.10)
Any other ethnic group	2217	19.5	39.0	19.50 (14.40 to 24.60)
Not stated	2921	19.3	36.7	17.40 (14.10 to 20.80)
All Ethnicities*	36983	22.8	44.5	21.80 (20.80 to 22.70)

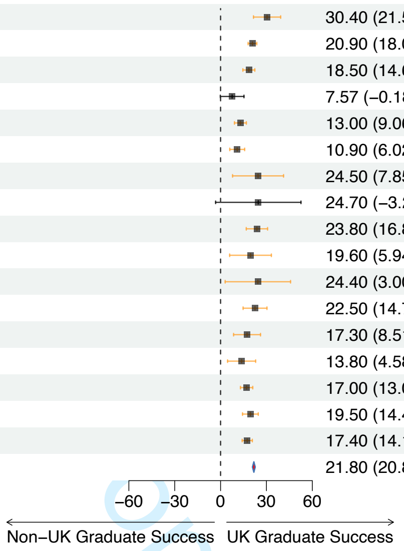

Supplementary Table 4: Success of applicants to Training Posts by ethnicity in the 2021 recruitment year by graduate status. Table showing the applications of trainees to Specialty Training Posts by ethnicity and percentage of successful applicants by graduate status (UK vs Non-UK). All values are provided to 3 significant figures. Where confidence intervals for estimated differences between the percentage of successful applicants by graduate status does not cross zero, it is highlighted in orange (greater UK graduate success).

Predictors	Adjusted Odds Ratios	95% Confidence Interval	p
Non-UK Graduates	0.43 ***	0.41 – 0.46	**<0.001**
White – Irish	1.02	0.87 – 1.20	0.762
Any other White background	0.73 ***	0.66 – 0.81	**<0.001**
Mixed White and Black Caribbean	0.88	0.58 – 1.33	0.546
Mixed White and Black African	0.52 ***	0.44 – 0.61	**<0.001**
Mixed White and Asian]	0.78 **	0.66 – 0.92	**0.004**
Black or Black British - Caribbean	0.88	0.61 – 1.23	0.453
Black or Black British - African	0.91	0.83 – 1.00	0.058
Any other Black background	0.60 ***	0.45 – 0.77	**<0.001**
Chinese	0.65 ***	0.57 – 0.74	**<0.001**
Asian or Asian British - Bangladeshi	0.80 *	0.67 – 0.96	**0.016**
Asian or Asian British - Indian	0.79 ***	0.72 – 0.85	**<0.001**
Asian or Asian British - Pakistani	0.68 ***	0.61 – 0.74	**<0.001**
Any other Asian background]	0.71 ***	0.64 – 0.78	**<0.001**
Any other mixed background	0.77 **	0.64 – 0.92	**0.004**
Any other ethnic group	0.62 ***	0.55 – 0.69	**<0.001**
Not stated	0.60 ***	0.55 – 0.67	**<0.001**
Observations	36983		

Supplementary Table 5: The effect of ethnicity and graduate status on success at application to Specialty Posts in the UK. Multivariable logistic regression model comparing success of application to Specialty Training Posts by minority ethnic groups to White British applicants where Country of Qualification (UK versus Non-UK) is included as a covariate. Non-Medical applicants to Public Health Specialty Training have been removed (n=988). Odds ratios are presented with 95% Confidence Intervals. ** p<0.05 ** p<0.01 *** p<0.001.*

Predictors	Unadjusted Odds Ratios	95% Confidence Interval	p
Non-UK Graduates	0.37 ***	0.35 – 0.38	**<0.001**
White - Irish	0.87	0.75 – 1.02	0.089
Any other white background	0.43 ***	0.39 – 0.47	**<0.001**
Mixed White and Black Caribbean	0.69	0.46 – 1.04	0.079
Mixed White and Black African	0.24 ***	0.20 – 0.28	**<0.001**
Mixed White and Asian	0.57 ***	0.48 – 0.66	**<0.001**
Black or Black British – Caribbean	0.55 ***	0.39 – 0.77	**0.001**
Black or Black British - African	0.45 ***	0.41 – 0.48	**<0.001**
Any other Black background	0.27 ***	0.21 – 0.35	**<0.001**
Chinese	0.59 ***	0.52 – 0.67	**<0.001**
Asian or Asian British - Bangladeshi	0.44 ***	0.37 – 0.53	**<0.001**
Asian or Asian British - Indian	0.48 ***	0.45 – 0.52	**<0.001**
Asian or Asian British - Pakistani	0.35 ***	0.32 – 0.38	**<0.001**
Any other Asian background	0.46 ***	0.41 – 0.50	**<0.001**
Any other mixed background	0.48 ***	0.40 – 0.57	**<0.001**
Any other ethnic group	0.33 ***	0.29 – 0.36	**<0.001**
Not stated	0.40 ***	0.37 – 0.44	**<0.001**
Observations	36983		

Supplementary Table 6: The unadjusted effects of ethnicity and graduate status on success at application to Specialty Posts in the UK. Unadjusted multivariable logistic regression model comparing success of application to Specialty Training Posts by minority ethnic groups to White British applicants where Country of Qualification (UK versus Non-UK) **is not** included as a covariate. Non-Medical applicants to Public Health Specialty Training have been removed (n=988). Odds ratios are presented with 95% Confidence Intervals. ** p<0.05 ** p<0.01 *** p<0.001.*

For peer review only

Ethnicity	UK Graduate probability of success	95% Confidence Interval	Non-UK Graduate probability of success	95% Confidence Interval
White British	0.48	0.47-0.49	0.31	0.23-0.40
White Irish	0.48	0.44-0.52	0.34	0.27-0.42
Any other white background	0.40	0.37-0.44	0.23	0.21-0.25
Mixed White and Black Caribbean	0.47	0.35-0.59	0.23	0.11-0.40
Mixed White and Black African	0.37	0.25-0.50	0.17	0.15-0.20
Mixed White and Asian	0.45	0.40-0.50	0.21	0.17-0.26
Black or Black British Caribbean	0.48	0.36-0.61	0.24	0.16-0.34
Black or Black British African	0.39	0.34-0.43	0.28	0.26-0.30
Any other Black background	0.44	0.22-0.68	0.19	0.15-0.24
Chinese	0.36	0.33-0.40	0.29	0.23-0.36
Asian or Asian British Bangladeshi	0.52	0.44-0.59	0.21	0.18-0.25
Asian or Asian British Indian	0.44	0.42-0.46	0.23	0.22-0.25
Asian or Asian British Pakistani	0.40	0.36-0.43	0.21	0.20-0.23
Any other Asian background	0.37	0.34-0.40	0.24	0.22-0.27
Any other mixed background	0.45	0.39-0.51	0.22	0.18-0.27
Any other ethnic group	0.39	0.34-0.44	0.20	0.18-0.21
Not stated	0.37	0.34-0.39	0.19	0.17-0.21

Supplementary Table 7: Multivariable logistic regression model displaying the probability of success of application to Specialty Training Posts in the 2021-22 recruitment cycle by Ethnicity and Country of Graduate Status. A logistic regression model is fitted with variation of success at application between ethnic groups by country of qualification measured by including interaction terms between the two variables. Probabilities are presented with 95% Confidence Intervals. Anova analysis of residuals between models where an interaction was included and where it was not, demonstrated a significant difference between the models. Only the 'Asian or Asian British – Bangladeshi' group significantly differed in outcome across the graduate status (p=0.02).

For peer review only

Supplementary Figures

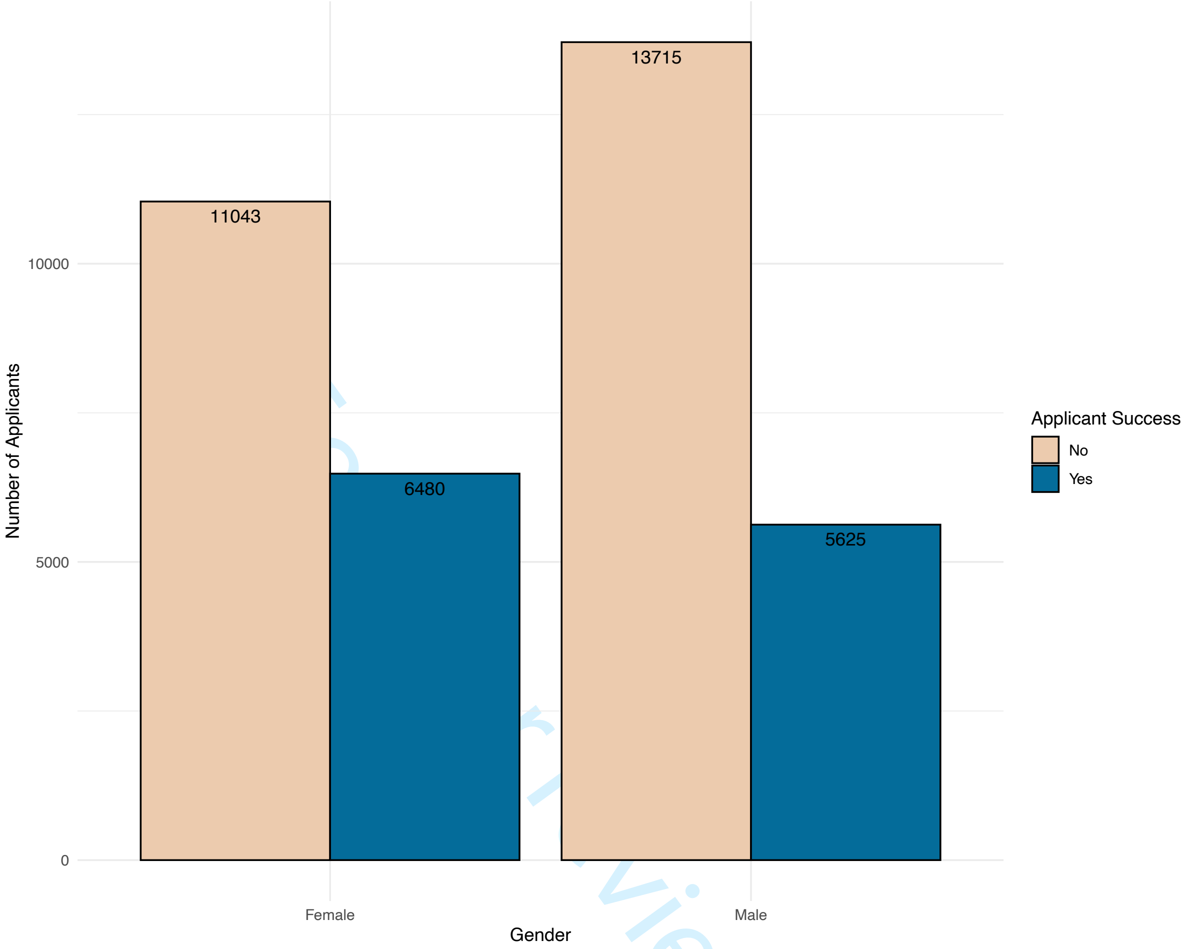

Supplementary Figure 1: Applications and outcomes for Specialty Training Posts in the 2021-22 recruitment year by gender. Bar chart demonstrating applications and outcomes of trainees to Specialty Training by gender. Overall, the difference in percentage of success by females (6480/17523, 37.0%) and males (5625/19340, 29.1%) was 7.9% (95% C.I.= 6.93-8.86%), in favour of females.

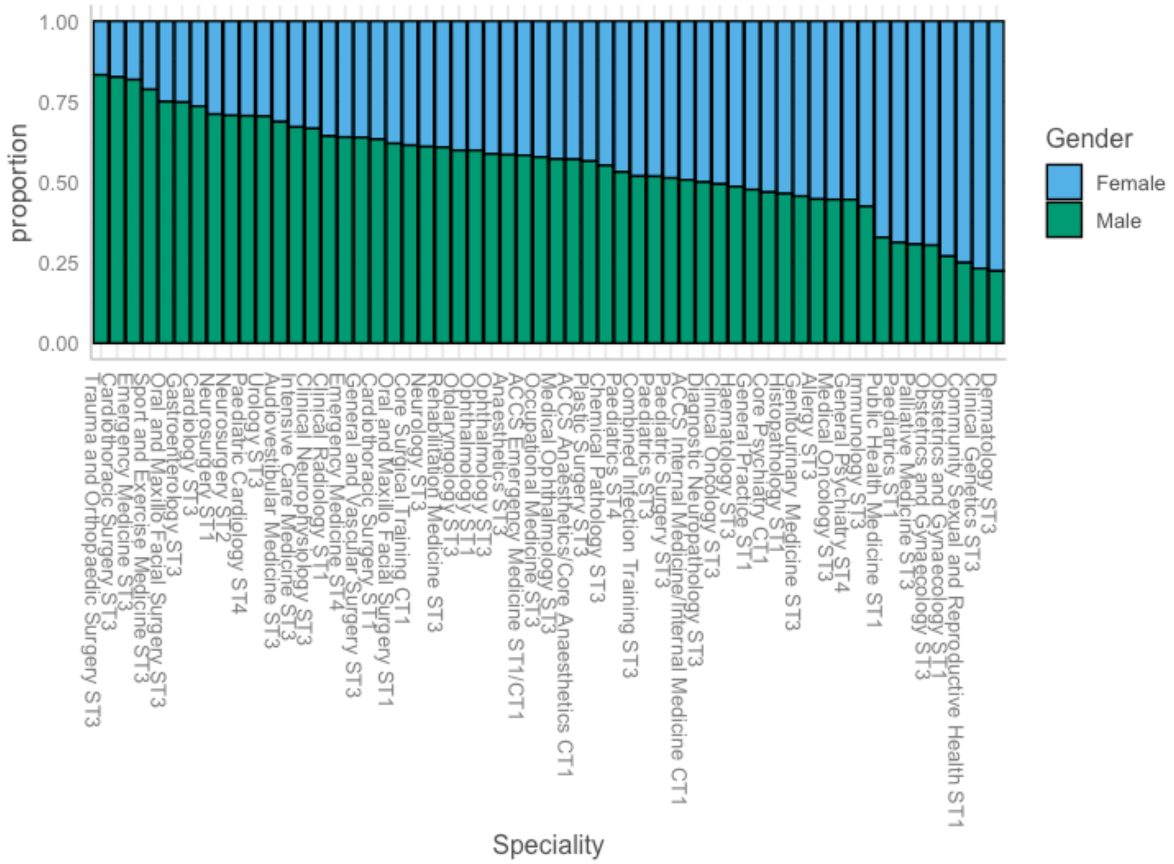

Supplementary Figure 2: Proportion of applicants to individual Specialty Training Posts in the 2021 recruitment year by Gender. Data is presented where complete.

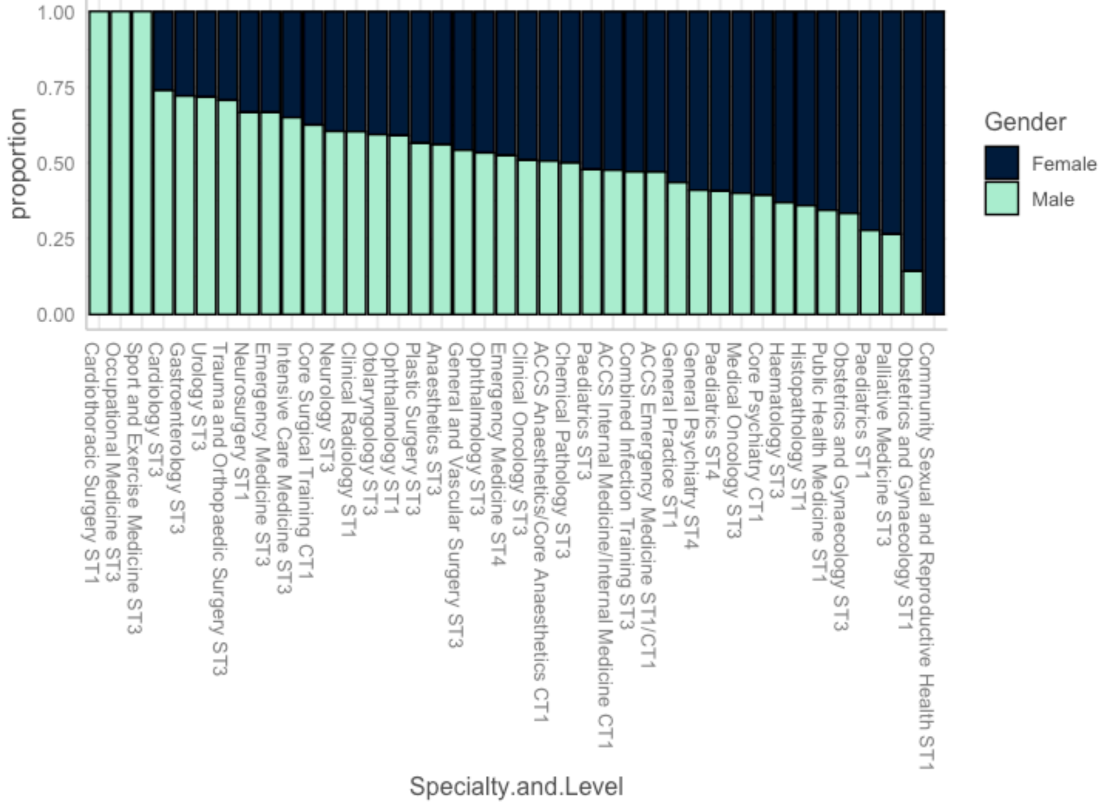

Supplementary Figure 3: Proportion of successful applicants to individual specialities in the 2021 recruitment year by Gender. Data is presented where complete.

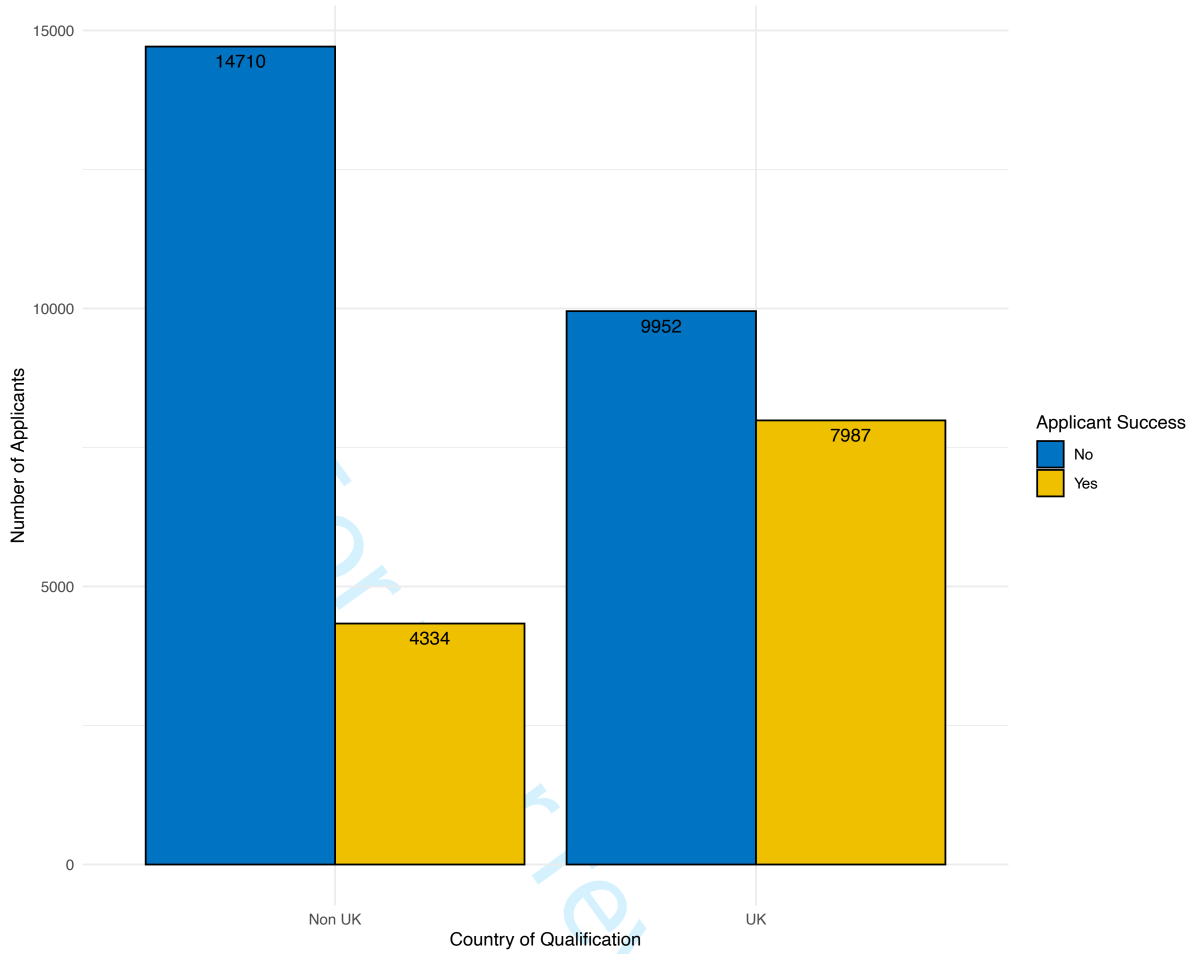

Supplementary Figure 4: Applications and outcomes for Specialty Training Posts in the 2021-2022 recruitment year by ethnicity. (a) Applications to Specialty Training Posts in the 2021 recruitment year by Ethnicity. (b**)** Applications and outcomes by country of qualification (Non-UK vs UK). Overall, the difference in percentage of success by UK graduates (7987/17939, 44.5%) and Non-UK graduates (4334/19044, 22.8%) was 21.8% (95% C.I. = 20.8-22.7%), in favour of UK graduates.

For peer review only

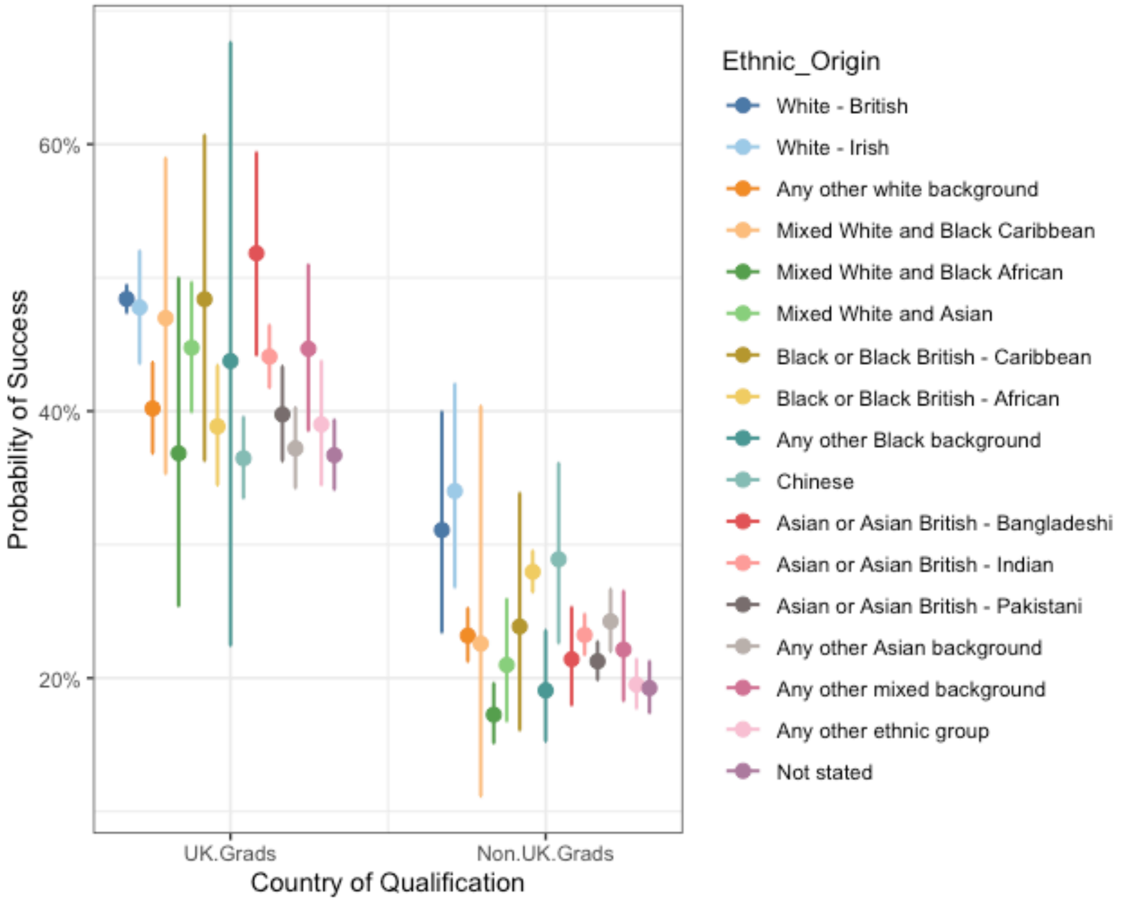

Supplementary Figure 5: Multivariable logistic regression model displaying the probability of success of application to Specialty Training Posts in the 2021-22 recruitment cycle by Ethnicity. A logistic regression model is fitted with variation of success at application between ethnic groups by country of qualification measured by including interaction terms between the two variables. Probabilities are presented with 95% Confidence Intervals. Anova analysis of residuals between models where an interaction was included and where it was not, demonstrated a significant difference between the models. Only the ‘Asian or Asian British – Bangladeshi’ group significantly differed in outcome across the graduate status.

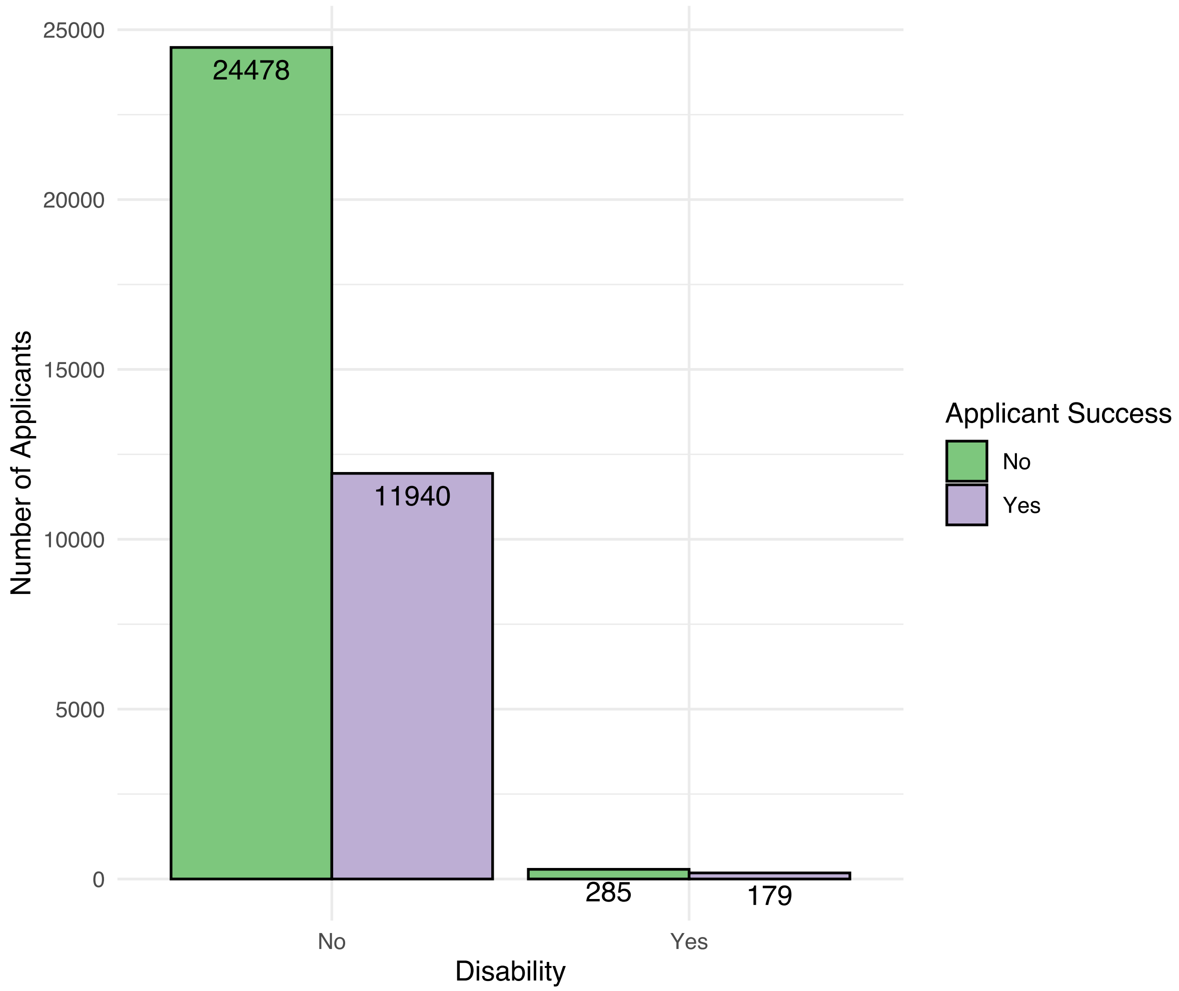

Supplementary Figure 6: **Applications and outcomes to Specialty Training Posts in 2021 by disability status.** Overall, the difference in percentage of success by disabled applicants (179/464, 38.6%) and non-disabled applicants (11940/36418, 32.8%) was 5.79% (95% C.I. = 1.23-10.4%), in favour of disabled applicants.

Gender – All applicants

Gender	2021 All Applicants	2021 Accepted Applicants
Male	19340	5625
Female	17523	6480
Not Stated	1108	314

Country of Qualification – All applicants

Country of Qualification	2021 All Applicants	2021 Accepted Applicants
UK	17939	7987
Non UK	19044	4334
Non Medical Public Health Applicants	988	98

Ethnicity by Graduate Country

Ethnic Origin	Applied			Accepted		
	UK Grads	Non-UK Grads	Non-Medical Applicants	UK Grads	Non-UK Grads	Non-Medical Applicants
Asian or Asian British - Bangladeshi	164	481	12	85	103	0
Asian or Asian British - Indian	1733	2970	90	764	690	<5
Asian or Asian British - Pakistani	732	3175	36	291	675	<5
Chinese	968	173	13	353	50	<5
Any other Asian background	989	1299	33	368	315	<5
Black or Black British - African	453	3283	105	176	918	<5
Black or Black British - Caribbean	62	88	12	30	21	0
Any other Black background	16	346	6	7	66	0
Mixed White and Asian	400	305	19	179	64	<5
Mixed White and Black African	57	1101	20	21	190	0
Mixed White and Black Caribbean	66	31	5	31	7	0
Any other mixed background	244	393	13	109	87	<5
White - British	8982	119	440	4348	37	68
White - Irish	538	147	35	257	50	<5
Any other white background	796	1734	55	320	402	5
Any other ethnic group	423	1794	38	165	350	<5
Not stated	1316	1605	56	483	309	<5

For peer review only

Disability - All applicants

Disability	2021 All Applicants	2021 Accepted Applicants
Yes	464	179
No	36418	11940
Not Stated	1089	300

Specialty by Gender

Specialty and Level	Number of Applicants			Number Accepted An Offer		
	Male	Female	Not Stated	Male	Female	Not Stated
ACCS Anaesthetics/Core Anaesthetics CT1	1364	1026	67	323	315	10
ACCS Emergency Medicine ST1/CT1	806	576	42	165	186	9
ACCS Internal Medicine/Internal Medicine CT1	1772	1723	95	728	801	38
Allergy ST3	17	21	<5	<5	<5	0
Anaesthetics ST3	930	660	37	315	247	15
Audiovestibular Medicine ST3	11	5	<5	<5	0	0
Cardiology ST3	284	102	15	68	24	<5
Cardiothoracic Surgery ST1	69	40	<5	6	0	0
Cardiothoracic Surgery ST3	38	8	<5	<5	0	0
Chemical Pathology ST3	16	13	0	5	5	0
Clinical Genetics ST3	13	43	0	<5	14	0
Clinical Neurophysiology ST3	32	16	<5	7	<5	0
Clinical Oncology ST3	127	130	10	55	53	<5
Clinical Radiology ST1	1146	637	96	222	146	22
Combined Infection Training ST3	138	128	6	32	36	0
Community Sexual and Reproductive Health ST1	40	120	7	0	6	0
Core Psychiatry CT1	964	1092	74	258	398	27
Core Surgical Training CT1	1486	932	110	332	199	23
Dermatology ST3	63	218	12	<5	35	<5
Diagnostic Neuropathology ST3	8	8	0	0	<5	0
Emergency Medicine ST3	189	42	6	12	6	<5
Emergency Medicine ST4	87	49	<5	32	29	0
Gastroenterology ST3	187	63	<5	31	12	<5
General and Vascular Surgery ST3	380	215	12	71	60	0
General Practice ST1	4675	5128	250	1952	2532	96
General Psychiatry ST4	284	353	8	178	256	8
Genitourinary Medicine ST3	21	25	<5	<5	9	0
Haematology ST3	174	184	10	31	53	<5
Histopathology ST1	156	180	15	37	66	6

For peer review only

Immunology ST3	31	42	<5	<5	6	0
Intensive Care Medicine ST3	392	192	11	128	69	5
Medical Oncology ST3	91	113	8	30	45	<5
Medical Ophthalmology ST3	12	9	0	<5	<5	0
Neurology ST3	97	62	9	29	19	<5
Neurosurgery ST1	165	67	10	10	5	0
Neurosurgery ST2	41	17	<5	0	<5	0
Nuclear Medicine ST3	13	<5	0	<5	<5	0
Obstetrics and Gynaecology ST1	230	622	25	36	216	8
Obstetrics and Gynaecology ST3	94	215	<5	7	14	<5
Occupational Medicine ST3	26	19	<5	6	0	0
Ophthalmology ST1	355	238	32	49	34	6
Ophthalmology ST3	47	33	<5	8	7	0
Oral and Maxillo Facial Surgery ST1	31	19	<5	<5	5	<5
Oral and Maxillo Facial Surgery ST3	18	6	<5	8	<5	<5
Otolaryngology ST3	91	61	<5	19	13	<5
Paediatric and Perinatal Pathology ST3	<5	7	0	0	<5	0
Paediatric Cardiology ST4	24	10	<5	5	<5	0
Paediatric Surgery ST3	40	38	<5	<5	7	0
Paediatrics ST1	272	598	16	116	302	5
Paediatrics ST3	114	106	<5	11	12	0
Paediatrics ST4	93	82	0	11	16	0
Palliative Medicine ST3	31	70	<5	9	25	0
Plastic Surgery ST3	113	87	11	39	30	<5
Public Health Medicine ST1	315	644	29	33	63	<5
Rehabilitation Medicine ST3	45	29	<5	13	<5	0
Sport and Exercise Medicine ST3	26	7	<5	6	0	0
Trauma and Orthopaedic Surgery ST3	489	98	17	123	51	<5
Urology ST3	136	57	6	33	13	<5

Specialty by Disability

Specialty and Level	Number of Applicants			Number Accepted An Offer		
	Disabled	Not Disabled	Not Stated	Disabled	Not Disabled	Not Stated
ACCS Anaesthetics/Core Anaesthetics CT1	24	2373	60	6	631	11
ACCS Emergency Medicine ST1/CT1	9	1374	41	<5	351	8
ACCS Internal Medicine/Internal Medicine CT1	36	3457	97	17	1516	34
Allergy ST3	<5	37	<5	0	5	0
Anaesthetics ST3	19	1585	23	5	563	9
Audiovestibular Medicine ST3	0	16	<5	0	<5	<5
Cardiology ST3	<5	384	18	<5	90	6

For peer review only

Cardiothoracic Surgery ST1	<5	109	<5	0	6	0
Cardiothoracic Surgery ST3	0	44	<5	0	<5	<5
Chemical Pathology ST3	<5	26	<5	<5	9	0
Clinical Genetics ST3	<5	55	0	0	18	0
Clinical Neurophysiology ST3	<5	35	<5	<5	7	0
Clinical Oncology ST3	<5	260	6	0	107	<5
Clinical Radiology ST1	18	1785	506	7	369	14
Combined Infection Training ST3	<5	246	5	0	65	<5
Community Sexual and Reproductive Health ST1	<5	153	11	0	5	<5
Core Psychiatry CT1	57	2000	73	23	639	21
Core Surgical Training CT1	21	2410	97	8	519	27
Dermatology ST3	<5	277	12	<5	40	<5
Diagnostic Neuropathology ST3	<5	15	0	0	<5	0
Emergency Medicine ST3	<5	229	6	0	19	<5
Emergency Medicine ST4	5	130	<5	<5	57	<5
Gastroenterology ST3	0	248	6	0	42	<5
General and Vascular Surgery ST3	7	585	15	0	130	<5
General Practice ST1	121	9632	300	61	4412	107
General Psychiatry ST4	16	618	11	15	418	9
Genitourinary Medicine ST3	<5	47	<5	0	13	0
Haematology ST3	<5	358	9	0	83	<5
Histopathology ST1	11	330	10	6	101	<5
Immunology ST3	<5	73	<5	0	9	0
Intensive Care Medicine ST3	<5	582	10	<5	196	<5
Medical Oncology ST3	<5	206	5	<5	76	<5
Medical Ophthalmology ST3	0	21	0	0	<5	0
Neurology ST3	<5	155	11	0	47	<5
Neurosurgery ST1	<5	233	6	<5	15	0
Neurosurgery ST2	<5	55	<5	0	<5	0
Nuclear Medicine ST3	0	16	0	0	<5	0
Obstetrics and Gynaecology ST1	7	844	26	<5	250	6
Obstetrics and Gynaecology ST3	0	305	7	0	21	<5
Occupational Medicine ST3	<5	43	<5	0	6	0
Ophthalmology ST1	7	572	26	<5	86	<5
Ophthalmology ST3	<5	79	<5	0	15	0
Oral and Maxillo Facial Surgery ST1	<5	51	<5	<5	9	0
Oral and Maxillo Facial Surgery ST3	0	27	<5	0	15	0
Otolaryngology ST3	<5	149	<5	0	33	0
Paediatric and Perinatal Pathology ST3	0	11	0	0	<5	0
Paediatric Cardiology ST4	0	34	<5	0	6	0
Paediatric Surgery ST3	0	77	<5	0	8	<5
Paediatrics ST1	11	851	24	9	408	6

For peer review only

Paediatrics ST3	0	218	<5	0	25	0
Paediatrics ST4	0	174	<5	0	27	0
Palliative Medicine ST3	<5	99	<5	0	33	<5
Plastic Surgery ST3	0	206	5	0	71	<5
Public Health Medicine ST1	46	919	23	<5	93	<5
Rehabilitation Medicine ST3	<5	69	6	0	16	0
Sport and Exercise Medicine ST3	<5	34	0	<5	5	0
Trauma and Orthopaedic Surgery ST3	<5	588	13	0	175	<5
Urology ST3	<5	196	<5	0	48	0

For peer review only

Specialty by Ethnicity

Specialty and Level	Number of Applicants																	Number Accepted An Offer																
	Asian or Asian British - Bangladeshi	Asian or Asian British - Indian	Asian or Asian British - Pakistani	Chinese	Any other Asian background	Black or Black British - African	Black or Black British - Caribbean	Any other Black background	Mixed White and Asian	Mixed White and Black African	Mixed White and Black Caribbean	Any other Mixed background	White - British	White - Irish	Any other White background	Any other ethnic group	Not Stated	Asian or Asian British - Bangladeshi	Asian or Asian British - Indian	Asian or Asian British - Pakistani	Chinese	Any other Asian background	Black or Black British - African	Black or Black British - Caribbean	Any other Black background	Mixed White and Asian	Mixed White and Black African	Mixed White and Black Caribbean	Any other Mixed background	White - British	White - Irish	Any other White background	Any other ethnic group	Not Stated
ACCS Anaesthetics/Core Anaesthetics CT1	21	225	121	102	129	98	9	5	46	37	13	38	1135	79	162	77	160	<5	41	14	26	19	5	<5	0	9	<5	6	12	424	29	31	5	22
ACCS Emergency Medicine ST1/CT1	14	190	99	31	75	113	5	<5	33	33	<5	29	492	24	97	69	113	<5	18	9	10	6	12	<5	0	13	<5	<5	9	226	11	21	<5	15
ACCS Internal Medicine/Internal Medicine CT1	59	456	327	172	266	244	9	19	94	66	10	47	1062	79	269	161	250	26	192	111	79	112	91	<5	6	43	21	6	21	577	30	112	56	81
Allergy ST3	<5	7	6	<5	<5	6	0	<5	0	<5	0	<5	<5	0	<5	<5	5	0	0	0	<5	0	0	0	0	0	0	0	0	<5	0	<5	0	<5
Anaesthetics ST3	<5	193	38	49	56	18	<5	<5	39	20	<5	27	893	43	94	66	81	<5	58	<5	19	12	<5	<5	<5	14	<5	<5	12	350	20	33	12	31
Audiovestibular Medicine ST3	0	<5	<5	<5	<5	<5	0	<5	0	<5	0	0	0	0	<5	<5	<5	0	<5	0	<5	0	<5	0	0	0	0	0	0	0	0	0	0	0
Cardiology ST3	11	48	68	9	30	35	<5	<5	5	19	0	12	44	5	38	29	41	<5	15	7	<5	8	6	<5	0	<5	0	0	0	25	<5	10	0	11
Cardiothoracic Surgery ST1	<5	14	10	9	10	9	0	<5	5	<5	<5	0	10	<5	19	<5	16	0	<5	0	<5	<5	0	0	0	0	0	0	0	<5	0	0	0	0
Cardiothoracic Surgery ST3	<5	0	<5	<5	7	6	0	0	0	<5	<5	0	6	<5	<5	6	6	0	0	0	0	0	0	0	0	0	0	0	0	0	0	0	<5	<5
Chemical Pathology ST3	0	<5	<5	0	0	<5	0	<5	0	<5	0	<5	0	0	0	<5	0	0	0	<5	0	0	<5	0	0	0	<5	0	0	0	0	0	0	0
Clinical Genetics ST3	0	6	<5	<5	5	6	0	<5	<5	0	<5	0	17	<5	5	<5	<5	0	<5	0	<5	<5	0	0	0	<5	0	0	<5	8	0	<5	<5	0
Clinical Neurophysiology ST3	<5	<5	9	<5	<5	5	0	<5	0	<5	<5	<5	<5	<5	<5	<5	<5	0	0	<5	0	0	<5	0	0	0	<5	0	<5	<5	<5	<5	0	0
Clinical Oncology ST3	<5	32	23	7	14	10	0	<5	<5	7	<5	<5	60	<5	7	14	14	0	16	6	<5	6	<5	0	<5	<5	<5	0	<5	35	<5	<5	6	5
Clinical Radiology ST1	32	288	255	79	137	185	10	17	26	44	6	39	226	26	145	164	200	<5	57	29	25	22	18	0	<5	7	<5	<5	8	116	7	31	19	38

For peer review only

Combined Infection Training ST3	<5	28	33	5	16	30	<5	11	5	19	0	11	59	<5	10	5	19	0	7	5	<5	5	5	<5	<5	<5	<5	0	<5	28	0	<5	0	<5
Community Sexual and Reproductive Health ST1	5	22	15	<5	9	27	<5	<5	<5	<5	<5	<5	32	6	9	10	18	0	0	0	0	0	<5	0	0	0	0	0	0	<5	<5	0	0	0
Core Psychiatry CT1	48	234	249	36	125	358	8	23	40	86	7	40	363	17	150	158	188	14	76	41	15	27	93	<5	<5	19	12	<5	17	204	8	49	53	46
Core Surgical Training CT1	45	292	185	117	167	168	13	12	47	31	<5	42	694	56	243	147	265	9	75	34	26	40	26	<5	<5	15	<5	<5	7	189	11	42	26	48
Dermatology ST3	<5	43	36	25	20	18	<5	<5	8	6	<5	<5	48	9	24	24	22	<5	6	<5	<5	<5	<5	0	0	<5	0	<5	0	9	0	<5	5	5
Diagnostic Neuropathology ST3	0	5	0	0	<5	<5	0	<5	0	<5	0	<5	<5	0	<5	0	0	0	0	0	0	0	0	0	0	0	0	0	<5	0	0	<5	0	0
Emergency Medicine ST3	<5	88	19	<1	10	32	0	<5	<5	17	0	6	9	<5	11	15	19	0	5	<5	0	<5	<5	0	<5	<5	<5	0	0	<5	0	0	<5	<5
Emergency Medicine ST4	0	34	12	<5	<5	<5	0	<5	<5	<5	<5	0	53	<5	8	<5	7	0	9	<5	<5	<5	<5	0	0	<5	0	<5	0	33	<5	5	0	<5
Gastroenterology ST3	6	32	52	5	18	22	0	15	<5	19	<5	9	19	<5	11	21	18	<5	5	5	<5	<5	8	0	<5	0	<5	0	0	8	<5	<5	<5	<5
General and Vascular Surgery ST3	12	77	55	15	31	34	<5	8	9	29	<5	13	142	15	50	73	40	<5	9	5	<5	7	5	<5	0	<5	<5	0	<5	66	8	12	<5	6
General Practice ST1	236	1211	1474	213	725	1533	47	109	187	309	17	146	1834	169	517	568	758	104	530	568	96	304	681	25	44	67	125	7	64	1123	109	202	203	328
General Psychiatry ST4	8	107	39	21	28	68	<5	<5	7	6	0	7	210	12	65	30	32	<5	87	23	15	20	40	0	<5	<5	<5	0	6	153	6	38	21	21
Genitourinary Medicine ST3	<5	6	6	<5	<5	6	0	<5	<5	<5	0	<5	7	<5	<5	<5	6	0	<5	0	0	0	<5	0	0	<5	0	0	0	<5	<5	0	<5	0
Haematology ST3	7	59	39	13	12	37	<5	12	6	23	<5	12	57	5	24	30	30	0	20	<5	6	<5	<5	<5	0	<5	<5	<5	<5	30	<5	5	<5	<5
Histopathology ST1	<5	32	20	12	14	41	<5	6	8	12	<5	7	87	5	42	34	26	0	12	5	7	<5	5	<5	0	5	<5	0	<5	42	<5	10	6	8
Immunology ST3	<5	7	6	<5	<5	14	0	5	0	10	0	8	<5	<5	<5	<5	9	0	<5	<5	0	0	<5	0	0	0	0	0	<5	<5	0	0	0	<5
Intensive Care Medicine ST3	<5	72	29	20	19	18	<5	<5	12	15	<5	6	285	14	34	26	33	0	24	<5	6	<5	<5	0	0	6	<5	<5	<5	115	5	10	8	13
Medical Oncology ST3	<5	36	32	8	11	10	0	<5	<5	8	0	<5	48	5	10	14	21	0	14	13	<5	<5	<5	0	0	0	0	0	<5	22	<5	<5	<5	8
Medical Ophthalmology ST3	0	<5	6	0	<5	<5	0	0	<5	0	0	0	<5	0	<5	<5	6	0	0	<5	0	0	0	0	0	0	0	0	0	0	0	<5	0	0
Neurology ST3	6	19	27	<5	10	18	0	5	<5	13	<5	13	20	<5	13	9	16	<5	<5	<5	<5	<5	<5	0	<5	0	<5	0	<5	13	<5	7	0	6
Neurosurgery ST1	6	38	25	9	20	15	<5	<5	<5	5	<5	5	41	<5	31	11	26	0	<5	0	0	<5	<5	0	0	0	0	0	<5	6	0	<5	<5	0
Neurosurgery ST2	<5	<5	8	<5	<5	9	<5	0	0	<5	0	<5	<5	0	10	6	<5	0	0	0	0	0	0	0	0	0	0	0	<5	0	0	0	0	0

For peer review only

Nuclear Medicine ST3	0	<5	<5	0	<5	<5	0	0	0	0	0	0	0	0	<5	<5	<5	0	0	0	0	0	0	0	0	0	0	0	0	0	0	<5	<5	0
Obstetrics and Gynaecology ST1	15	85	43	25	52	146	11	6	19	27	<5	13	206	12	87	48	78	<5	20	7	6	17	21	5	<5	11	<5	<5	<5	114	5	18	7	20
Obstetrics and Gynaecology ST3	<5	79	41	0	8	46	<5	9	6	23	<5	5	<5	0	27	34	22	0	13	<5	0	<5	<5	0	0	0	0	0	0	0	0	<5	<5	<5
Occupational Medicine ST3	0	<5	<5	0	<5	6	0	<5	0	<5	0	0	7	0	<5	0	<5	0	0	0	0	0	<5	0	0	0	0	0	0	<5	0	<5	0	<5
Ophthalmology ST1	7	74	72	30	51	37	<5	<5	7	14	<5	13	72	15	66	68	71	0	13	<5	5	6	<5	0	0	<5	0	0	<5	24	<5	12	13	6
Ophthalmology ST3	0	18	<5	<5	<5	6	<5	0	<5	<5	0	<5	<5	0	9	16	14	0	<5	<5	0	0	<5	<5	0	0	0	0	0	0	0	<5	5	<5
Oral and Maxillo Facial Surgery ST1	<5	12	<5	0	<5	<5	0	0	<5	<5	0	<5	13	<5	5	5	<5	0	<5	0	0	<5	0	0	0	<5	0	0	0	<5	<5	0	<5	0
Oral and Maxillo Facial Surgery ST3	0	<5	<5	<5	<5	0	0	0	0	0	0	0	6	0	<5	<5	<5	0	<5	0	<5	0	0	0	0	0	0	0	0	<5	0	<5	<5	<5
Otolaryngology ST3	29	<5	<5	9	14	<5	0	0	5	<5	<5	<5	47	<5	15	12	10	0	5	<5	<5	<5	0	0	0	0	0	0	<5	18	0	<5	<5	<5
Paediatric and Perinatal Pathology ST3	0	<5	0	0	<5	0	0	0	0	0	0	0	0	0	0	0	0	0	0	0	0	<5	0	0	0	0	0	0	0	0	0	0	0	0
Paediatric Cardiology ST4	0	<5	<5	<5	0	<5	0	0	<5	<5	0	0	8	0	6	8	5	0	0	0	<5	0	0	0	0	0	<5	0	0	<5	0	0	<5	<5
Paediatric Surgery ST3	<5	7	<5	<5	0	<5	0	0	<5	<5	0	6	25	<5	6	13	5	0	<5	0	0	0	0	0	0	0	0	0	0	5	0	<5	<5	<5
Paediatrics ST1	6	107	36	34	52	82	<5	10	10	31	0	8	311	22	69	44	62	<5	52	9	17	18	25	<5	<5	7	8	0	5	203	14	23	15	19
Paediatrics ST3	<5	51	22	<5	6	30	<5	8	5	28	0	5	10	<5	9	23	17	0	11	<5	0	0	<5	0	0	0	<5	0	0	<5	<5	<5	0	0
Paediatrics ST4	<5	49	13	<5	5	20	0	5	8	20	0	<5	7	<5	8	23	9	0	9	<5	<5	<5	<5	0	0	<5	<5	0	<5	<5	0	<5	<5	<5
Palliative Medicine ST3	<5	10	6	6	7	5	<5	0	<5	<5	0	0	49	<5	<5	<5	<5	0	<5	<5	<5	0	0	0	0	0	0	0	0	27	<5	0	0	0
Plastic Surgery ST3	0	24	7	16	11	6	<5	<5	<5	<5	<5	5	60	<5	24	13	33	0	<5	<5	6	5	<5	0	0	0	<5	<5	<5	28	<5	8	<5	9
Public Health Medicine ST1	12	90	36	13	33	105	12	6	19	20	5	13	440	35	55	38	56	0	<5	<5	<5	<5	<5	0	0	<5	0	0	<5	68	<5	5	<5	<5
Rehabilitation Medicine ST3	<5	<5	13	<5	5	13	0	<5	<5	6	0	<5	9	<5	<5	5	9	0	<5	<5	0	0	<5	0	<5	0	<5	0	<5	0	0	<5	<5	<5
Sport and Exercise Medicine ST3	0	5	6	0	<5	<5	0	0	<5	0	0	0	11	<5	<5	<5	<5	0	<5	<5	0	<5	0	0	0	0	0	0	0	0	<5	0	0	0
Trauma and Orthopaedic Surgery ST3	<5	113	44	12	23	31	<5	<5	8	21	<5	11	177	7	41	45	58	<5	17	12	6	6	<5	<5	0	0	<5	<5	<5	98	<5	8	5	12
Urology ST3	6	22	13	10	9	18	<5	<5	<5	<5	0	<5	59	8	12	20	12	<5	9	<5	0	<5	<5	<5	<5	<5	0	0	0	23	<5	<5	<5	<5

For peer review only

STROBE Statement—checklist of items that should be included in reports of observational studies

	Item No.	Recommendation	Page No.
**Title and abstract**	1	(*a*) Indicate the study's design with a commonly used term in the title or the abstract	2
		(*b*) Provide in the abstract an informative and balanced summary of what was done and what was found	2
		**Introduction**	
Background/rationale	2	Explain the scientific background and rationale for the investigation being reported	3-5
Objectives	3	State specific objectives, including any prespecified hypotheses	4-5
		**Methods**	
Study design	4	Present key elements of study design early in the paper	5-7
Setting	5	Describe the setting, locations, and relevant dates, including periods of recruitment, exposure, follow-up, and data collection	5-6
Participants	6	(*a*) *Cohort study*—Give the eligibility criteria, and the sources and methods of selection of participants. Describe methods of follow-up<br>*Case-control study*—Give the eligibility criteria, and the sources and methods of case ascertainment and control selection. Give the rationale for the choice of cases and controls<br>*Cross-sectional study*—Give the eligibility criteria, and the sources and methods of selection of participants	5-6
		(*b*) *Cohort study*—For matched studies, give matching criteria and number of exposed and unexposed<br>*Case-control study*—For matched studies, give matching criteria and the number of controls per case	NA
Variables	7	Clearly define all outcomes, exposures, predictors, potential confounders, and effect modifiers. Give diagnostic criteria, if applicable	5-7
Data sources/ measurement	8*	For each variable of interest, give sources of data and details of methods of assessment (measurement). Describe comparability of assessment methods if there is more than one group	5-7
Bias	9	Describe any efforts to address potential sources of bias	5-7
Study size	10	Explain how the study size was arrived at	6

Continued on next page

For peer review only - http://bmjopen.bmj.com/site/about/guidelines.xhtml

For peer review only

Quantitative variables	11	Explain how quantitative variables were handled in the analyses. If applicable, describe which groupings were chosen and why	5-7	
Statistical methods	12	(*a*) Describe all statistical methods, including those used to control for confounding	6-7	
		(*b*) Describe any methods used to examine subgroups and interactions	6-7	
		(*c*) Explain how missing data were addressed	5-7	
		(*d*) *Cohort study*—If applicable, explain how loss to follow-up was addressed<br>*Case-control study*—If applicable, explain how matching of cases and controls was addressed<br>*Cross-sectional study*—If applicable, describe analytical methods taking account of sampling strategy	NA	
		(*e*) Describe any sensitivity analyses	NA	
**Results**				
Participants	13*	(a) Report numbers of individuals at each stage of study—eg numbers potentially eligible, examined for eligibility, confirmed eligible, included in the study, completing follow-up, and analysed	7-13	
		(b) Give reasons for non-participation at each stage	NA	
		(c) Consider use of a flow diagram	NA	
Descriptive data	14*	(a) Give characteristics of study participants (eg demographic, clinical, social) and information on exposures and potential confounders	7-9	
		(b) Indicate number of participants with missing data for each variable of interest	7-9	
		(c) *Cohort study*—Summarise follow-up time (eg, average and total amount)	NA	
Outcome data	15*	*Cohort study*—Report numbers of outcome events or summary measures over time	7-9	
		Case-control study—Report numbers in each exposure category, or summary measures of exposure		
		Cross-sectional study—Report numbers of outcome events or summary measures		
Main results	16	(*a*) Give unadjusted estimates and, if applicable, confounder-adjusted estimates and their precision (eg, 95% confidence interval). Make clear which confounders were adjusted for and why they were included	7-9	country of qualification (UK versus Non-UK) is included as a covariate
		(*b*) Report category boundaries when continuous variables were categorized	NA	
		(*c*) If relevant, consider translating estimates of relative risk into absolute risk for a meaningful time period	NA	

Continued on next page

For peer review only

Other analyses	17	Report other analyses done—eg analyses of subgroups and interactions, and sensitivity analyses	7-9
**Discussion**			
Key results	18	Summarise key results with reference to study objectives	9-10
Limitations	19	Discuss limitations of the study, taking into account sources of potential bias or imprecision. Discuss both direction and magnitude of any potential bias	10-11
Interpretation	20	Give a cautious overall interpretation of results considering objectives, limitations, multiplicity of analyses, results from similar studies, and other relevant evidence	9-15
Generalisability	21	Discuss the generalisability (external validity) of the study results	9-15
**Other information**			
Funding	22	Give the source of funding and the role of the funders for the present study and, if applicable, for the original study on which the present article is based	17

*Give information separately for cases and controls in case-control studies and, if applicable, for exposed and unexposed groups in cohort and cross-sectional studies.

Note: An Explanation and Elaboration article discusses each checklist item and gives methodological background and published examples of transparent reporting. The STROBE checklist is best used in conjunction with this article (freely available on the Web sites of PLoS Medicine at http://www.plosmedicine.org/, Annals of Internal Medicine at http://www.annals.org/, and Epidemiology at http://www.epidem.com/). Information on the STROBE Initiative is available at www.strobe-statement.org.

For peer review only

For peer review only - http://bmjopen.bmj.com/site/about/guidelines.xhtml

BMJ Open

Applications to medical and surgical specialist training in the UK National Health Service, 2021-22: a cross-sectional observational study to characterise the diversity of successful applicants

Journal:	*BMJ Open*
Manuscript ID	bmjopen-2022-069846.R1
Article Type:	Original research
Date Submitted by the Author:	03-Feb-2023
Complete List of Authors:	Aggarwal, Dinesh; University of Cambridge, Department of Medicine; Wellcome Sanger Institute<br>Roy-Chowdhury, Meera; Bar Standards Board<br>Xiang, Nicola; Imperial College Healthcare NHS Trust<br>Peacock, Sharon; Univ Cambridge
**Primary Subject Heading**:	Health services research
Secondary Subject Heading:	Health policy, Medical management, Health services research, Medical education and training
Keywords:	Health policy < HEALTH SERVICES ADMINISTRATION & MANAGEMENT, Organisational development < HEALTH SERVICES ADMINISTRATION & MANAGEMENT, SURGERY, GENERAL MEDICINE (see Internal Medicine), Health Equity, EDUCATION & TRAINING (see Medical Education & Training)

SCHOLARONE™
Manuscripts

I, the Submitting Author has the right to grant and does grant on behalf of all authors of the Work (as defined in the below author licence), an exclusive licence and/or a non-exclusive licence for contributions from authors who are: i) UK Crown employees; ii) where BMJ has agreed a CC-BY licence shall apply, and/or iii) in accordance with the terms applicable for US Federal Government officers or employees acting as part of their official duties; on a worldwide, perpetual, irrevocable, royalty-free basis to BMJ Publishing Group Ltd ("BMJ") its licensees and where the relevant Journal is co-owned by BMJ to the co-owners of the Journal, to publish the Work in this journal and any other BMJ products and to exploit all rights, as set out in our licence.

The Submitting Author accepts and understands that any supply made under these terms is made by BMJ to the Submitting Author unless you are acting as an employee on behalf of your employer or a postgraduate student of an affiliated institution which is paying any applicable article publishing charge ("APC") for Open Access articles. Where the Submitting Author wishes to make the Work available on an Open Access basis (and intends to pay the relevant APC), the terms of reuse of such Open Access shall be governed by a Creative Commons licence – details of these licences and which Creative Commons licence will apply to this Work are set out in our licence referred to above.

Other than as permitted in any relevant BMJ Author's Self Archiving Policies, I confirm this Work has not been accepted for publication elsewhere, is not being considered for publication elsewhere and does not duplicate material already published. I confirm all authors consent to publication of this Work and authorise the granting of this licence.

For peer review only

Applications to medical and surgical specialist training in the UK National Health Service, 2021-22: a cross-sectional observational study to characterise the diversity of successful applicants

*Dr Dinesh Aggarwal[1,2,3], Ms Meera Roy-Chowdhury[4], Dr Nicola Xiang[5], Prof Sharon J. Peacock[1,2]

Affiliations
1. University of Cambridge, Department of Medicine, Hills Rd, Cambridge, CB2 0QQ
2. Cambridge University Hospital NHS Foundation Trust, Hills Rd, Cambridge, CB2 0QQ
3. Wellcome Sanger Institute, Hinxton, Saffron Walden, CB10 1RQ, UK
4. The Bar Standards Board, High Holborn, London, WC1V 7HZ
5. St Mary's Hospital, Imperial College Healthcare NHS Trust, Praed St, London, W2 1NY

* corresponding authors:
Dr Dinesh Aggarwal, University of Cambridge, Department of Medicine, Cambridge, UK. Email: dinesh.aggarwal@nhs.net. Telephone: +441223 336 849

Declarations of Interest
None

For peer review only

Abstract

Objectives: To compare success of applicants to Specialty Training Posts in the UK by gender, ethnicity, and disability status.

Design: Cross-sectional observational study.

Setting: National Health Service, UK.

Participants: All Specialty Training Post applications to Health Education England, UK, during the 2021 to 2022 recruitment cycle.

Main outcome measures: Comparison of success at application to Specialty Training Posts by gender, ethnicity, country of qualification (UK vs Non-UK), and disability. The influence of ethnicity on success was investigated using a logistic regression model, where country of qualification was included as a covariate.

Results: 12419/37971 (32.7%) of applicants to Specialty Training Posts were successful, representing 58 specialities. The difference in percentage of successful females (6480/17523, 37.0%) and males (5625/19340, 29.1%) was 7.9% (95% C.I=6.93-8.86%), in favour of females. Segregation of applications to specialities by gender was observed; surgical specialities had the highest proportion of male applicants, whilst obstetrics and gynaecology had the highest proportion of female applicants. The proportion of successful recruits to specialities largely reflected the number of applications. 11/15 minority ethnic groups (excluding 'Not stated') had significantly lower adjusted odds-ratios for success compared to White-British applicants. 'Mixed White and Black African' (OR=0.52, 95% C.I=0.44-0.61. *p*=<0.001) were least successful minority group in our study, whilst Non-UK graduates had an adjusted odds-ratio for success of 0.43 (95% C.I=0.41-0.46. *p*=<0.001) compared to UK graduates. The difference in percentage of success by disabled applicants (179/464, 38.6%) and non-disabled applicants (11940/36418, 32.8%) was 5.79% (95% C.I=1.23-10.4%), in favour of disabled applicants. No disabled applicants were accepted to 21/58 (36.2%) of specialities.

Conclusions: Despite greater success by female applicants overall, there is an attraction issue to specialities by gender. Further, most ethnic minority groups are less successful at application when compared to White-British applicants. This requires continuous monitoring and evaluation of the reasons behind observed differences.

Strengths and limitations of this study

- This study utilises a large sample of applicants to a national recruitment portal allowing the estimation of success at application to specialist recruitment posts by demographic groups.

For peer review only

- We evaluate three important protected characteristics - gender, ethnicity, and disability - representing the most comprehensive study of the inclusivity of the national recruitment process, to our knowledge.
- By including country of qualification as a covariate in our analysis we account for a key confounding variable.
- Due to Information Commission Office standards, small specialities are largely excluded from the individual specialty level analysis and intersectional analysis beyond what is presented is not possible.
- Due to limitations on data availability longitudinal effects and those of residual confounding factors, such as the influence of socioeconomic background on the disparate success observed by ethnic minority groups, cannot be ascertained here but are important to study in future work.

Introduction

It is crucial for the National Health Service (NHS) to reflect the society which it serves and to nurture diverse talent effectively. Harnessing diverse lived experiences and perspectives strengthens the pool of knowledge and skills within the profession. Diverse teams are more efficient, innovative and make better decisions, meaning that they are ultimately better placed to serve patients (1). Importantly, a lack of workforce diversity can be detrimental to patient care, with research demonstrating inherent biases influencing how clinicians treat patients (2). The NHS is under-staffed at all levels of seniority; backlogs created by the COVID-19 crisis, systemic under-funding, the increasingly complex care needs of an ageing population, and issues with retention of its medical workforce are only serving to exacerbate this (3). The UK is reported to employ 3.03 doctors per 1000 people compared to an average of 3.7 per 1000 people in the Organisation for Economic Co-operation and Development (OECD) European Union nations (4). These issues are likely to only worsen as the workforce supply and demand gap is predicted to widen (5). Creating a more inclusive culture in the NHS is required

For peer review only - http://bmjopen.bmj.com/site/about/guidelines.xhtml

to ensure that a wider, more diverse talent pool is attracted, able to break into the profession, able to progress successfully, and retained.

Disparate representation within the NHS workforce is well reported (6-8). Surgical specialities have the lowest proportion of female consultants, and gender parity within surgical specialities is predicted to be reached in decades rather than years (8). Such disparities have inevitable knock-on effects. A lack of female representation contributes towards a male dominated culture, and results in fewer female role models to inspire and encourage aspiring female doctors. These are known factors in deterring female applicants as early as undergraduate training (9). A recent independent report examining the gender pay gap in medicine in England found that the mean whole-time equivalent pay gap is 18.9% for hospital doctors, 15.3% for GPs and 11.5% for clinical academics in favour of men; a key recommendation to reduce gender pay was to minimise gender segregation within specialties (6). Additionally, 41.9% of the NHS medical workforce comprises individuals from minority ethnic groups, yet only 22.7% at Clinical Director level (7). Potential bias against minority ethnic groups embedded in recruitment processes and decisions has been demonstrated in multiple sectors and can occur at multiple stages (10-12). A historical study from Esmail et al reported poorer outcomes for matched applicants to specialty medicine with Asian names compared to those with English names (10). More recent data has highlighted differences in applicants deemed appointable for specialty posts by ethnicity, but the data failed to account for the impact of country of graduation – a known additional factor in success at postgraduate examinations and recruitment (13, 14).

For peer review only

As the largest employer in the UK, the NHS must have fair and transparent recruitment processes. This responsibility is reinforced by the fact that the NHS is governed by the Public Sector Equality Duty with the stated objectives to: “eliminate unlawful discrimination; advance equality of opportunity between people who share a protected characteristic and those who don’t; foster or encourage good relations between people who share a protected characteristic and those who don’t” (15). Systematic examination of disparities in recruitment data by protected characteristics across specialties is therefore a key missing piece of analysis.

Investment of time and resources in investigating any disparities highlighted, addressing the root causes through action and mitigation, and evaluating and measuring progress over time will help to progress diverse representation throughout all levels of seniority, and create a more inclusive culture across the NHS clinical workforce. Here, we examine disparities in the outcomes for the recruitment to all Specialty Training Posts in the UK in 2021 by gender, ethnicity, and disability status.

Methods

Specialty Training Post recruitment process

Obtaining a national training number for specialty training is the route which generates the vast majority of new consultants and general practitioners in the UK. Depending on the specialty, length of training can be variable and entry points for candidates can also vary (i.e. some post-graduate junior doctors may enter specialty training in the first year of the respective Specialty Training Programme or may be deemed to have the competencies required to apply at a later stage in the Programme; these entry points are donated by a prefix of 'ST' or 'CT' followed the year of training at which the Specialty Training Posts is allowing entry. Applications are made online and mostly processed/organised by a lead Health Education England (HEE) local office/devolved nation in order to prevent the need for multiple applications to access posts in different geographical locations. Briefly, the process involves:

- shortlisting of candidates which can include self-assessment or a Multi-Specialty Recruitment Assessment
- an interview, recommended to be multi-panel
- ranking based on shortlisting and interview scores
- offer of appointment.

Demographic details are self-reported. Options for gender include 'Male', 'Female', and 'Not Stated'. Options for ethnicity include 'White - British ', 'Asian or Asian British - Bangladeshi',

For peer review only - http://bmjopen.bmj.com/site/about/guidelines.xhtml

'Asian or Asian British - Indian', 'Asian or Asian British - Pakistani', 'Chinese', 'Any other Asian background', 'Black or Black British - African', 'Black or Black British - Caribbean', 'Any other Black background', 'Mixed White and Asian', 'Mixed White and Black African', 'Mixed White and Black Caribbean', 'Any other Mixed background', 'White - Irish', 'Any other White background', 'Any other ethnic background', and 'Not stated'. Options for Disability include 'Yes' (i.e. applicant has a disability), 'No', and 'Not stated', and data pertaining to the category of disability was not available for analysis. Further details on the application process for individual specialties in the UK can be found here, https://specialtytraining.hee.nhs.uk/Recruitment (16).

Study participants

Data on the gender, ethnicity, disability status, and country of qualification (UK vs Non-UK) of applicants to Specialty Training Posts for the 2021-22 recruitment cycle was made available through two separate Freedom of Information (FOI) requests to HEE. HEE follows the Information Commissioner's Office code of practice relating to data anonymisation; release of small numbers (<5 in a geographical area/demographic) has therefore been suppressed. All data made available through the FOI request, including specialities where data is incomplete due to low numbers, can be found in supplementary files (**Supplementary Data**).

Statistical analysis

Analysis was performed in Excel 2016 and R version 4.02. Figures were generated in R version 4.2.1. Probabilities were calculated from estimated models using the emmeans package (1.7.3) (17). Where individual specialities have been grouped for graphical representation, the breakdown of these groups (e.g. surgical) can be found in **Supplementary Table 1**. Differences in percentages of successful recruitment between categories are reported with 95% confidence intervals derived using the prop.test function in R. Where 95% confidence intervals do not cross the line of no-effect (i.e. zero in the case of differences in

For peer review only - http://bmjopen.bmj.com/site/about/guidelines.xhtml

percentages of successful recruitment between categories), the result is statistically significant. When assessing completeness of data to consider the difference in percentages of successful applicants by gender, data relating to applicants with undisclosed gender are excluded. The influence of ethnicity on success at application to specialty posts is investigated using a logistic regression model by univariable and multivariable analysis (outcome ~ graduate status + ethnic origin) where country of qualification (UK versus Non-UK) is included as a covariate. Variation of success at application between ethnic groups by country of qualification is then examined by including interaction terms (outcome ~ graduate status + ethnic origin + graduate status:ethnic origin) between the two variables. This study complies with the STROBE reporting guidelines for observational studies in Epidemiology (18).

Patient and Public Involvement

Patients or the public were not involved in study design, data collection and analysis, decision to publish, or preparation of the manuscript.

Results

There were 12419/37971 (32.7%) successful applicants to Health Education England for Training Posts representing 58 specialty posts in 2021; a distribution of applications by specialty is shown in **Supplementary Table 2**.

Recruitment by gender

When considering all applicants, we found 17523/37971 (46.1%) were female, and 1108/37971 (2.9%) individuals preferred not to state their gender. Of the successful applicants, 6480/12419 (52.2%) were female and 5625/12419 (45.3%) were male. Overall, the difference in percentage of success by females (6480/17523, 37.0%) and males

For peer review only - http://bmjopen.bmj.com/site/about/guidelines.xhtml

(5625/19340, 29.1%) was 7.9% (95% C.I.= 6.93-8.86%), in favour of females **(Figure 1, Supplementary Figure 1**).

Recruitment to Specialities by gender

Complete data representing male and female applications was available for 56/58 (96.6%) Specialty Training Posts, whilst 40/58 (69.0%) Specialty Training Posts had complete data representing successful male and female applications. When considering applications by individual specialty, 13/40 (32.5%) specialty posts had a higher percentage of successful females when compared to males, compared to specialty 2/40 (5.0%) posts that had a higher percentage of successful males (**Figure 1**). Despite this, 22/40 (55.0%) specialty posts had a greater absolute number of successful male applicants compared to females, representing an 'attraction' issue. When examining absolute numbers of applicants and successful applicants by gender, there was an observed segregation by specialty (**Figure 2, Supplementary Figures 2 and 3**). Of note, surgical specialities (3097/4742, 65.3%) and radiology (1146/1783, 64.3%) had the highest proportion of male applicants, whilst obstetrics and gynaecology (957/1321, 72.4%) and public health (644/959, 67.2%) had the highest proportion of female applicants. The gender balance of successful recruits to specialities largely reflected that of the pool of applicants (**Figure 2c**).

Recruitment by Ethnicity and Country of Qualification

Applications for Specialty Training Posts by ethnicity are shown in **Figure 3**. In the 2021-22 application cycle, 19044/37971 (50.2%) of applicants to Specialty Training Posts were Non-UK medical graduates **(Supplementary Figure 4)**. Excluding non-medical applicants to Public Health Training Posts, of the successful applicants, 4334/12419 (34.9%) were Non-UK graduates and 7987/12419 (64.3%) were UK graduates. Overall, the difference in percentage of success by UK graduates (7987/17939, 44.5%) and Non-UK graduates (4334/19044, 22.8%) was 21.8% (95% C.I. = 20.8-22.7%), in favour of UK graduates (**Supplementary**

For peer review only - http://bmjopen.bmj.com/site/about/guidelines.xhtml

Table 4). UK graduates were significantly more successful in all ethnic groups other than 'Chinese' and 'Any other Black background' (**Supplementary Table 4**).

Using a multivariable logistic regression analysis, including country of qualification as a covariate, we found 11/15 (73.3%) minority ethnic groups (excluding 'Not stated') to have a significantly lower odds ratio of success at recruitment when compared to White-British applicants (**Figure 4 and Supplementary Table 5**). Non-UK graduates had a significantly lower odds ratio of success at recruitment when compared to UK-graduates (adjusted OR=0.43, 95% C.I = 0.41 to 0.46. *p* = <0.001). All adjusted and unadjusted odd-ratios for success by country of qualification and minority ethnic group are provided in **Supplementary Tables 5** and **6**. Additionally, only the 'Asian or Asian British – Bangladeshi' group significantly differed in outcome across the graduate status (p=0.02) in the interaction model. (**Supplementary Table 7 and Supplementary Figure 5**).

Recruitment by Disability

When considering all applicants to Specialty Training Posts, we found 464/37971 (1.2%) were disabled, and 1089/37971 (2.9%) individuals preferred not to state their disability status. Of the successful applicants, 179/12419 (1.4%) were disabled and 11940/12419 (96.1%) were non-disabled. Overall, the difference in percentage of success by disabled applicants (179/464, 38.6%) and non-disabled applicants (11940/36418, 32.8%) was 5.79% (95% C.I. = 1.23-10.4%), in favour of disabled applicants (**Supplementary Figure 6**). However, there were no disabled applicants to 13/58 (22.4%) specialities and a further 21/58 (36.2%) specialities where no disabled applicants were accepted. Of the specialities where data was available, General Psychiatry ST4 had the highest acceptance rate for disabled applicants (15/16, 93.8%), whilst General Practice had the highest absolute number of successful applicants (61/121, 50.4%).

Discussion

For peer review only

We present the most complete assessment of diversity data relating to recruitment to Specialty Training Posts in the UK to our knowledge. Overall, of the specialties studied, female applicants were more successful in the recruitment process though we note clear segregation of applications by specialty. We observe a low absolute number of successful female applicants to Surgical specialities, Radiology, and Ophthalmology, and a low absolute number of successful male applicants to Obstetrics and Gynaecology, Public Health and Paediatric Medicine. We show that several minority ethnic groups are less likely to be successful in their applications when adjusting for country of graduation. Finally, we find disabled applicants to be more successful in the recruitment process when compared to non-disabled applicants, but again with significant variation in applications and success at individual specialty level. The NHS has committed to fostering a truly inclusive environment; this work, and the associated actions (**Table 1**), should serve as a framework to evaluate recruitment data, and subsequently review policies and processes to enable the implementation of improvements, to ensure that this commitment can be achieved.

For peer review only

Strengths and limitations of this study, and future work

We use a large sample of applicants to a national recruitment portal maintained by HEE which allows us to estimate the success at application to specialist recruitment posts by demographic groups. We evaluate three important protected characteristics, gender, ethnicity, and disability, which represents the most comprehensive evaluation of the inclusivity of the national recruitment process to our knowledge. By including country of qualification as a covariate in our analysis, we account for a key confounding variable, demonstrating unequal success by ethnicity despite country of qualification; an important result that suggests the recruitment process needs further investigation.

This study has a number of limitations. Due to Information Commission Office standards, small specialities are largely excluded from the individual specialty level analysis. Data is particularly incomplete at individual specialty level for ethnicity and disability data. Further, a comparative

analysis by specialty is limited by the small numbers in each specialty and beyond the scope of this study. Due to the nature of the data, multivariable analysis beyond what is presented is not possible; an intersectional analysis of protected characteristics, e.g. females from ethnic minority backgrounds, should be conducted to identify groups that may be particularly disadvantaged. Further, residual confounding factors, such as the influence of socioeconomic background on the disparate success observed by ethnic minority groups cannot be ascertained here but present an important factor to study in future work. The data is representative of one year of recruitment, and possible enduring effects of the COVID-19 pandemic on applications and recruitment patterns cannot be disregarded; ongoing systematic data monitoring is necessary to evaluate trends in applications and successful recruitment by demographic characteristics over time. The data was obtained through two separate FOI requests; one representing aggregated data for 'all Specialty Training Posts' and the second including data separated by individual specialties. Data analysis does not include comparison of the aggregated data between datasets but we do note a minor difference in the total number of applicants between datasets (raw data is provided in supplementary files) – given the large sample size, these are not expected to influence our findings. We report an analysis of recruitment to Specialty Training Posts which is only one aspect of assessing the diversity and inclusiveness of a profession; additional work should assess how these findings relate to pay gaps, progression and retention of the workforce, and for any barriers faced by groups representing other protected characteristics as defined by the Equality Act 2010 within the NHS. We note 'Black or Black British – African', 'Black or Black British – Caribbean', and 'Mixed White and Black Caribbean' applicants do not have a lower odds ratio of success at application when compared to White British applicants; this does not mean the absolute numbers of applicants are not disproportionately lower or that this group are not disadvantaged at other stages of their career progression. Finally, to reiterate, the NHS workforce should reflect the population it serves at all levels. This extends beyond clinicians – similar work should be undertaken for allied health professionals and support staff in the NHS.

Implications and supporting evidence

Over-representation of males in senior positions of medicine in the NHS, and in particular specialities such as surgery, has been noted previously (19) but national data on recruitment across specialty posts is limited. Success by female applicants in many specialties, demonstrated in our study, is a positive step towards gender balance, and perhaps reflects existing efforts to address disparities, or higher applicant quality where female applications are low. Additionally, data provided by NHS Digital demonstrates a steady rise in the proportion of female specialty trainees, from 45% in 2009 to 53% in 2017, suggesting either an increase in female applicants or successes, or a combination (19). This does not however provide clarity on the distribution of female specialist trainees across specialties. The skew in applications and subsequent recruitment by gender, particularly amongst surgical specialities, is concerning. There is a clear attraction issue (i.e. a lack of diversity amongst applicants) amongst multiple specialities (**Figure 2a and Supplementary Figure 1**), with several possible reasons. Taking surgery as an example, factors including a male-dominated workplace culture, incidents of bullying and harassment, few female role models, and career inflexibility, have been suggested as the reasons why females are deterred from considering to apply, as early as undergraduate study (9, 20). Female surgeons have reported quality of life and fewer unsocial hours as explanations of why women prefer other clinical specialities, in addition to the fear that working less-than-full-time (LTFT) or taking career breaks is perceived negatively (20). There are exceptions to this where some specialities appear to have succeeded in creating a culture that attracts female trainees and allows them to thrive. Obstetrics and Gynaecology provides a clear example of a demanding specialty requiring shift work and a surgical skill set (21) where females dominate applications and successful hires. Obstetrics and Gynaecology, Public Health, and Paediatric medicine, are known to have a higher representation of females compared to males (6), and continue to disproportionately recruit females; these represent 3 or the 4 specialities with the highest proportion of LTFT trainees (22). The availability of LTFT opportunities compound gender segregation within specialities,

For peer review only

and can exacerbate the gender pay gap (6). Additional specialty-specific factors will inevitably influence a doctor's application decision-making, including personal choice and even patient preference (studies have demonstrated a slight preference for female Obstetric and Gynaecology clinicians by their female patient cohort, for example (23)), and these should be taken into consideration. However, the aim should be to ensure that doctors are given the opportunity to flourish in all specialities and that this is facilitated by fair recruitment.

Concerningly, we find disparities in the success of applicants by ethnicity and this is not explained by country of graduation alone. A previous descriptive analysis demonstrated that doctors from minority ethnic groups were less likely to be considered suitable for appointment to Specialty Training Posts in the UK, but did not consider country of graduation or undertake a thorough analysis of the data (24). Examining Combined Infection Training specifically, country of qualification possibly accounted for imbalances in recruitment by ethnicity (13). Our findings, however, indicate a need to thoroughly review recruitment policies and processes from a diversity and inclusion perspective (such as bias in recruitment decisions and a lack of inclusivity in the design of recruitment processes), to ensure that they are facilitating equitable outcomes. Outside of recruitment to specialty training, unequal progression by ethnicity has been demonstrated in the NHS. Doctors from minority ethnic groups are underrepresented at consultant grade and within academic roles (7). They have reported disproportionately high levels of discrimination from work colleagues, and had poorer outcomes at revalidation and Annual Review of Competence Progression, regardless of country of qualification. Additionally, differences in postgraduate examination pass rates by country of qualification, and between minority ethnic groups are established (14). These differences can be overcome; data from NHS Digital demonstrates an incremental rise in the proportion of consultant grade doctors from minority ethnic groups over time, from 29% in 2009 to 36% in 2017 (19), whilst the NHS WRES demonstrated that in regions where a concerted effort to improve disparate outcomes for ethnic minority employees had been made, significant improvements were seen (25). We should be mindful that unequal outcomes may reflect a history of disparities in the

For peer review only - http://bmjopen.bmj.com/site/about/guidelines.xhtml

opportunities available to, and experiences of, various demographic groups and we encourage the NHS to support access schemes that aim to help doctors at an early-career stage. The 'widening access' initiative introduced by HEE, for example, is a positive step in attempting to support the applications of Non-UK graduate doctors to Specialty Training Posts (26); schemes such as these should be maintained after HEE merges with NHS England. The fragmented nature of the NHS and its complex employment structures mean monitoring progression and attainment by protected characteristics is perhaps more complex than other professions, but effort must be made to formulate a consistent methodology to do so. The NHS is facing a workforce crisis (3) and doctors from ethnic minority backgrounds are being increasingly relied on in the UK. They comprise 41.9% (53,157) of the medical and dental workforce in NHS trusts and clinical commissioning groups in England in 2020 (7); therefore, ensuring that these doctors are able to work within an inclusive environment, that allows them to thrive and progress should be a priority.

Only 1.2% of the applicants to Specialty Training Posts reported a disability – this will likely be an under-estimate with under-reporting of disabilities amongst medical professionals (27). It is encouraging, however, to observe a high proportion of acceptances amongst all individuals disclosing a disability, with a particularly high proportion of success seen in Psychiatry and Paediatric Medicine. The 2021 Workforce Disability Equality Standard found 23.4% of the disabled staff working in the NHS did not have the adjustments necessary to effectively work in the NHS (27). Ultimately, the NHS needs to ensure that application and recruitment processes are accessible and open to adjustments for all disabilities (including neurodiversity), eliminate any perceived fear of discrimination, and provide assurance that all NHS workplaces will accommodate reasonable adjustments to ensure that disabled doctors can carry out their work. This will not only help to encourage more disabled applicants, but also make disabled clinicians to feel more comfortable disclosing this information, in turn support the monitoring and evaluation of diversity data. Additionally, given the diversity of issues faced by disabled doctors, we encourage assessment of this protected characteristic by taking the nature of

For peer review only - http://bmjopen.bmj.com/site/about/guidelines.xhtml

one's disability into account. Stakeholders should engage with disabled doctors through networks such as the Disability Doctors Network, to identify possible barriers to disclosing their disability, applying to become, and working as a doctor, and how these could be overcome. Disabled doctors harbour unique insights and provide an opportunity to relate to the patient journey (28); the NHS would stand to benefit from this expertise if they are supported to prosper within its environment.

Conclusions

Overall, we present detailed analysis of the diversity and inclusion considerations for recruitment to specialty training in the UK for the 2021-22 cycle. This study provides evidence to demonstrate higher success rates by females compared to men but gender segregation of applications by specialty. Our data highlights poorer outcomes for minority ethnic applicants, regardless of country of graduation. We show favourable outcomes for disabled doctors and encourage data reporting in this domain to be improved. Examination of recruitment processes is the first step towards building an inclusive workplace; we encourage policy makers to investigate the root cause of our findings and ensure such monitoring is made routine.

Recruitment specific recommendation
Processes and data pertaining to the allocation of Specialty Training Posts should be transparent and published.
Ensure that recruitment panels are as diverse as possible.
Ensure that all staff involved in recruitment processes are trained in fair and inclusive recruitment, with regular refresher training.
Language used in applications should be inclusive and examined to ensure it does not deter any demographic group.
Review job criteria to ensure that only "essential" skills and experience is included. Women for example, are less likely to apply for a job where they do not meet all the criteria.
Include a self-identification category for gender.
Recruitment systems and processes should be flexible to accommodate for reasonable adjustments. This should be plainly messaged to applicants, along with a clearly signposted process for requesting reasonable adjustments.
Review application tests for inclusivity and fairness across protected characteristics.
Implement positive action schemes to support applicants from groups with disproportionately low success rates.
Wider approaches to promote inclusivity to address attraction issues

For peer review only - http://bmjopen.bmj.com/site/about/guidelines.xhtml

Support all genders with less-than-full-time applications.
Actively support woman returning from maternity leave to increase confidence and mitigate de-skilling (such as return to work transition programmes and 'keeping-in-touch' days).
De-stigmatise parental leave for all genders.
Encourage male applications to traditionally female dominated specialities to help break-down existing gender stereotypes within the NHS.
Promote diversity in specialities that are under-represented by certain demographic groups by highlighting role models and positive messaging.
Investigate cultural issues within specialties where this is a problem; carry out engagement surveys and focus groups to determine how these can be addressed.
Create and promote a culture with zero tolerance for bullying and harassment, and ensure that doctors feel safe to speak out.
Create outreach programmes for those specialties with attraction issues for certain groups.
Ensure workplaces are accessible and processes for reasonable adjustments (including successful examples of reasonable adjustments) are well advertised.
Engage with employee networks and patient groups to understand specific issues faced by different demographic groups and promote Equality, Diversity, and Inclusion related activities.

Table 1: Actions for consideration to improve inclusivity in the Specialty Post Recruitment process

Acknowledgements

We thank members of the Freedom of Information office at Health Education England for providing data. We thank Mr Vivek Roy Chowdhury, Department of Economics, University of Cambridge, and Dr Oliver Feng, Department of Mathematics, University of Cambridge, for providing direction on statistical analysis.

Ethics approval

This study uses data obtained from a freedom of information request, that is routinely collected and obtained in line with the Information Commissioner's Office. Ethical approval is therefore not required.

Transparency statement

Author DA (manuscript guarantor) affirms the manuscript is an honest, accurate, and transparent account of the study being reported; that no important aspects of the study have

For peer review only

For peer review only - http://bmjopen.bmj.com/site/about/guidelines.xhtml

been omitted; and that any discrepancies from the study as originally planned (and, if relevant, registered) have been explained.

Competing interests statement

None declared.

Data statement

All data relevant to the study are included in the article or uploaded as supplementary information.

For peer review only

Contributions

Conceptualisation: D.A, M.R.C; Project administration: D.A.; Investigation: D.A.; Data curation: D.A.; Methodology: D.A., M.R.C.; Visualization: D.A.; Formal analysis: D.A.; Writing—original draft preparation: D.A.; Writing—review and editing: D.A, M.R.C, N.X., S.J.P.; Funding acquisition: N/A; Supervision: S.J.P.

Copyright Statement

The corresponding author has the right to grant on behalf of all authors and does grant on behalf of all authors, an exclusive licence on a worldwide basis to the BMJ Publishing Group Ltd to permit this article (if accepted) to be published in BMJ editions and any other BMJPGL products and sublicences such use and exploit all subsidiary rights, as set out in our licence.

Funding Statement

DA is a Clinical PhD Fellow and gratefully supported by the Wellcome Trust (Grant number: 222903/Z/21/Z). This research did not receive any specific grant from funding agencies in the public, commercial, or not-for-profit sectors. This is an open access article distributed in accordance with the terms of the Creative Commons Attribution (CC BY 4.0) license, which permits others to distribute, remix, adapt and build upon this work, for commercial use, provided the original work is properly cited. See: http://creativecommons.org/licenses/by/4.0.

References

1. Gomez LE, Bernet P. Diversity improves performance and outcomes. J Natl Med Assoc. 2019;111(4):383-92.
2. Rotenstein LS, Reede JY, Jena AB. Addressing Workforce Diversity - A Quality-Improvement Framework. N Engl J Med. 2021;384(12):1083-6.
3. Committee HaSC. Workforce: recruitment, training and retention in health and social care: UK Parliament; 2022 [Available from: https://publications.parliament.uk/pa/cm5803/cmselect/cmhealth/115/report.html.
4. Association BM. NHS medical staffing data analysis 2022 [Available from: https://www.bma.org.uk/advice-and-support/nhs-delivery-and-workforce/workforce/nhs-medical-staffing-data-analysis.
5. Shembavnekar N BJ, Bazeer N, Kelly E, Beech J, Charlesworth A, McConkey R, Fisher R. . NHS workforce projections 2022. The Health Foundation. 2022.
6. Jane Dacre CW, Carol Atkinson, Ioannis Laliotis, Mark Williams, Jo Blanden, Sheila Wild, Duncan Brown. Mend the Gap: The Independent Review into Gender Pay Gaps in Medicine in England: Department of Health and Social Care 2020; 2020 [Available from: https://assets.publishing.service.gov.uk/government/uploads/system/uploads/attachment_data/file/944246/Gender_pay_gap_in_medicine_review.pdf.
7. team WI. Medical Workforce Race Equality Standard (MWRES): WRES indicators for the medical workforce 2020: NHS England; 2021 [Available from: https://www.england.nhs.uk/wp-content/uploads/2021/07/MWRES-DIGITAL-2020_FINAL.pdf.
8. Newman TH, Parry MG, Zakeri R, Pegna V, Nagle A, Bhatti F, et al. Gender diversity in UK surgical specialties: a national observational study. BMJ Open. 2022;12(2):e055516.
9. Skinner H, Bhatti F. Women in surgery. The Bulletin of the Royal College of Surgeons of England. 2019;101(suppl_6):12-4.
10. Esmail A, Everington S. Racial discrimination against doctors from ethnic minorities. Bmj. 1993;306(6879):691-2.
11. Wood M, Hales J, Purdon S, Sejersen T, Hayllar O. A test for racial discrimination in recruitment practice in British cities. Department for Work and Pensions Research Report. 2009;607.
12. Adamovic M. When ethnic discrimination in recruitment is likely to occur and how to reduce it: Applying a contingency perspective to review resume studies. Hum Resour Manage R. 2022;32(2).
13. Aggarwal D, Roy-Chowdhury M, Xiang N, Peacock SJ. Equity in recruitment to Combined Infection Training, 2021: Diversity & Inclusion considerations. J Infect. 2022.
14. MRCP(UK) and Specialty Certificate Examinations Pass Rates by Gender and Ethnicity 2021: MRCP(UK); 2021 [Available from: https://www.mrcpuk.org/sites/default/files/documents/Equality%20report%202021.pdf.
15. office Ge. Equality Act 2010: Public Sector Equality Duty. What do I need to know? A quick start guide for public sector organisations.: GOV.UK; 2011 [Available from: https://assets.publishing.service.gov.uk/government/uploads/system/uploads/attachment_data/file/85041/equality-duty.pdf.
16. England HE. Medical Specialty Recruitment 2022: Health Education England; [Available from: https://specialtytraining.hee.nhs.uk/Recruitment.
17. Lenth R, Singmann H, Love J, Buerkner P, Herve M. Emmeans: Estimated marginal means, aka least-squares means. R package version. 2018;1(1):3.
18. Vandenbroucke JP, von Elm E, Altman DG, Gotzsche PC, Mulrow CD, Pocock SJ, et al. Strengthening the Reporting of Observational Studies in Epidemiology (STROBE): explanation and elaboration. Int J Surg. 2014;12(12):1500-24.

For peer review only - http://bmjopen.bmj.com/site/about/guidelines.xhtml

19. Digital N. Analysis of the representation of women across the hospital and community health services workforce: NHS Digital; 2018 [Available from: https://digital.nhs.uk/data-and-information/find-data-and-publications/supplementary-information/2018-supplementary-information-files/analysis-of-the-representation-of-women-across-the-hospital-and-community-health-services-workforce.
20. Bellini MI, Graham Y, Hayes C, Zakeri R, Parks R, Papalois V. A woman's place is in theatre: women's perceptions and experiences of working in surgery from the Association of Surgeons of Great Britain and Ireland women in surgery working group. BMJ Open. 2019;9(1):e024349.
21. Wise J. Life as a physician in obstetrics and gynaecology. BMJ. 2020;368:m37.
22. Council GM. National training survey: General Medical Council; 2022 [Available from: https://www.gmc-uk.org/education/how-we-quality-assure-medical-education-and-training/evidence-data-and-intelligence/national-training-surveys.
23. Janssen SM, Lagro-Janssen AL. Physician's gender, communication style, patient preferences and patient satisfaction in gynecology and obstetrics: a systematic review. Patient Educ Couns. 2012;89(2):221-6.
24. Iacobucci G. Specialty training: ethnic minority doctors' reduced chance of being appointed is "unacceptable". BMJ. 2020;368:m479.
25. England N. NHS Workforce Race Equality Standard: 2021 data analysis report for NHS trusts: NHS England; 2022 [Available from: https://www.england.nhs.uk/wp-content/uploads/2022/04/Workforce-Race-Equality-Standard-report-2021-.pdf.
26. England HE. Widening Access to Specialty Training: start your medical career in the NHS hee.nhs.uk: Health Education England; [Available from: https://specialtytraining.hee.nhs.uk/wideningaccess.
27. Workforce Disability Equality Standard: 2021 data analysis report for NHS trusts and foundation trusts: NHS England; 2022 [Available from: https://www.england.nhs.uk/wp-content/uploads/2022/05/Workforce-Disability-Equality-Standard-2021-data-analysis-report-for-NHS-trusts-and-foundation-trusts.pdf.
28. GMC. Welcomed and valued: Supporting disabled learners in medical education and training GMC: GMC; 2019 [Available from: https://www.gmc-uk.org/education/standards-guidance-and-curricula/guidance/welcomed-and-valued.

Figure Legends

For peer review only

Figure 1: Success of applicants to Training Posts by specialty in the 2021 recruitment year by gender.
Figure showing the applications of trainees to Specialty Training Posts and percentage of successful applicants by gender, where data is complete. All values are provided to 3 significant figures. Where confidence intervals for estimated differences between the percentage of successful applicants by gender does not cross zero, bars are highlighted in orange (greater female success) or purple (greater male success). Specialities marked with ^ contained small numbers (i.e expected frequencies were lower than 5 in any domain of the contingency table) and therefore Fisher-exact test results to examine differences in proportions of successful candidates are also provided in **Supplementary Table 3**.

Figure 2: Applications and outcomes for Specialty Training Posts separated into groups of specialities in the 2021-22 recruitment year by gender.
(a) Proportion of applicants to specialty groups by gender. (b) Number of applicants to specialty groups by gender. (c) Proportion of successful applicants to specialty groups by gender. (d) Number of successful applicants to specialty groups by gender. Groupings of specialties can be found in **Supplementary Table 1**.

Figure 3: Applications and outcomes for Specialty Training Posts in the 2021-2022 recruitment year by ethnicity.
Applications to Specialty Training Posts in the 2021 recruitment year by Ethnicity.

Figure 4: Applications and outcomes for Specialty Training Posts in the 2021-2022 recruitment year by ethnicity and graduate status.
(a) Unadjusted Odds ratios for success by ethnicity when compared to White British applicants derived from a logistic regression model (b) Adjusted Odds ratios for success by ethnicity compared to White-British where country of qualification (UK versus Non-UK) is included as a covariate derived from a multivariable logistic regression model. Non-Medical applicants to Public Health Specialty Training have been removed (n=988). Odds ratios are presented with 95% Confidence Intervals. * *p<0.05* ** *p<0.01* *** *p<0.001*.

For peer review only

Specialty	Total	Male Success (%)	Female Success (%)	Percentage Difference (95% CI)
General Practice ST1	9803	41.80	49.40	7.62 (5.63 to 9.61)
Core Surgical Training CT1	2418	22.30	21.40	−0.99 (−4.45 to 2.47)
ACCS Internal Medicine/Internal Medicine CT1	3495	41.10	46.50	5.41 (2.06 to 8.75)
ACCS Anaesthetics/Core Anaesthetics CT1	2390	23.70	30.70	7.02 (3.32 to 10.70)
Clinical Radiology ST1	1783	19.40	22.90	3.55 (−0.56 to 7.66)
Core Psychiatry CT1	2056	26.80	36.40	9.68 (5.59 to 13.80)
ACCS Emergency Medicine ST1/CT1	1382	20.50	32.30	11.80 (6.94 to 16.70)
Anaesthetics ST3	1590	33.90	37.40	3.55 (−1.36 to 8.47)
Trauma and Orthopaedic Surgery ST3	587	25.20	52.00	26.90 (15.70 to 38.10)
General and Vascular Surgery ST3	595	18.70	27.90	9.22 (1.70 to 16.70)
Ophthalmology ST1	593	13.80	14.30	0.48 (−5.58 to 6.55)
Public Health Medicine ST1	959	10.50	9.78	−0.69 (−5.02 to 3.63)
Intensive Care Medicine ST3	584	32.70	35.90	3.28 (−5.33 to 11.90)
Cardiology ST3	386	23.90	23.50	−0.41 (−10.40 to 9.61)
Obstetrics and Gynaecology ST1	852	15.70	34.70	19.10 (12.80 to 25.40)
Emergency Medicine ST3^	231	6.35	14.30	7.94 (−4.66 to 20.50)
Gastroenterology ST3	250	16.60	19.00	2.47 (−9.66 to 14.60)
Paediatrics ST1	870	42.60	50.50	7.85 (0.47 to 15.20)
Neurosurgery ST1^	232	6.06	7.46	1.40 (−6.92 to 9.72)
Haematology ST3	358	17.80	28.80	11.00 (1.76 to 20.20)
Histopathology ST1	336	23.70	36.70	12.90 (2.65 to 23.20)
Combined Infection Training ST3	266	23.20	28.10	4.94 (−6.32 to 16.20)
General Psychiatry ST4	637	62.70	72.50	9.85 (2.22 to 17.50)
Paediatrics ST3	220	9.65	11.30	1.67 (−7.35 to 10.70)
Urology ST3	193	24.30	22.80	−1.46 (−15.80 to 12.80)
Obstetrics and Gynaecology ST3	309	7.45	6.51	−0.94 (−7.95 to 6.08)
Paediatrics ST4	175	11.80	19.50	7.68 (−4.26 to 19.60)
Plastic Surgery ST3	200	34.50	34.50	−0.03 (−13.30 to 13.30)
Clinical Oncology ST3	257	43.30	40.80	−2.54 (−15.40 to 10.30)
Otolaryngology ST3	152	20.90	21.30	0.43 (−13.20 to 14.10)
Neurology ST3	159	29.90	30.60	0.75 (−14.70 to 16.10)
Cardiothoracic Surgery ST1^	109	8.70	0.00	−8.70 (−17.30 to −0.07)
Medical Oncology ST3	204	33.00	39.80	6.86 (−7.36 to 21.10)
Emergency Medicine ST4	136	36.80	59.20	22.40 (3.72 to 41.10)
Community Sexual and Reproductive Health ST1^	160	0.00	5.00	5.00 (−0.57 to 10.60)
Ophthalmology ST3	80	17.00	21.20	4.19 (−16.00 to 24.40)
Palliative Medicine ST3	101	29.00	35.70	6.68 (−15.20 to 28.50)
Occupational Medicine ST3^	45	23.10	0.00	−23.10 (−43.80 to −2.33)
Sport and Exercise Medicine ST3^	33	23.10	0.00	−23.10 (−48.30 to 2.18)
Chemical Pathology ST3^	29	31.20	38.50	7.21 (−34.60 to 49.00)
All Applicants*	36863	29.10	37.00	7.90 (6.93 to 8.86)

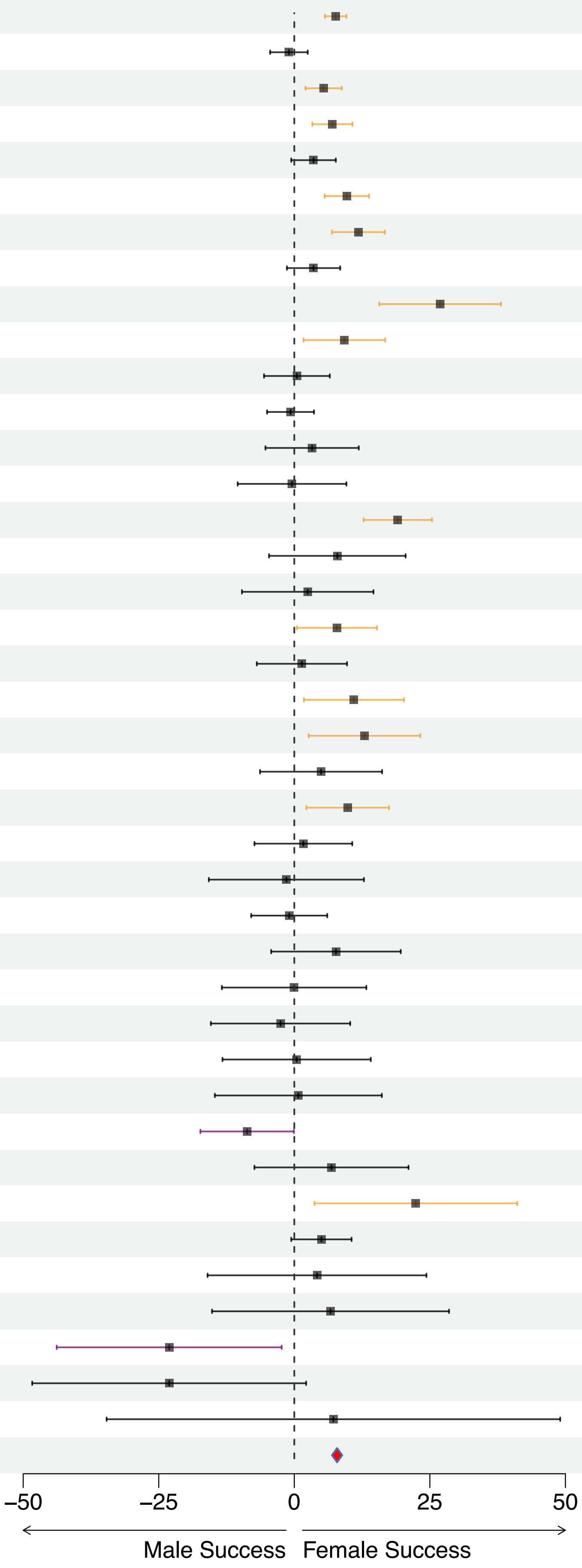

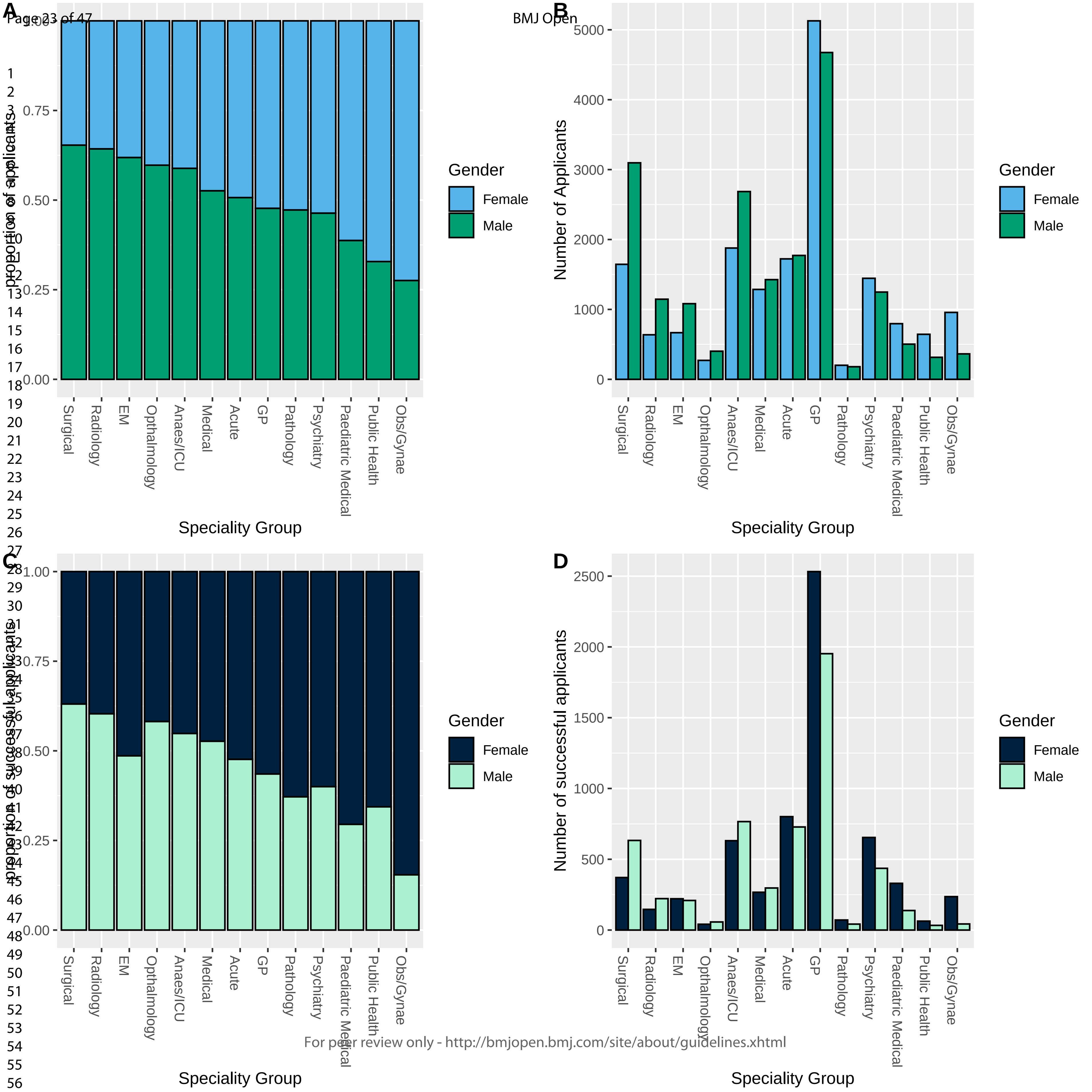
A
proportion of applicants
1.00
0.75
0.50
0.25
0.00
Gender
Female
Male
Surgical
Radiology
EM
Opthalmology
Anaes/ICU
Medical
Acute
GP
Pathology
Psychiatry
Paediatric Medical
Public Health
Obs/Gynae
Speciality Group
B
Number of Applicants
5000
4000
3000
2000
1000
0
Gender
Female
Male
Speciality Group
C
proportion of successful applicants
1.00
0.75
0.50
0.25
0.00
Gender
Female
Male
Speciality Group
D
Number of successful applicants
2500
2000
1500
1000
500
0
Gender
Female
Male
Speciality Group

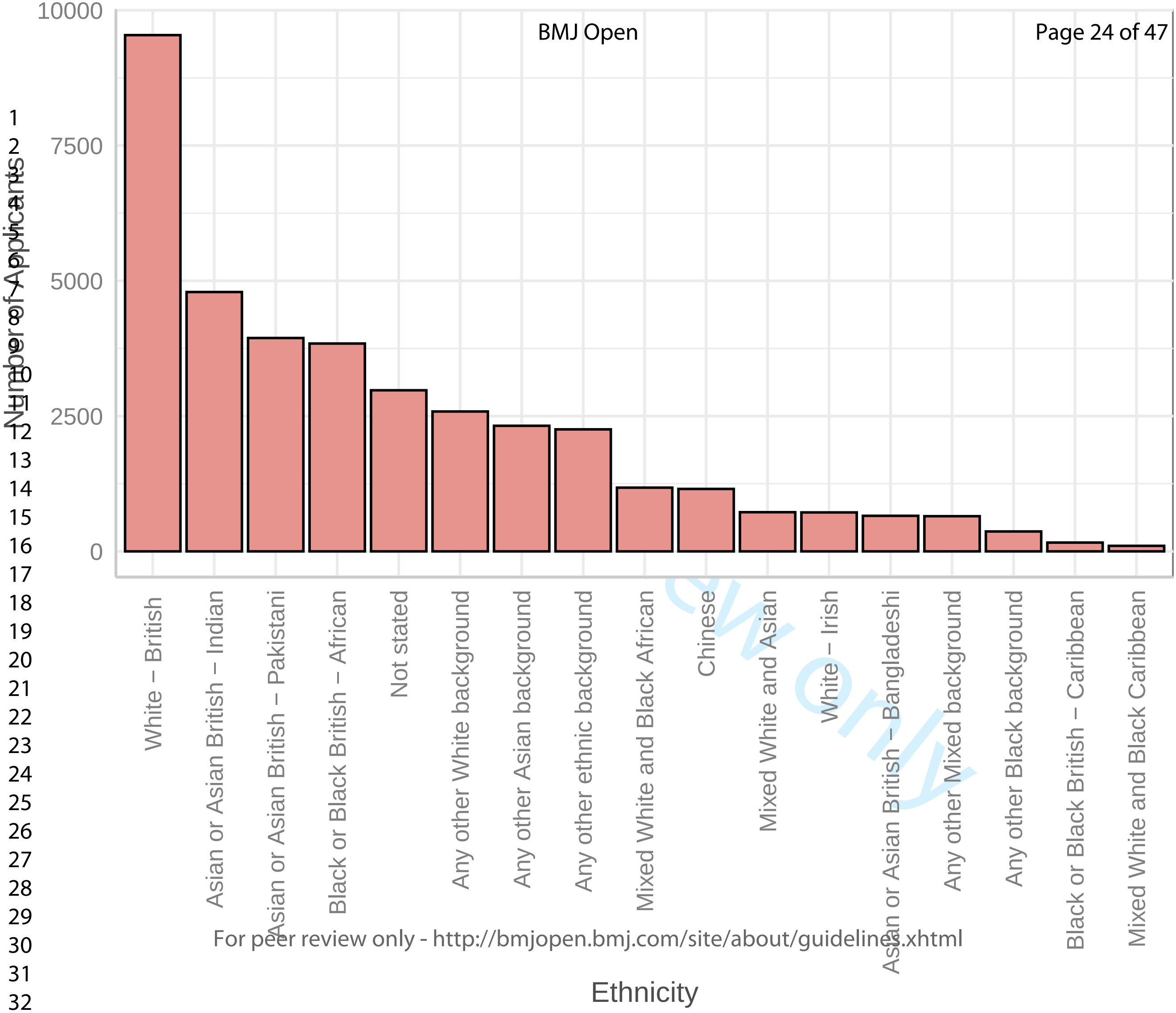

For peer review only - http://bmjopen.bmj.com/site/about/guidelines.xhtml

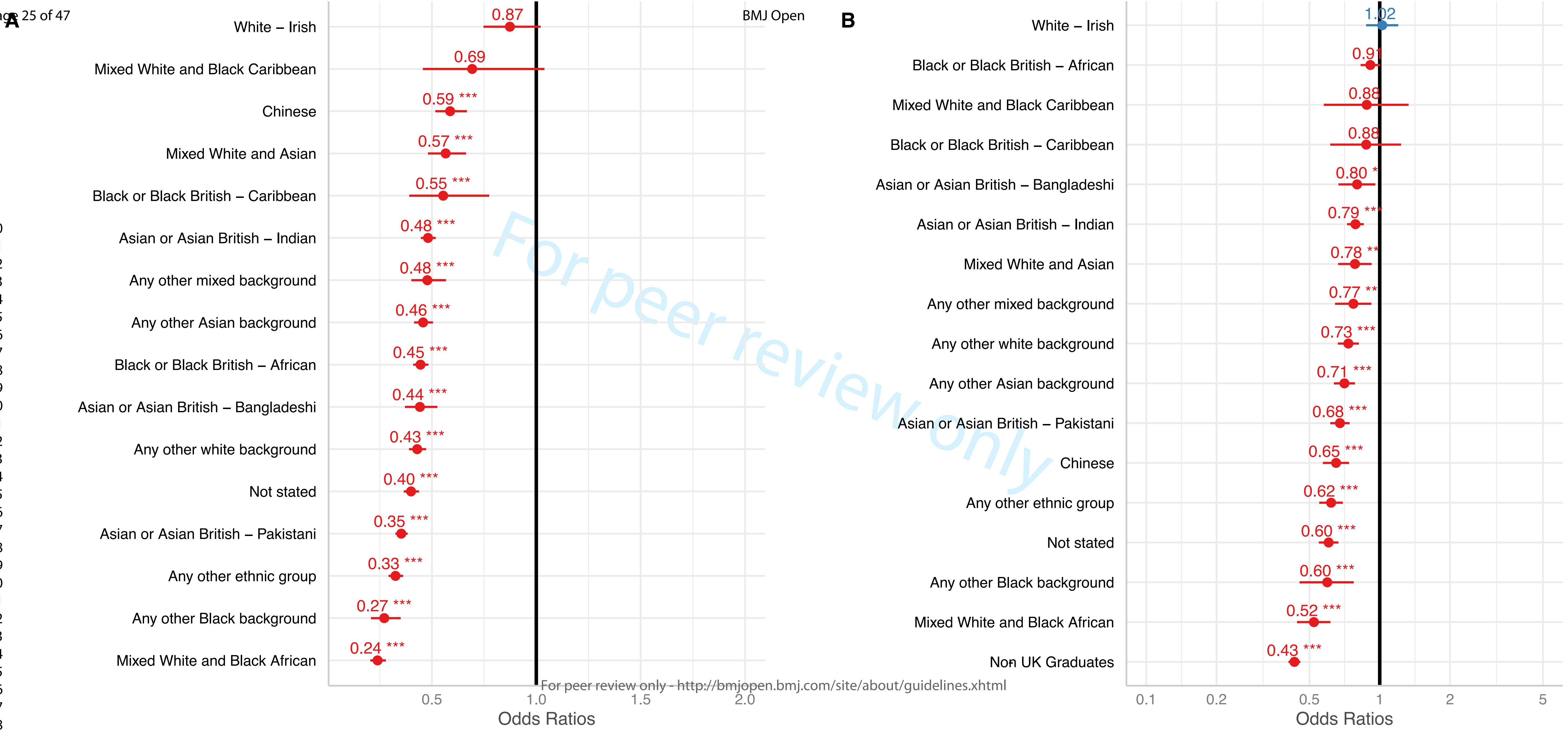

For peer review only

Supplementary Tables

Specialty and Level of Entry	Group
ACCS Internal Medicine/Internal Medicine CT1	Acute
ACCS Anaesthetics/Core Anaesthetics CT1	Anaes/ICU
Anaesthetics ST3	Anaes/ICU
Intensive Care Medicine ST3	Anaes/ICU
ACCS Emergency Medicine ST1/CT1	EM
Emergency Medicine ST3	EM
Emergency Medicine ST4	EM
General Practice ST1	GP
Allergy ST3	Medical
Audiovestibular Medicine ST3	Medical
Cardiology ST3	Medical
Clinical Genetics ST3	Medical
Clinical Neurophysiology ST3	Medical
Clinical Oncology ST3	Medical
Combined Infection Training ST3	Medical
Dermatology ST3	Medical
Gastroenterology ST3	Medical
Genitourinary Medicine ST3	Medical
Haematology ST3	Medical
Immunology ST3	Medical
Medical Oncology ST3	Medical
Medical Ophthalmology ST3	Medical
Neurology ST3	Medical
Occupational Medicine ST3	Medical
Palliative Medicine ST3	Medical
Rehabilitation Medicine ST3	Medical
Sport and Exercise Medicine ST3	Medical
Community Sexual and Reproductive Health ST1	Obs/Gynae
Obstetrics and Gynaecology ST1	Obs/Gynae
Obstetrics and Gynaecology ST3	Obs/Gynae
Ophthalmology ST1	Ophthalmology
Ophthalmology ST3	Ophthalmology
Paediatric Cardiology ST4	Paediatric Medical
Paediatrics ST1	Paediatric Medical
Paediatrics ST3	Paediatric Medical
Paediatrics ST4	Paediatric Medical
Chemical Pathology ST3	Pathology
Diagnostic Neuropathology ST3	Pathology
Histopathology ST1	Pathology
Core Psychiatry CT1	Psychiatry
General Psychiatry ST4	Psychiatry
Public Health Medicine ST1	Public Health
Clinical Radiology ST1	Radiology
Cardiothoracic Surgery ST1	Surgical
Cardiothoracic Surgery ST3	Surgical
Core Surgical Training CT1	Surgical
General and Vascular Surgery ST3	Surgical
Neurosurgery ST1	Surgical
Neurosurgery ST2	Surgical
Oral and Maxillo Facial Surgery ST1	Surgical
Oral and Maxillo Facial Surgery ST3	Surgical

For peer review only

Otolaryngology ST3	Surgical
Paediatric Surgery ST3	Surgical
Plastic Surgery ST3	Surgical
Trauma and Orthopaedic Surgery ST3	Surgical
Urology ST3	Surgical

Supplementary Table 1: Grouping of individual specialty posts for onward analysis
Abbreviations: ST, Specialist Trainee (year); CT, Core Trainee (year); ACCS, Acute Care Common Stem.

Specialty and Level of Entry	Male	Female	Not Stated	Total
General Practice ST1	4675	5128	250	10053
ACCS Internal Medicine/Internal Medicine CT1	1772	1723	95	3590
Core Surgical Training CT1	1486	932	110	2528
ACCS Anaesthetics/Core Anaesthetics CT1	1364	1026	67	2457
Core Psychiatry CT1	964	1092	74	2130
Clinical Radiology ST1	1146	637	96	1879
Anaesthetics ST3	930	660	37	1627
ACCS Emergency Medicine ST1/CT1	806	576	42	1424
Public Health Medicine ST1	315	644	29	988
Paediatrics ST1	272	598	16	886
Obstetrics and Gynaecology ST1	230	622	25	877
General Psychiatry ST4	284	353	8	645
Ophthalmology ST1	355	238	32	625
General and Vascular Surgery ST3	380	215	12	607
Trauma and Orthopaedic Surgery ST3	489	98	17	604
Intensive Care Medicine ST3	392	192	11	595
Cardiology ST3	284	102	15	401
Haematology ST3	174	184	10	368
Histopathology ST1	156	180	15	351
Obstetrics and Gynaecology ST3*	94	215	<5	309
Dermatology ST3	63	218	12	293
Combined Infection Training ST3	138	128	6	272
Clinical Oncology ST3	127	130	10	267
Gastroenterology ST3*	187	63	<5	250
Neurosurgery ST1	165	67	10	242
Emergency Medicine ST3	189	42	6	237
Paediatrics ST3*	114	106	<5	220
Medical Oncology ST3	91	113	8	212
Plastic Surgery ST3	113	87	11	211
Urology ST3	136	57	6	199
Paediatrics ST4	93	82	0	175
Neurology ST3	97	62	9	168
Community Sexual and Reproductive Health ST1	40	120	7	167

For peer review only

Otolaryngology ST3*	91	61	<5	152
Emergency Medicine ST4*	87	49	<5	136
Cardiothoracic Surgery ST1*	69	40	<5	109
Palliative Medicine ST3*	31	70	<5	101
Ophthalmology ST3*	47	33	<5	80
Paediatric Surgery ST3*	40	38	<5	78
Rehabilitation Medicine ST3*	45	29	<5	74
Immunology ST3*	31	42	<5	73
Neurosurgery ST2*	41	17	<5	58
Clinical Genetics ST3	13	43	0	56
Oral and Maxillo Facial Surgery ST1*	31	19	<5	50
Clinical Neurophysiology ST3*	32	16	<5	48
Cardiothoracic Surgery ST3*	38	8	<5	46
Genitourinary Medicine ST3*	21	25	<5	46
Occupational Medicine ST3*	26	19	<5	45
Allergy ST3*	17	21	<5	38
Paediatric Cardiology ST4*	24	10	<5	34
Sport and Exercise Medicine ST3*	26	7	<5	33
Chemical Pathology ST3	16	13	0	29
Oral and Maxillo Facial Surgery ST3*	18	6	<5	24
Medical Ophthalmology ST3	12	9	0	21
Audiovestibular Medicine ST3*	11	5	<5	16
Diagnostic Neuropathology ST3	8	8	0	16

Supplementary Table 2: The number of applicants to Specialty Posts in the 2021-2022 recruitment cycle by gender. Totals with * are not exact as <5 applicants did not state their gender. 'Nuclear Medicine ST3' and 'Paediatric and Perinatal Pathology ST3' did not have 5 or more applicants in a gender category and therefore are not included here. Abbreviations: ST, Specialist Trainee (year); CT, Core Trainee (year); ACCS, Acute Care Common Stem.

For peer review only

Specialty and Level	Male Success (%)	Female Success (%)	Sig diff in male or female success (p-value)
Emergency Medicine ST3	6.35	14.29	0.107
Neurosurgery ST1	6.06	7.46	0.769
Cardiothoracic Surgery ST1	8.70	0.00	0.084
Community Sexual and Reproductive Health ST1	0.00	5.00	0.338
Occupational Medicine ST3	23.08	0.00	0.032
Sport and Exercise Medicine ST3	23.08	0.00	0.301
Chemical Pathology ST3	31.25	38.46	0.714

Supplementary Table 3: Success of applicants to Training Posts by specialty in the 2021 recruitment year by gender where numbers are small. Table showing the applications of trainees to Specialty Training Posts and percentage of successful applicants by gender, where numbers are small and *p*-values derived from Fisher exact tests are provided for comparison and to 3 decimal places. Where p-values fall below the significance threshold of 0.05 they are highlighted in blue (Occupational Medicine ST3, which had a higher proportion of successful males).

Ethnic.Origin	Total	Non–UK Graduate Success (%)	UK Graduate Success (%)	Percentage Difference (95% CI)
Asian or Asian British – Bangladeshi	645	21.4	51.8	30.40 (21.50 to 39.30)
Asian or Asian British – Indian	4703	23.2	44.1	20.90 (18.00 to 23.70)
Asian or Asian British – Pakistani	3907	21.3	39.8	18.50 (14.60 to 22.40)
Chinese	1141	28.9	36.5	7.57 (–0.18 to 15.30)
Any other Asian background	2288	24.2	37.2	13.00 (9.06 to 16.90)
Black or Black British – African	3736	28.0	38.9	10.90 (6.02 to 15.80)
Black or Black British – Caribbean	150	23.9	48.4	24.50 (7.85 to 41.20)
Any other Black background	362	19.1	43.8	24.70 (–3.25 to 52.60)
Mixed White and Asian	705	21.0	44.8	23.80 (16.80 to 30.70)
Mixed White and Black African	1158	17.3	36.8	19.60 (5.94 to 33.20)
Mixed White and Black Caribbean	97	22.6	47.0	24.40 (3.00 to 45.80)
Any other mixed background	637	22.1	44.7	22.50 (14.70 to 30.30)
White – British	9101	31.1	48.4	17.30 (8.51 to 26.10)
White – Irish	685	34.0	47.8	13.80 (4.58 to 22.90)
Any other white background	2530	23.2	40.2	17.00 (13.00 to 21.10)
Any other ethnic group	2217	19.5	39.0	19.50 (14.40 to 24.60)
Not stated	2921	19.3	36.7	17.40 (14.10 to 20.80)
All Ethnicities*	36983	22.8	44.5	21.80 (20.80 to 22.70)

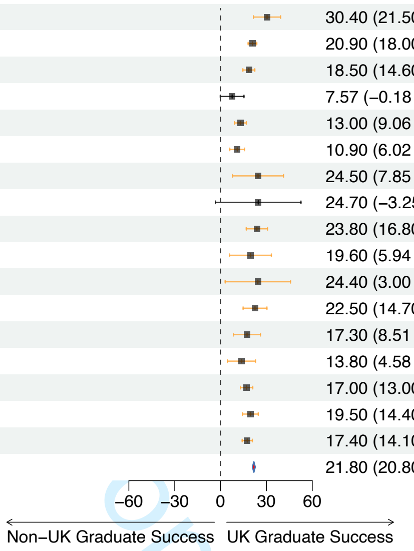

Supplementary Table 4: Success of applicants to Training Posts by ethnicity in the 2021 recruitment year by graduate status. Table showing the applications of trainees to Specialty Training Posts by ethnicity and percentage of successful applicants by graduate status (UK vs Non-UK). All values are provided to 3 significant figures. Where confidence intervals for estimated differences between the percentage of successful applicants by graduate status does not cross zero, it is highlighted in orange (greater UK graduate success).

Predictors	Adjusted Odds Ratios	95% Confidence Interval	p
Non-UK Graduates	0.43 ***	0.41 – 0.46	**<0.001**
White – Irish	1.02	0.87 – 1.20	0.762
Any other White background	0.73 ***	0.66 – 0.81	**<0.001**
Mixed White and Black Caribbean	0.88	0.58 – 1.33	0.546
Mixed White and Black African	0.52 ***	0.44 – 0.61	**<0.001**
Mixed White and Asian]	0.78 **	0.66 – 0.92	**0.004**
Black or Black British - Caribbean	0.88	0.61 – 1.23	0.453
Black or Black British - African	0.91	0.83 – 1.00	0.058
Any other Black background	0.60 ***	0.45 – 0.77	**<0.001**
Chinese	0.65 ***	0.57 – 0.74	**<0.001**
Asian or Asian British - Bangladeshi	0.80 *	0.67 – 0.96	**0.016**
Asian or Asian British - Indian	0.79 ***	0.72 – 0.85	**<0.001**
Asian or Asian British - Pakistani	0.68 ***	0.61 – 0.74	**<0.001**
Any other Asian background]	0.71 ***	0.64 – 0.78	**<0.001**
Any other mixed background	0.77 **	0.64 – 0.92	**0.004**
Any other ethnic group	0.62 ***	0.55 – 0.69	**<0.001**
Not stated	0.60 ***	0.55 – 0.67	**<0.001**
Observations	36983		

Supplementary Table 5: The effect of ethnicity and graduate status on success at application to Specialty Posts in the UK. Multivariable logistic regression model comparing success of application to Specialty Training Posts by minority ethnic groups to White British applicants where Country of Qualification (UK versus Non-UK) is included as a covariate. Non-Medical applicants to Public Health Specialty Training have been removed (n=988). Odds ratios are presented with 95% Confidence Intervals. ** p<0.05   ** p<0.01   *** p<0.001.*

Predictors	Unadjusted Odds Ratios	95% Confidence Interval	p
Non-UK Graduates	0.37 ***	0.35 – 0.38	**<0.001**
White - Irish	0.87	0.75 – 1.02	0.089
Any other white background	0.43 ***	0.39 – 0.47	**<0.001**
Mixed White and Black Caribbean	0.69	0.46 – 1.04	0.079
Mixed White and Black African	0.24 ***	0.20 – 0.28	**<0.001**
Mixed White and Asian	0.57 ***	0.48 – 0.66	**<0.001**
Black or Black British – Caribbean	0.55 ***	0.39 – 0.77	**0.001**
Black or Black British - African	0.45 ***	0.41 – 0.48	**<0.001**
Any other Black background	0.27 ***	0.21 – 0.35	**<0.001**
Chinese	0.59 ***	0.52 – 0.67	**<0.001**
Asian or Asian British - Bangladeshi	0.44 ***	0.37 – 0.53	**<0.001**
Asian or Asian British - Indian	0.48 ***	0.45 – 0.52	**<0.001**
Asian or Asian British - Pakistani	0.35 ***	0.32 – 0.38	**<0.001**
Any other Asian background	0.46 ***	0.41 – 0.50	**<0.001**
Any other mixed background	0.48 ***	0.40 – 0.57	**<0.001**
Any other ethnic group	0.33 ***	0.29 – 0.36	**<0.001**
Not stated	0.40 ***	0.37 – 0.44	**<0.001**
Observations	36983		

Supplementary Table 6: The unadjusted effects of ethnicity and graduate status on success at application to Specialty Posts in the UK. Unadjusted multivariable logistic regression model comparing success of application to Specialty Training Posts by minority ethnic groups to White British applicants where Country of Qualification (UK versus Non-UK) **is not** included as a covariate. Non-Medical applicants to Public Health Specialty Training have been removed (n=988). Odds ratios are presented with 95% Confidence Intervals. ** p<0.05   ** p<0.01   *** p<0.001.*

For peer review only

Ethnicity	UK Graduate probability of success	95% Confidence Interval	Non-UK Graduate probability of success	95% Confidence Interval
White British	0.48	0.47-0.49	0.31	0.23-0.40
White Irish	0.48	0.44-0.52	0.34	0.27-0.42
Any other white background	0.40	0.37-0.44	0.23	0.21-0.25
Mixed White and Black Caribbean	0.47	0.35-0.59	0.23	0.11-0.40
Mixed White and Black African	0.37	0.25-0.50	0.17	0.15-0.20
Mixed White and Asian	0.45	0.40-0.50	0.21	0.17-0.26
Black or Black British Caribbean	0.48	0.36-0.61	0.24	0.16-0.34
Black or Black British African	0.39	0.34-0.43	0.28	0.26-0.30
Any other Black background	0.44	0.22-0.68	0.19	0.15-0.24
Chinese	0.36	0.33-0.40	0.29	0.23-0.36
Asian or Asian British Bangladeshi	0.52	0.44-0.59	0.21	0.18-0.25
Asian or Asian British Indian	0.44	0.42-0.46	0.23	0.22-0.25
Asian or Asian British Pakistani	0.40	0.36-0.43	0.21	0.20-0.23
Any other Asian background	0.37	0.34-0.40	0.24	0.22-0.27
Any other mixed background	0.45	0.39-0.51	0.22	0.18-0.27
Any other ethnic group	0.39	0.34-0.44	0.20	0.18-0.21
Not stated	0.37	0.34-0.39	0.19	0.17-0.21

Supplementary Table 7: Multivariable logistic regression model displaying the probability of success of application to Specialty Training Posts in the 2021-22 recruitment cycle by Ethnicity and Country of Graduate Status. A logistic regression model is fitted with variation of success at application between ethnic groups by country of qualification measured by including interaction terms between the two variables. Probabilities are presented with 95% Confidence Intervals. Anova analysis of residuals between models where an interaction was included and where it was not, demonstrated a significant difference between the models. Only the 'Asian or Asian British – Bangladeshi' group significantly differed in outcome across the graduate status (p=0.02).

For peer review only

Supplementary Figures

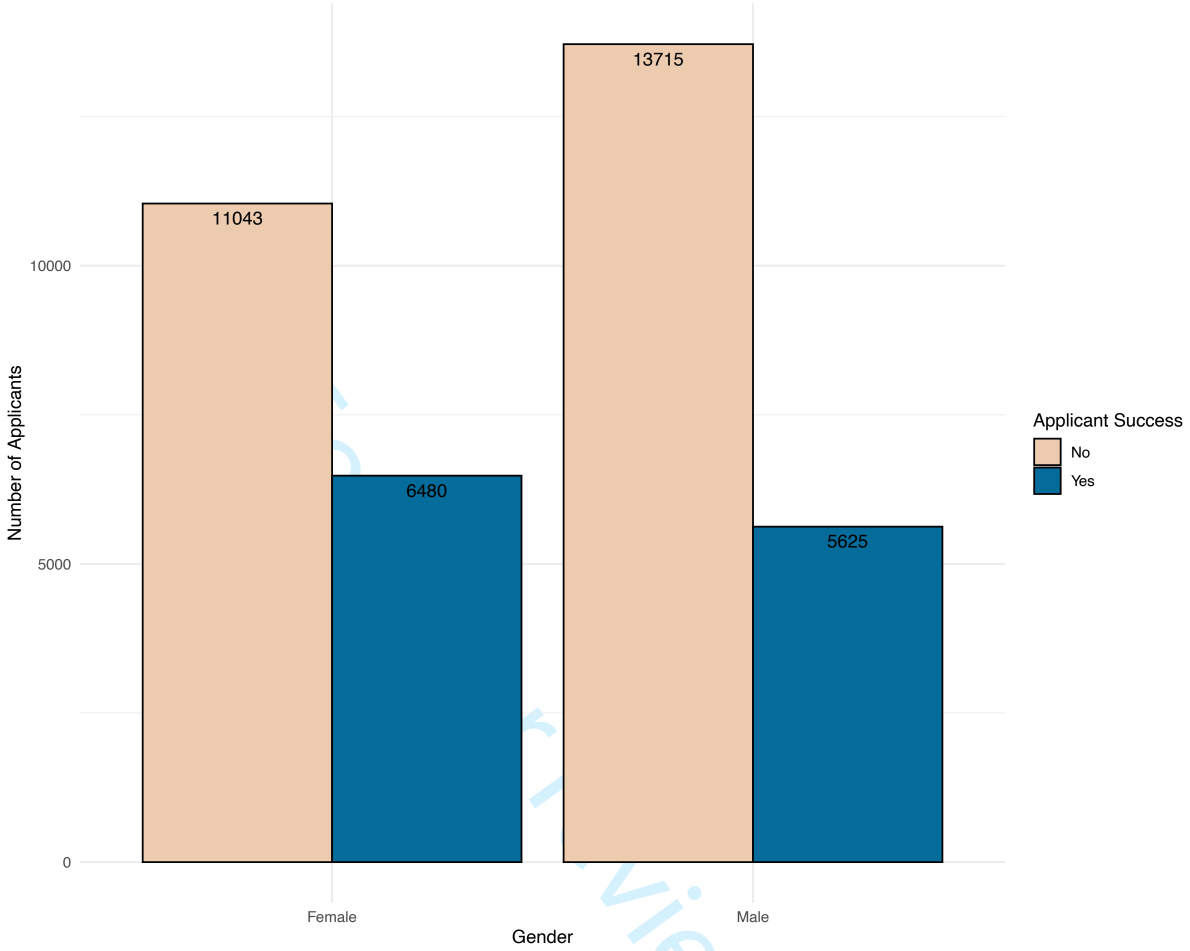

Supplementary Figure 1: Applications and outcomes for Specialty Training Posts in the 2021-22 recruitment year by gender. Bar chart demonstrating applications and outcomes of trainees to Specialty Training by gender. Overall, the difference in percentage of success by females (6480/17523, 37.0%) and males (5625/19340, 29.1%) was 7.9% (95% C.I.= 6.93-8.86%), in favour of females.

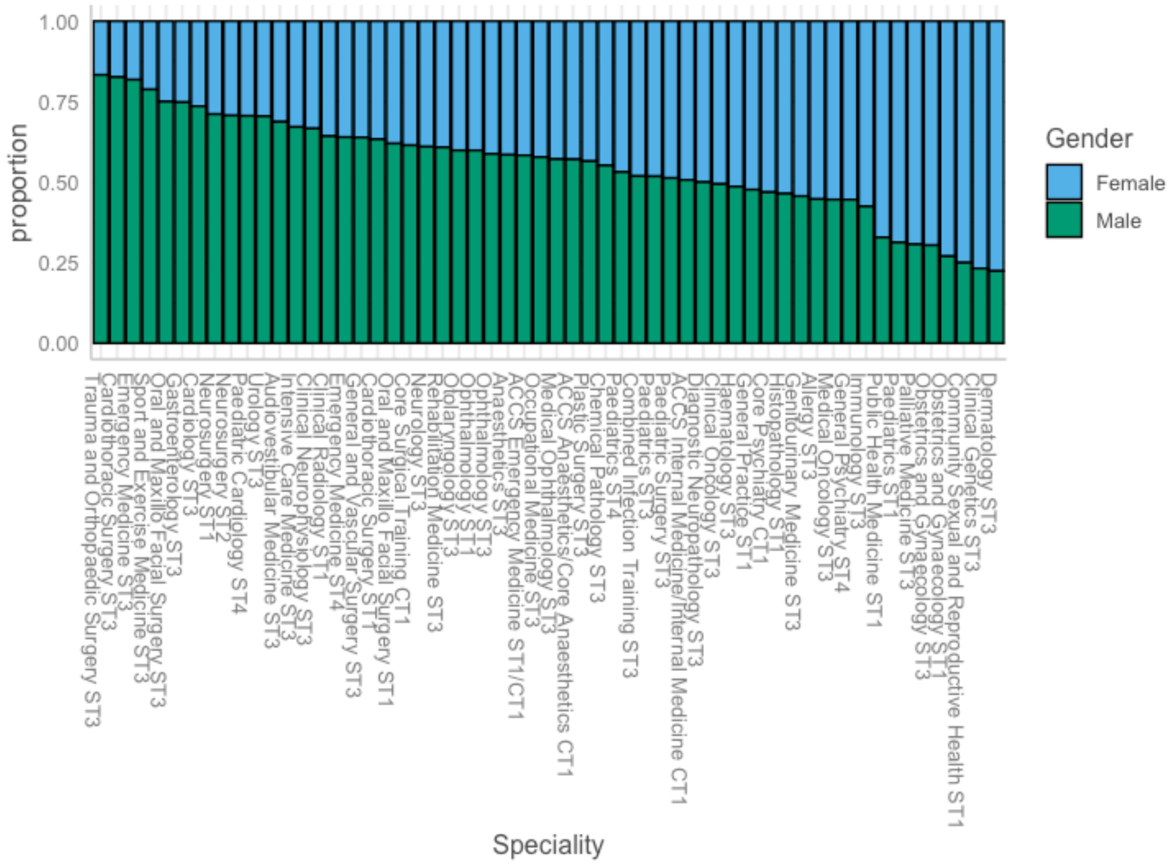

Supplementary Figure 2: Proportion of applicants to individual Specialty Training Posts in the 2021 recruitment year by Gender. Data is presented where complete.

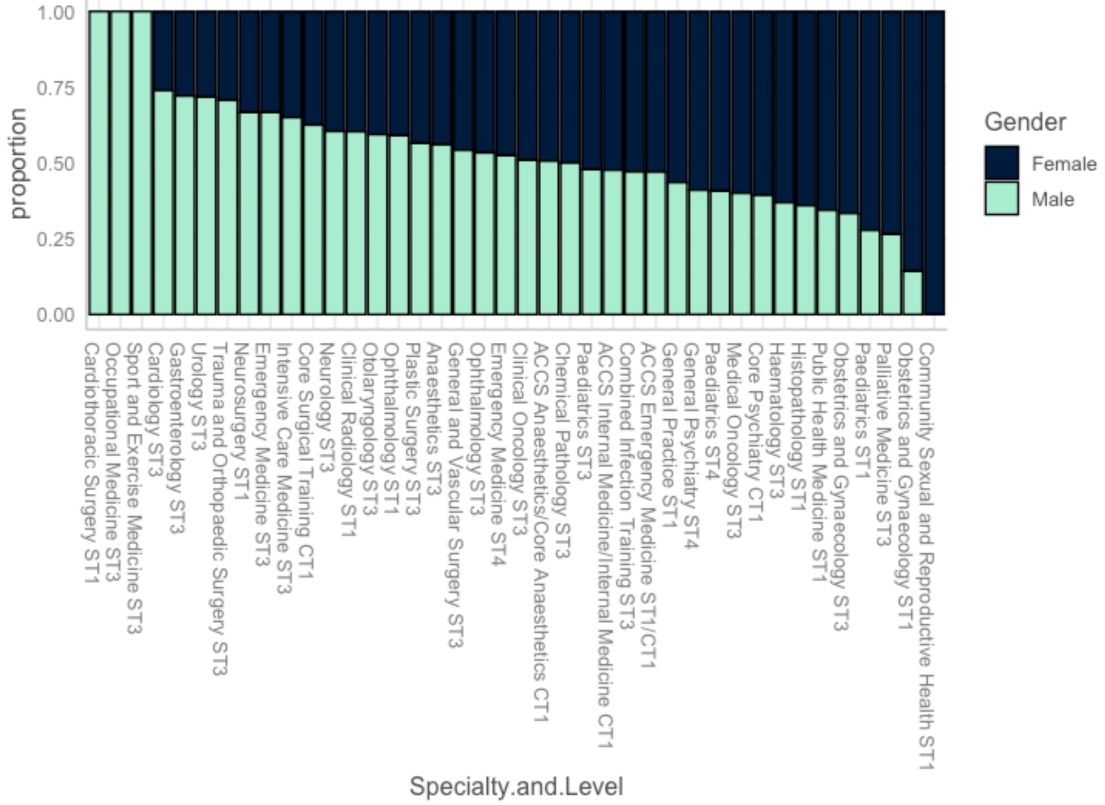

Supplementary Figure 3: Proportion of successful applicants to individual specialities in the 2021 recruitment year by Gender. Data is presented where complete.

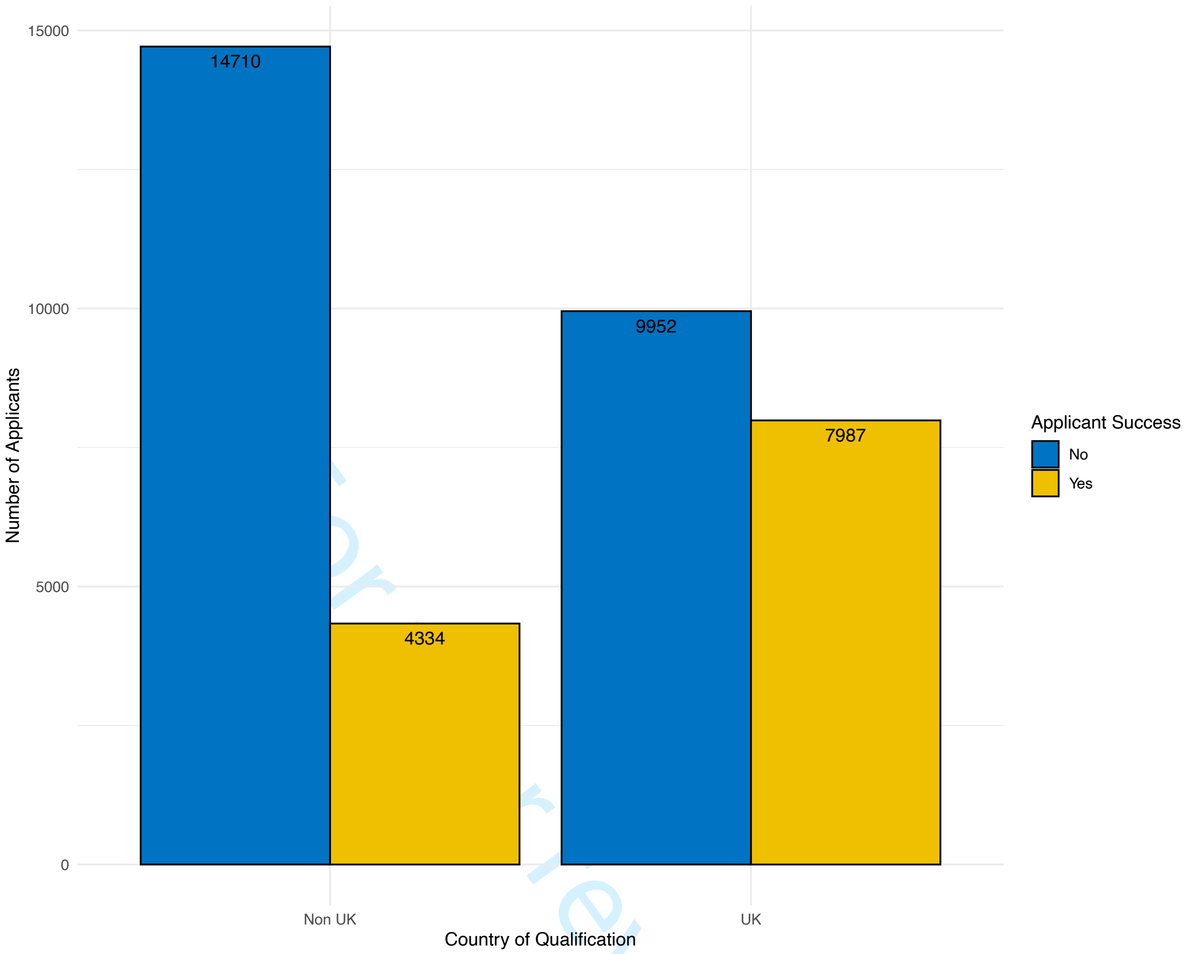

Supplementary Figure 4: Applications and outcomes for Specialty Training Posts in the 2021-2022 recruitment year by ethnicity. (a) Applications to Specialty Training Posts in the 2021 recruitment year by Ethnicity. (b**)** Applications and outcomes by country of qualification (Non-UK vs UK). Overall, the difference in percentage of success by UK graduates (7987/17939, 44.5%) and Non-UK graduates (4334/19044, 22.8%) was 21.8% (95% C.I. = 20.8-22.7%), in favour of UK graduates.

For peer review only

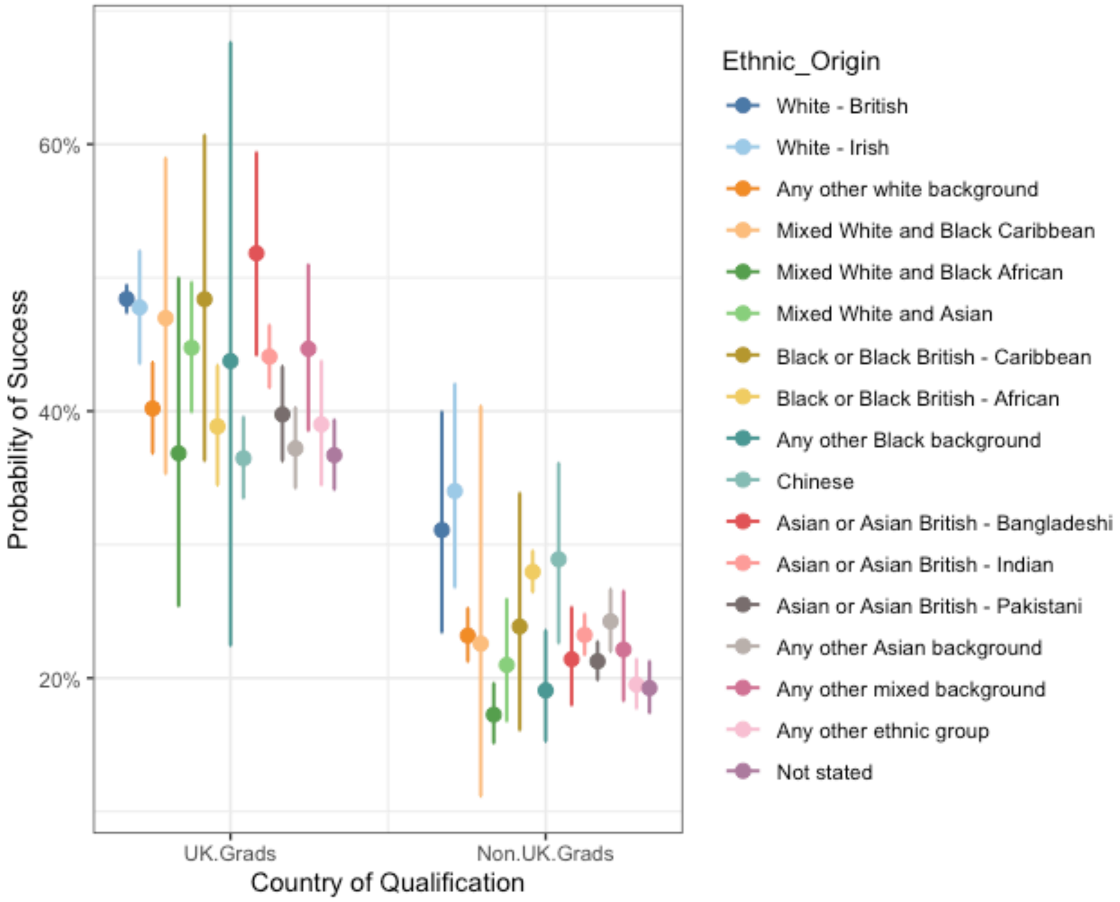

Supplementary Figure 5: Multivariable logistic regression model displaying the probability of success of application to Specialty Training Posts in the 2021-22 recruitment cycle by Ethnicity. A logistic regression model is fitted with variation of success at application between ethnic groups by country of qualification measured by including interaction terms between the two variables. Probabilities are presented with 95% Confidence Intervals. Anova analysis of residuals between models where an interaction was included and where it was not, demonstrated a significant difference between the models. Only the 'Asian or Asian British – Bangladeshi' group significantly differed in outcome across the graduate status.

For peer review only

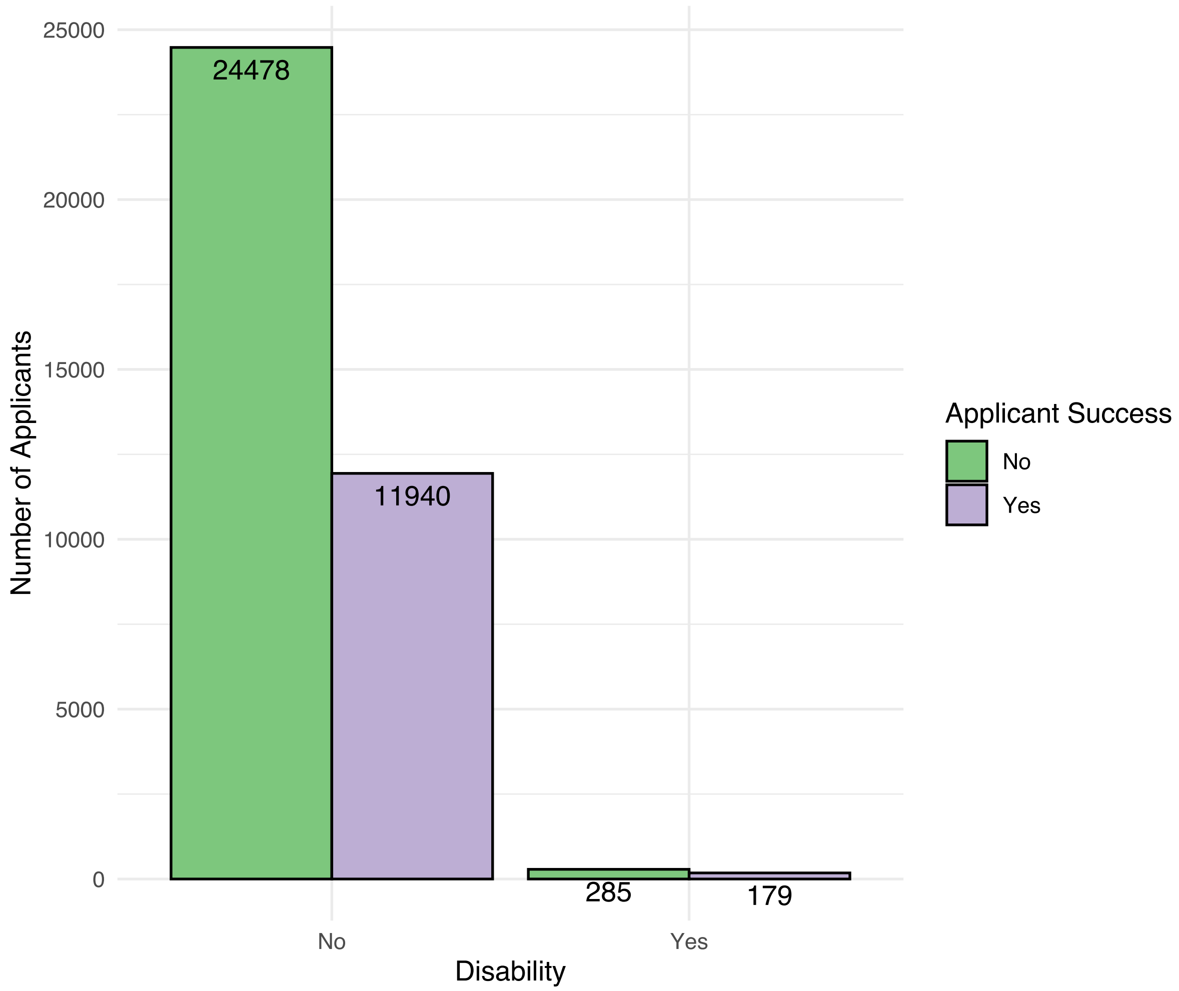

Supplementary Figure 6: **Applications and outcomes to Specialty Training Posts in 2021 by disability status.** Overall, the difference in percentage of success by disabled applicants (179/464, 38.6%) and non-disabled applicants (11940/36418, 32.8%) was 5.79% (95% C.I. = 1.23-10.4%), in favour of disabled applicants.

review only

Gender – All applicants

Gender	2021 All Applicants	2021 Accepted Applicants
Male	19340	5625
Female	17523	6480
Not Stated	1108	314

Country of Qualification – All applicants

Country of Qualification	2021 All Applicants	2021 Accepted Applicants
UK	17939	7987
Non UK	19044	4334
Non Medical Public Health Applicants	988	98

Ethnicity by Graduate Country

Ethnic Origin	Applied			Accepted		
	UK Grads	Non-UK Grads	Non-Medical Applicants	UK Grads	Non-UK Grads	Non-Medical Applicants
Asian or Asian British - Bangladeshi	164	481	12	85	103	0
Asian or Asian British - Indian	1733	2970	90	764	690	<5
Asian or Asian British - Pakistani	732	3175	36	291	675	<5
Chinese	968	173	13	353	50	<5
Any other Asian background	989	1299	33	368	315	<5
Black or Black British - African	453	3283	105	176	918	<5
Black or Black British - Caribbean	62	88	12	30	21	0
Any other Black background	16	346	6	7	66	0
Mixed White and Asian	400	305	19	179	64	<5
Mixed White and Black African	57	1101	20	21	190	0
Mixed White and Black Caribbean	66	31	5	31	7	0
Any other mixed background	244	393	13	109	87	<5
White - British	8982	119	440	4348	37	68
White - Irish	538	147	35	257	50	<5
Any other white background	796	1734	55	320	402	5
Any other ethnic group	423	1794	38	165	350	<5
Not stated	1316	1605	56	483	309	<5

For peer review only

Disability - All applicants

Disability	2021 All Applicants	2021 Accepted Applicants
Yes	464	179
No	36418	11940
Not Stated	1089	300

Specialty by Gender

Specialty and Level	Number of Applicants			Number Accepted An Offer		
	Male	Female	Not Stated	Male	Female	Not Stated
ACCS Anaesthetics/Core Anaesthetics CT1	1364	1026	67	323	315	10
ACCS Emergency Medicine ST1/CT1	806	576	42	165	186	9
ACCS Internal Medicine/Internal Medicine CT1	1772	1723	95	728	801	38
Allergy ST3	17	21	<5	<5	<5	0
Anaesthetics ST3	930	660	37	315	247	15
Audiovestibular Medicine ST3	11	5	<5	<5	0	0
Cardiology ST3	284	102	15	68	24	<5
Cardiothoracic Surgery ST1	69	40	<5	6	0	0
Cardiothoracic Surgery ST3	38	8	<5	<5	0	0
Chemical Pathology ST3	16	13	0	5	5	0
Clinical Genetics ST3	13	43	0	<5	14	0
Clinical Neurophysiology ST3	32	16	<5	7	<5	0
Clinical Oncology ST3	127	130	10	55	53	<5
Clinical Radiology ST1	1146	637	96	222	146	22
Combined Infection Training ST3	138	128	6	32	36	0
Community Sexual and Reproductive Health ST1	40	120	7	0	6	0
Core Psychiatry CT1	964	1092	74	258	398	27
Core Surgical Training CT1	1486	932	110	332	199	23
Dermatology ST3	63	218	12	<5	35	<5
Diagnostic Neuropathology ST3	8	8	0	0	<5	0
Emergency Medicine ST3	189	42	6	12	6	<5
Emergency Medicine ST4	87	49	<5	32	29	0
Gastroenterology ST3	187	63	<5	31	12	<5
General and Vascular Surgery ST3	380	215	12	71	60	0
General Practice ST1	4675	5128	250	1952	2532	96
General Psychiatry ST4	284	353	8	178	256	8
Genitourinary Medicine ST3	21	25	<5	<5	9	0
Haematology ST3	174	184	10	31	53	<5
Histopathology ST1	156	180	15	37	66	6

For peer review only

Immunology ST3	31	42	<5	<5	6	0
Intensive Care Medicine ST3	392	192	11	128	69	5
Medical Oncology ST3	91	113	8	30	45	<5
Medical Ophthalmology ST3	12	9	0	<5	<5	0
Neurology ST3	97	62	9	29	19	<5
Neurosurgery ST1	165	67	10	10	5	0
Neurosurgery ST2	41	17	<5	0	<5	0
Nuclear Medicine ST3	13	<5	0	<5	<5	0
Obstetrics and Gynaecology ST1	230	622	25	36	216	8
Obstetrics and Gynaecology ST3	94	215	<5	7	14	<5
Occupational Medicine ST3	26	19	<5	6	0	0
Ophthalmology ST1	355	238	32	49	34	6
Ophthalmology ST3	47	33	<5	8	7	0
Oral and Maxillo Facial Surgery ST1	31	19	<5	<5	5	<5
Oral and Maxillo Facial Surgery ST3	18	6	<5	8	<5	<5
Otolaryngology ST3	91	61	<5	19	13	<5
Paediatric and Perinatal Pathology ST3	<5	7	0	0	<5	0
Paediatric Cardiology ST4	24	10	<5	5	<5	0
Paediatric Surgery ST3	40	38	<5	<5	7	0
Paediatrics ST1	272	598	16	116	302	5
Paediatrics ST3	114	106	<5	11	12	0
Paediatrics ST4	93	82	0	11	16	0
Palliative Medicine ST3	31	70	<5	9	25	0
Plastic Surgery ST3	113	87	11	39	30	<5
Public Health Medicine ST1	315	644	29	33	63	<5
Rehabilitation Medicine ST3	45	29	<5	13	<5	0
Sport and Exercise Medicine ST3	26	7	<5	6	0	0
Trauma and Orthopaedic Surgery ST3	489	98	17	123	51	<5
Urology ST3	136	57	6	33	13	<5

For peer review only

Specialty by Disability

Specialty and Level	Number of Applicants			Number Accepted An Offer		
	Disabled	Not Disabled	Not Stated	Disabled	Not Disabled	Not Stated
ACCS Anaesthetics/Core Anaesthetics CT1	24	2373	60	6	631	11
ACCS Emergency Medicine ST1/CT1	9	1374	41	<5	351	8
ACCS Internal Medicine/Internal Medicine CT1	36	3457	97	17	1516	34
Allergy ST3	<5	37	<5	0	5	0
Anaesthetics ST3	19	1585	23	5	563	9
Audiovestibular Medicine ST3	0	16	<5	0	<5	<5
Cardiology ST3	<5	384	18	<5	90	6

Cardiothoracic Surgery ST1	<5	109	<5	0	6	0
Cardiothoracic Surgery ST3	0	44	<5	0	<5	<5
Chemical Pathology ST3	<5	26	<5	<5	9	0
Clinical Genetics ST3	<5	55	0	0	18	0
Clinical Neurophysiology ST3	<5	35	<5	<5	7	0
Clinical Oncology ST3	<5	260	6	0	107	<5
Clinical Radiology ST1	18	1785	506	7	369	14
Combined Infection Training ST3	<5	246	5	0	65	<5
Community Sexual and Reproductive Health ST1	<5	153	11	0	5	<5
Core Psychiatry CT1	57	2000	73	23	639	21
Core Surgical Training CT1	21	2410	97	8	519	27
Dermatology ST3	<5	277	12	<5	40	<5
Diagnostic Neuropathology ST3	<5	15	0	0	<5	0
Emergency Medicine ST3	<5	229	6	0	19	<5
Emergency Medicine ST4	5	130	<5	<5	57	<5
Gastroenterology ST3	0	248	6	0	42	<5
General and Vascular Surgery ST3	7	585	15	0	130	<5
General Practice ST1	121	9632	300	61	4412	107
General Psychiatry ST4	16	618	11	15	418	9
Genitourinary Medicine ST3	<5	47	<5	0	13	0
Haematology ST3	<5	358	9	0	83	<5
Histopathology ST1	11	330	10	6	101	<5
Immunology ST3	<5	73	<5	0	9	0
Intensive Care Medicine ST3	<5	582	10	<5	196	<5
Medical Oncology ST3	<5	206	5	<5	76	<5
Medical Ophthalmology ST3	0	21	0	0	<5	0
Neurology ST3	<5	155	11	0	47	<5
Neurosurgery ST1	<5	233	6	<5	15	0
Neurosurgery ST2	<5	55	<5	0	<5	0
Nuclear Medicine ST3	0	16	0	0	<5	0
Obstetrics and Gynaecology ST1	7	844	26	<5	250	6
Obstetrics and Gynaecology ST3	0	305	7	0	21	<5
Occupational Medicine ST3	<5	43	<5	0	6	0
Ophthalmology ST1	7	572	26	<5	86	<5
Ophthalmology ST3	<5	79	<5	0	15	0
Oral and Maxillo Facial Surgery ST1	<5	51	<5	<5	9	0
Oral and Maxillo Facial Surgery ST3	0	27	<5	0	15	0
Otolaryngology ST3	<5	149	<5	0	33	0
Paediatric and Perinatal Pathology ST3	0	11	0	0	<5	0
Paediatric Cardiology ST4	0	34	<5	0	6	0
Paediatric Surgery ST3	0	77	<5	0	8	<5
Paediatrics ST1	11	851	24	9	408	6

For peer review only

Paediatrics ST3	0	218	<5	0	25	0
Paediatrics ST4	0	174	<5	0	27	0
Palliative Medicine ST3	<5	99	<5	0	33	<5
Plastic Surgery ST3	0	206	5	0	71	<5
Public Health Medicine ST1	46	919	23	<5	93	<5
Rehabilitation Medicine ST3	<5	69	6	0	16	0
Sport and Exercise Medicine ST3	<5	34	0	<5	5	0
Trauma and Orthopaedic Surgery ST3	<5	588	13	0	175	<5
Urology ST3	<5	196	<5	0	48	0

For peer review only

Specialty by Ethnicity

Specialty and Level	Number of Applicants																	Number Accepted An Offer																
	Asian or Asian British - Bangladeshi	Asian or Asian British - Indian	Asian or Asian British - Pakistani	Chinese	Any other Asian background	Black or Black British - African	Black or Black British - Caribbean	Any other Black background	Mixed White and Asian	Mixed White and Black African	Mixed White and Black Caribbean	Any other Mixed background	White - British	White - Irish	Any other White background	Any other ethnic group	Not Stated	Asian or Asian British - Bangladeshi	Asian or Asian British - Indian	Asian or Asian British - Pakistani	Chinese	Any other Asian background	Black or Black British - African	Black or Black British - Caribbean	Any other Black background	Mixed White and Asian	Mixed White and Black African	Mixed White and Black Caribbean	Any other Mixed background	White - British	White - Irish	Any other White background	Any other ethnic group	Not Stated
ACCS Anaesthetics/Core Anaesthetics CT1	21	225	121	102	129	98	9	5	46	37	13	38	1135	79	162	77	160	<5	41	14	26	19	5	<5	0	9	<5	6	12	424	29	31	5	22
ACCS Emergency Medicine ST1/CT1	14	190	99	31	75	113	5	<5	33	33	<5	29	492	24	97	69	113	<5	18	9	10	6	12	<5	0	13	<5	<5	9	226	11	21	<5	15
ACCS Internal Medicine/Internal Medicine CT1	59	456	327	172	266	244	9	19	94	66	10	47	1062	79	269	161	250	26	192	111	79	112	91	<5	6	43	21	6	21	577	30	112	56	81
Allergy ST3	<5	7	6	<5	<5	6	0	<5	0	<5	0	<5	<5	0	<5	<5	5	0	0	0	<5	0	0	0	0	0	0	0	0	<5	0	<5	0	<5
Anaesthetics ST3	<5	193	38	49	56	18	<5	<5	39	20	<5	27	893	43	94	66	81	<5	58	<5	19	12	<5	<5	<5	14	<5	<5	12	350	20	33	12	31
Audiovestibular Medicine ST3	0	<5	<5	<5	<5	<5	0	<5	0	<5	0	0	0	0	<5	<5	<5	0	<5	0	<5	0	<5	0	0	0	0	0	0	0	0	0	0	0
Cardiology ST3	11	48	68	9	30	35	<5	<5	5	19	0	12	44	5	38	29	41	<5	15	7	<5	8	6	<5	0	<5	0	0	0	25	<5	10	0	11
Cardiothoracic Surgery ST1	<5	14	10	9	10	9	0	<5	5	<5	<5	0	10	<5	19	<5	16	0	<5	0	<5	<5	0	0	0	0	0	0	0	<5	0	0	0	0
Cardiothoracic Surgery ST3	<5	0	<5	<5	7	6	0	0	0	<5	<5	0	6	<5	<5	6	6	0	0	0	0	0	0	0	0	0	0	0	0	0	0	0	<5	<5
Chemical Pathology ST3	0	<5	<5	0	0	<5	0	<5	0	<5	0	<5	0	0	0	<5	0	0	0	<5	0	0	<5	0	0	0	<5	0	0	0	0	0	0	0
Clinical Genetics ST3	0	6	<5	<5	5	6	0	<5	<5	0	<5	0	17	<5	5	<5	<5	0	<5	0	<5	<5	0	0	0	<5	0	0	<5	8	0	<5	<5	0
Clinical Neurophysiology ST3	<5	<5	9	<5	<5	5	0	<5	0	<5	<5	<5	<5	<5	<5	<5	<5	0	0	<5	0	0	<5	0	0	0	<5	0	<5	<5	<5	<5	0	0
Clinical Oncology ST3	<5	32	23	7	14	10	0	<5	<5	7	<5	<5	60	<5	7	14	14	0	16	6	<5	6	<5	0	<5	<5	<5	0	<5	35	<5	<5	6	5
Clinical Radiology ST1	32	288	255	79	137	185	10	17	26	44	6	39	226	26	145	164	200	<5	57	29	25	22	18	0	<5	7	<5	<5	8	116	7	31	19	38

Combined Infection Training ST3	<5	28	33	5	16	30	<5	11	5	19	0	11	59	<5	10	5	19	0	7	5	<5	5	5	<5	<5	<5	<5	0	<5	28	0	<5	0	<5
Community Sexual and Reproductive Health ST1	5	22	15	<5	9	27	<5	<5	<5	<5	<5	<5	32	6	9	10	18	0	0	0	0	0	<5	0	0	0	0	0	0	<5	<5	0	0	0
Core Psychiatry CT1	48	234	249	36	125	358	8	23	40	86	7	40	363	17	150	158	188	14	76	41	15	27	93	<5	<5	19	12	<5	17	204	8	49	53	46
Core Surgical Training CT1	45	292	185	117	167	168	13	12	47	31	<5	42	694	56	243	147	265	9	75	34	26	40	26	<5	<5	15	<5	<5	7	189	11	42	26	48
Dermatology ST3	<5	43	36	25	20	18	<5	<5	8	6	<5	<5	48	9	24	24	22	<5	6	<5	<5	<5	<5	0	0	<5	0	<5	0	9	0	<5	5	5
Diagnostic Neuropathology ST3	0	5	0	0	<5	<5	0	<5	0	<5	0	<5	<5	0	<5	0	0	0	0	0	0	0	0	0	0	0	0	0	<5	0	0	<5	0	0
Emergency Medicine ST3	<5	88	19	<1	10	32	0	<5	<5	17	0	6	9	<5	11	15	19	0	5	<5	0	<5	<5	0	<5	<5	<5	0	0	<5	0	0	<5	<5
Emergency Medicine ST4	0	34	12	<5	<5	<5	0	<5	<5	<5	<5	0	53	<5	8	<5	7	0	9	<5	<5	<5	<5	0	0	<5	0	<5	0	33	<5	5	0	<5
Gastroenterology ST3	6	32	52	5	18	22	0	15	<5	19	<5	9	19	<5	11	21	18	<5	5	5	<5	<5	8	0	<5	0	<5	0	0	8	<5	<5	<5	<5
General and Vascular Surgery ST3	12	77	55	15	31	34	<5	8	9	29	<5	13	142	15	50	73	40	<5	9	5	<5	7	5	<5	0	<5	<5	0	<5	66	8	12	<5	6
General Practice ST1	236	1211	1474	213	725	1533	47	109	187	309	17	146	1834	169	517	568	758	104	530	568	96	304	681	25	44	67	125	7	64	1123	109	202	203	328
General Psychiatry ST4	8	107	39	21	28	68	<5	<5	7	6	0	7	210	12	65	30	32	<5	87	23	15	20	40	0	<5	<5	<5	0	6	153	6	38	21	21
Genitourinary Medicine ST3	<5	6	6	<5	<5	6	0	<5	<5	<5	0	<5	7	<5	<5	<5	6	0	<5	0	0	0	<5	0	0	<5	0	0	0	<5	<5	0	<5	0
Haematology ST3	7	59	39	13	12	37	<5	12	6	23	<5	12	57	5	24	30	30	0	20	<5	6	<5	<5	<5	0	<5	<5	<5	<5	30	<5	5	<5	<5
Histopathology ST1	<5	32	20	12	14	41	<5	6	8	12	<5	7	87	5	42	34	26	0	12	5	7	<5	5	<5	0	5	<5	0	<5	42	<5	10	6	8
Immunology ST3	<5	7	6	<5	<5	14	0	5	0	10	0	8	<5	<5	<5	<5	9	0	<5	<5	0	0	<5	0	0	0	0	0	<5	<5	0	0	0	<5
Intensive Care Medicine ST3	<5	72	29	20	19	18	<5	<5	12	15	<5	6	285	14	34	26	33	0	24	<5	6	<5	<5	0	0	6	<5	<5	<5	115	5	10	8	13
Medical Oncology ST3	<5	36	32	8	11	10	0	<5	<5	8	0	<5	48	5	10	14	21	0	14	13	<5	<5	<5	0	0	0	0	0	<5	22	<5	<5	<5	8
Medical Ophthalmology ST3	0	<5	6	0	<5	<5	0	0	<5	0	0	0	<5	0	<5	<5	6	0	0	<5	0	0	0	0	0	0	0	0	0	0	0	<5	0	0
Neurology ST3	6	19	27	<5	10	18	0	5	<5	13	<5	13	20	<5	13	9	16	<5	<5	<5	<5	<5	<5	0	<5	0	<5	0	<5	13	<5	7	0	6
Neurosurgery ST1	6	38	25	9	20	15	<5	<5	<5	5	<5	5	41	<5	31	11	26	0	<5	0	0	<5	<5	0	0	0	0	0	<5	6	0	<5	<5	0
Neurosurgery ST2	<5	<5	8	<5	<5	9	<5	0	0	<5	0	<5	<5	0	10	6	<5	0	0	0	0	0	0	0	0	0	0	0	<5	0	0	0	0	0

For peer review only

For peer review only - http://bmjopen.bmj.com/site/about/guidelines.xhtml

Nuclear Medicine ST3	0	<5	<5	0	<5	<5	0	0	0	0	0	0	0	0	<5	<5	<5	0	0	0	0	0	0	0	0	0	0	0	0	0	0	<5	<5	0
Obstetrics and Gynaecology ST1	15	85	43	25	52	146	11	6	19	27	<5	13	206	12	87	48	78	<5	20	7	6	17	21	5	<5	11	<5	<5	<5	114	5	18	7	20
Obstetrics and Gynaecology ST3	<5	79	41	0	8	46	<5	9	6	23	<5	5	<5	0	27	34	22	0	13	<5	0	<5	<5	0	0	0	0	0	0	0	0	<5	<5	<5
Occupational Medicine ST3	0	<5	<5	0	<5	6	0	<5	0	<5	0	0	7	0	<5	0	<5	0	0	0	0	0	<5	0	0	0	0	0	0	<5	0	<5	0	<5
Ophthalmology ST1	7	74	72	30	51	37	<5	<5	7	14	<5	13	72	15	66	68	71	0	13	<5	5	6	<5	0	0	<5	0	0	<5	24	<5	12	13	6
Ophthalmology ST3	0	18	<5	<5	<5	6	<5	0	<5	<5	0	<5	<5	0	9	16	14	0	<5	<5	0	0	<5	<5	0	0	0	0	0	0	0	<5	5	<5
Oral and Maxillo Facial Surgery ST1	<5	12	<5	0	<5	<5	0	0	<5	<5	0	<5	13	<5	5	5	<5	0	<5	0	0	<5	0	0	0	<5	0	0	0	<5	<5	0	<5	0
Oral and Maxillo Facial Surgery ST3	0	<5	<5	<5	<5	0	0	0	0	0	0	0	6	0	<5	<5	<5	0	<5	0	<5	0	0	0	0	0	0	0	0	<5	0	<5	<5	<5
Otolaryngology ST3	29	<5	<5	9	14	<5	0	0	5	<5	<5	<5	47	<5	15	12	10	0	5	<5	<5	<5	0	0	0	0	0	0	<5	18	0	<5	<5	<5
Paediatric and Perinatal Pathology ST3	0	<5	0	0	<5	0	0	0	0	0	0	0	0	0	0	0	0	0	0	0	0	<5	0	0	0	0	0	0	0	0	0	0	0	0
Paediatric Cardiology ST4	0	<5	<5	<5	0	<5	0	0	<5	<5	0	0	8	0	6	8	5	0	0	0	<5	0	0	0	0	0	<5	0	0	<5	0	0	<5	<5
Paediatric Surgery ST3	<5	7	<5	<5	0	<5	0	0	<5	<5	0	6	25	<5	6	13	5	0	<5	0	0	0	0	0	0	0	0	0	0	5	0	<5	<5	<5
Paediatrics ST1	6	107	36	34	52	82	<5	10	10	31	0	8	311	22	69	44	62	<5	52	9	17	18	25	<5	<5	7	8	0	5	203	14	23	15	19
Paediatrics ST3	<5	51	22	<5	6	30	<5	8	5	28	0	5	10	<5	9	23	17	0	11	<5	0	0	<5	0	0	0	<5	0	0	<5	<5	<5	0	0
Paediatrics ST4	<5	49	13	<5	5	20	0	5	8	20	0	<5	7	<5	8	23	9	0	9	<5	<5	<5	<5	0	0	<5	<5	0	<5	<5	0	<5	<5	<5
Palliative Medicine ST3	<5	10	6	6	7	5	<5	0	<5	<5	0	0	49	<5	<5	<5	<5	0	<5	<5	<5	0	0	0	0	0	0	0	0	27	<5	0	0	0
Plastic Surgery ST3	0	24	7	16	11	6	<5	<5	<5	<5	<5	5	60	<5	24	13	33	0	<5	<5	6	5	<5	0	0	0	<5	<5	<5	28	<5	8	<5	9
Public Health Medicine ST1	12	90	36	13	33	105	12	6	19	20	5	13	440	35	55	38	56	0	<5	<5	<5	<5	<5	0	0	<5	0	0	<5	68	<5	5	<5	<5
Rehabilitation Medicine ST3	<5	<5	13	<5	5	13	0	<5	<5	6	0	<5	9	<5	<5	5	9	0	<5	<5	0	0	<5	0	<5	0	<5	0	<5	0	0	<5	<5	<5
Sport and Exercise Medicine ST3	0	5	6	0	<5	<5	0	0	<5	0	0	0	11	<5	<5	<5	<5	0	<5	<5	0	<5	0	0	0	0	0	0	0	0	<5	0	0	0
Trauma and Orthopaedic Surgery ST3	<5	113	44	12	23	31	<5	<5	8	21	<5	11	177	7	41	45	58	<5	17	12	6	6	<5	<5	0	0	<5	<5	<5	98	<5	8	5	12
Urology ST3	6	22	13	10	9	18	<5	<5	<5	<5	0	<5	59	8	12	20	12	<5	9	<5	0	<5	<5	<5	<5	<5	0	0	0	23	<5	<5	<5	<5

For peer review only

For peer review only

STROBE Statement—checklist of items that should be included in reports of observational studies

	Item No.	Recommendation	Page No.
**Title and abstract**	1	(*a*) Indicate the study's design with a commonly used term in the title or the abstract	2
		(*b*) Provide in the abstract an informative and balanced summary of what was done and what was found	2
		**Introduction**	
Background/rationale	2	Explain the scientific background and rationale for the investigation being reported	3-5
Objectives	3	State specific objectives, including any prespecified hypotheses	4-5
		**Methods**	
Study design	4	Present key elements of study design early in the paper	5-7
Setting	5	Describe the setting, locations, and relevant dates, including periods of recruitment, exposure, follow-up, and data collection	5-6
Participants	6	(*a*) *Cohort study*—Give the eligibility criteria, and the sources and methods of selection of participants. Describe methods of follow-up<br>*Case-control study*—Give the eligibility criteria, and the sources and methods of case ascertainment and control selection. Give the rationale for the choice of cases and controls<br>*Cross-sectional study*—Give the eligibility criteria, and the sources and methods of selection of participants	5-6
		(*b*) *Cohort study*—For matched studies, give matching criteria and number of exposed and unexposed<br>*Case-control study*—For matched studies, give matching criteria and the number of controls per case	NA
Variables	7	Clearly define all outcomes, exposures, predictors, potential confounders, and effect modifiers. Give diagnostic criteria, if applicable	5-7
Data sources/ measurement	8*	For each variable of interest, give sources of data and details of methods of assessment (measurement). Describe comparability of assessment methods if there is more than one group	5-7
Bias	9	Describe any efforts to address potential sources of bias	5-7
Study size	10	Explain how the study size was arrived at	6

Continued on next page

For peer review only - http://bmjopen.bmj.com/site/about/guidelines.xhtml

Quantitative variables	11	Explain how quantitative variables were handled in the analyses. If applicable, describe which groupings were chosen and why	5-7	
Statistical methods	12	(*a*) Describe all statistical methods, including those used to control for confounding	6-7	
		(*b*) Describe any methods used to examine subgroups and interactions	6-7	
		(*c*) Explain how missing data were addressed	5-7	
		(*d*) *Cohort study*—If applicable, explain how loss to follow-up was addressed<br>*Case-control study*—If applicable, explain how matching of cases and controls was addressed<br>*Cross-sectional study*—If applicable, describe analytical methods taking account of sampling strategy	NA	
		(*e*) Describe any sensitivity analyses	NA	
**Results**				
Participants	13*	(a) Report numbers of individuals at each stage of study—eg numbers potentially eligible, examined for eligibility, confirmed eligible, included in the study, completing follow-up, and analysed	7-13	
		(b) Give reasons for non-participation at each stage	NA	
		(c) Consider use of a flow diagram	NA	
Descriptive data	14*	(a) Give characteristics of study participants (eg demographic, clinical, social) and information on exposures and potential confounders	7-9	
		(b) Indicate number of participants with missing data for each variable of interest	7-9	
		(c) *Cohort study*—Summarise follow-up time (eg, average and total amount)	NA	
Outcome data	15*	*Cohort study*—Report numbers of outcome events or summary measures over time	7-9	
		Case-control study—Report numbers in each exposure category, or summary measures of exposure		
		Cross-sectional study—Report numbers of outcome events or summary measures		
Main results	16	(*a*) Give unadjusted estimates and, if applicable, confounder-adjusted estimates and their precision (eg, 95% confidence interval). Make clear which confounders were adjusted for and why they were included	7-9	country of qualification (UK versus Non-UK) is included as a covariate
		(*b*) Report category boundaries when continuous variables were categorized	NA	
		(*c*) If relevant, consider translating estimates of relative risk into absolute risk for a meaningful time period	NA	

Continued on next page

For peer review only - http://bmjopen.bmj.com/site/about/guidelines.xhtml

Other analyses	17	Report other analyses done—eg analyses of subgroups and interactions, and sensitivity analyses	7-9
**Discussion**			
Key results	18	Summarise key results with reference to study objectives	9-10
Limitations	19	Discuss limitations of the study, taking into account sources of potential bias or imprecision. Discuss both direction and magnitude of any potential bias	10-11
Interpretation	20	Give a cautious overall interpretation of results considering objectives, limitations, multiplicity of analyses, results from similar studies, and other relevant evidence	9-15
Generalisability	21	Discuss the generalisability (external validity) of the study results	9-15
**Other information**			
Funding	22	Give the source of funding and the role of the funders for the present study and, if applicable, for the original study on which the present article is based	17

*Give information separately for cases and controls in case-control studies and, if applicable, for exposed and unexposed groups in cohort and cross-sectional studies.

Note: An Explanation and Elaboration article discusses each checklist item and gives methodological background and published examples of transparent reporting. The STROBE checklist is best used in conjunction with this article (freely available on the Web sites of PLoS Medicine at http://www.plosmedicine.org/, Annals of Internal Medicine at http://www.annals.org/, and Epidemiology at http://www.epidem.com/). Information on the STROBE Initiative is available at www.strobe-statement.org.

For peer review only
